# Excessive collagen type VII mediates pleural fibrosis via increasing extracellular matrix stiffness

**DOI:** 10.1172/JCI188822

**Published:** 2025-10-16

**Authors:** Qian Li, Xin-Liang He, Shuai-Jun Chen, Qian Niu, Tan-Ze Cao, Xiao-Lin Cui, Zi-Heng Jia, He-De Zhang, Xiao Feng, Ye-Han Jiang, Li-Mei Liang, Pei-Pei Cheng, Shi-He Hu, Liang Xiong, Meng Wang, Hong Ye, Wan-Li Ma

**Affiliations:** 1 Department of Pathophysiology, School of Basic Medicine; and; 2 Department of Respiratory and Critical Care Medicine, Union Hospital; Tongji Medical College, Huazhong University of Science and Technology, Wuhan, China.; 3 Department of Respiratory and Critical Care Medicine, Wuhan Pulmonary Hospital, Wuhan, China.

**Keywords:** Cell biology, Inflammation, Pulmonology, Collagens, Extracellular matrix, Fibrosis

## Abstract

The interaction between cells and extracellular matrix (ECM) has been recognized in the mechanism of fibrotic diseases. Collagen type VII (collagen VII) is an ECM component that plays an important role in cell-ECM interaction, particularly in cell anchoring and maintenance of ECM integrity. Pleural mesothelial cells (PMCs) drive inflammatory reactions and ECM production in pleura. However, the role of collagen VII and PMCs in pleural fibrosis was poorly understood. In this study, collagen VII protein was found to be increased in pleura of patients with tuberculous pleural fibrosis. Investigation of cellular and animal models revealed that collagen VII began to increase at an early stage in the pleural fibrotic process. Increase of collagen VII occurred ahead of collagen I and α-SMA in PMCs and pleura of animal models. Inhibition of collagen VII by mesothelial cell–specific deletion of collagen VII gene (*Wt1-Cre^+^ Col7a1^fl/fl^*) attenuated mouse experimental pleural fibrosis. Finally, it was found that excessive collagen VII changed collagen conformation, which resulted in elevation of ECM stiffness. Elevation of ECM stiffness activated integrin/PI3K-AKT/JUN signaling and promoted more ECM deposition, as well as mediated pleural fibrosis. In conclusion, excessive collagen VII mediated pleural fibrosis via increasing ECM stiffness.

## Abstract 2

Excessive collagen type VII mediated pleural fibrosis via increasing extracellular matrix stiffness and activating integrin/PI3K-AKT/JUN signaling.

## Graphical abstract

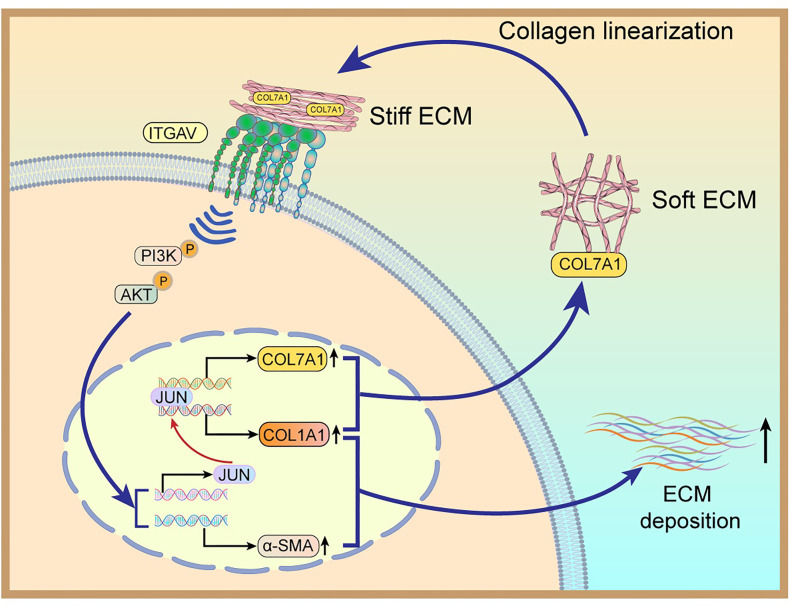

## Introduction

Fibrotic diseases are characterized by excessive deposition of extracellular matrix (ECM). The global incidence of fibrotic diseases is increasing. Most fibrotic diseases lack adequate treatment, and increase health burdens and economic burdens on humans (1). Pleural fibrosis is distinctive because of its association with early pleural inflammation, subsequent tissue thickening, and fibrosis (2). Pleural mesothelial cells (PMCs) play the pivotal role in driving inflammatory reactions and ECM production in pleura (3). However, the detailed role of PMCs in pleural fibrosis remained unclear. Pleural fibrosis is irreversible, resulting in lung entrapment and respiratory complications. There is no effective medication for pleural fibrosis. In many cases, pleurectomy is the only treatment option (4, 5). Thus, exploration of the mechanisms of pleural fibrosis is urgently needed.

In the past decade, alteration in ECM mechanical properties, particularly increased matrix stiffness, was recognized to be involved in the mechanism of tissue fibrosis (6, 7). Conformational changes of ECM proteins are highly correlated with ECM stiffness. Collagen becomes tight after linearization, which results in increase of ECM stiffness (8–11). In addition to providing structural support, ECM regulates cellular signaling pathways that are crucial for cell and tissue homeostasis. Cells respond to mechanical stress of ECM through cell-ECM mechanotransduction, influencing cellular behaviors and phenotype (12). Collagen type VII (collagen VII), an ECM component, plays an important role in cell-ECM interaction, particularly in cell anchoring and maintenance of ECM integrity (13, 14). Mutations in the *COL7A1* gene, which encodes collagen VII protein, have been identified as the primary cause of the disease recessive dystrophic epidermolysis bullosa. Reduced collagen VII results in decreased anchoring between skin cells and ECM, leading to recurrent blistering and inflammation in skin tissue (15–17). The cell-ECM interaction mechanism has been recognized in fibrotic diseases such as cardiac fibrosis, liver fibrosis, and pulmonary fibrosis (18–22). Many cell types, including mesenchymal stem cells, fibroblasts, epithelial cells, endothelial cells, and macrophages, have been identified as mechanosensors of ECM (23–26). PMCs are active cells characterized by microvilli-covered apical surface and well-defined basal surface (26). PMCs should exhibit unique features in pleural mechanosensing mechanisms. However, the roles of PMCs and collagen VII in pleural fibrosis were poorly understood.

Tuberculous pleurisy is one of the most common respiratory diseases in developing countries. It is also the main cause of pleural fibrosis in these countries. Thus, cellular models, animal models, and clinical samples from patients with tuberculous pleurisy were used in the current study. The aim of this study was to investigate changes of collagen VII in PMCs and understand their role in pleural fibrosis.

## Results

### Collagen VII protein increased in pleura of patients with tuberculous pleural fibrosis.

**Figure 1:**
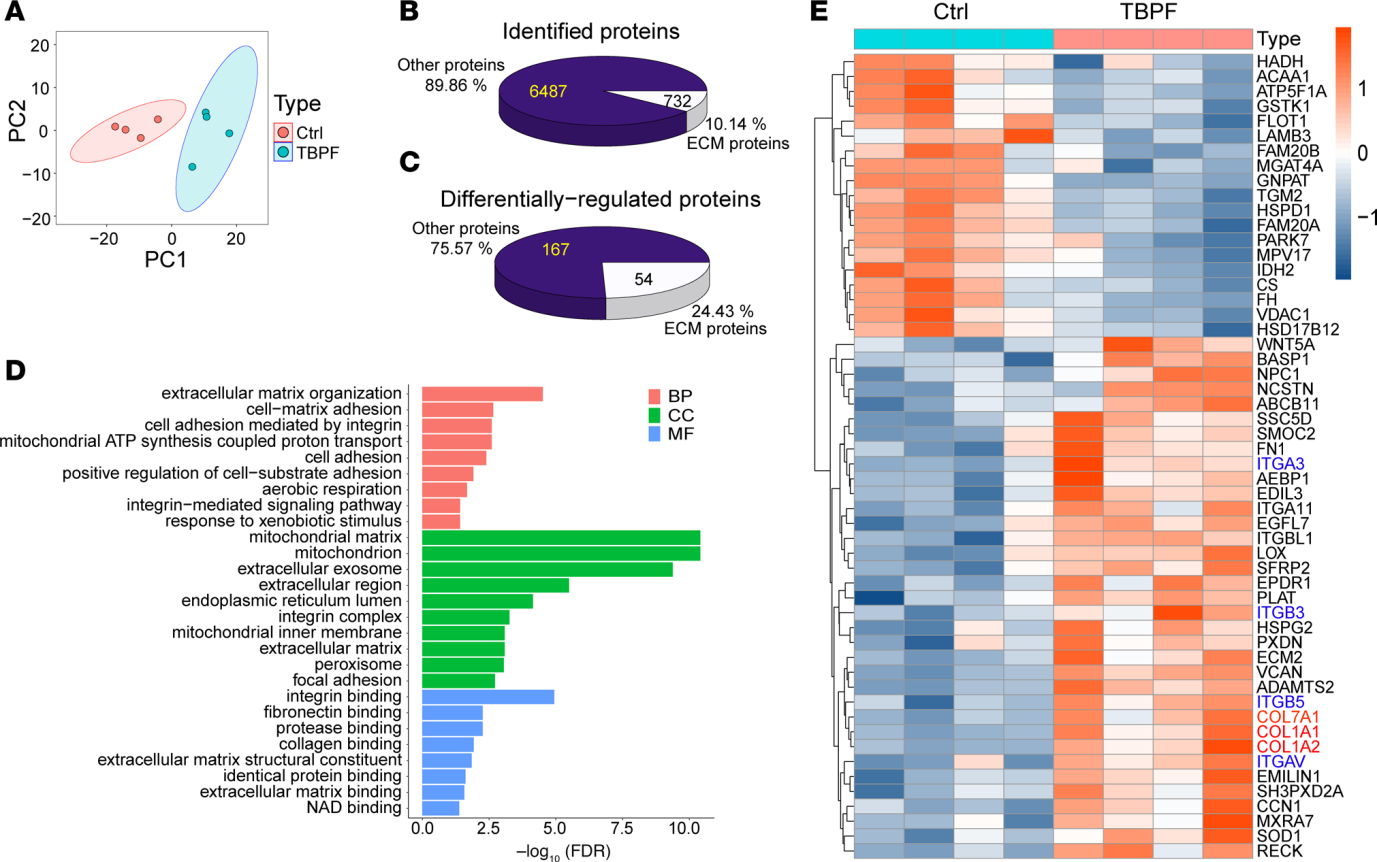
Collagen VII protein increased in pleura of patients with tuberculous pleural fibrosis. (**A**) Principal component analysis of proteomic expression in human pleural tissues was performed by tandem mass tag–based proteomics. Ctrl, control subjects; TBPF, patients with tuberculous pleural fibrosis. *n* = 4. (**B**) Pie chart of extracellular matrix (ECM) protein proportion in total proteins. (**C**) Number and proportion of differentially expressed proteins between 2 groups. (**D**) Gene Ontology enrichment analysis of differentially expressed proteins. (**E**) Heatmap shows 54 differentially expressed ECM proteins (FDR 1%, fold change > 2).

Using tandem mass tag–based proteomic analysis, we performed principal component analysis in pleural tissues from 4 patients with tuberculous pleural fibrosis (TBPF) and 4 control subjects with normal pleura. Different protein clustering in pleural tissues was distinguished between the TBPF and the control group (Figure 1A). We identified 7,219 proteins, with 732 (10.14%) classified as ECM proteins (Figure 1B). There were 221 proteins that expressed marked difference between the 2 groups. Notably, 54 (24.43%) of these 221 differentially expressed proteins were ECM proteins (Figure 1C). Gene Ontology enrichment analysis indicated that ECM organization and cell-matrix adhesion was the main different biological process between the 2 groups (Figure 1D). It revealed upregulation of fibrosis-associated proteins including collagen VII (COL7A1) and collagen I (COL1A1, COL1A2) in pleura of TBPF compared with controls (Figure 1E). Masson’s trichrome staining showed that collagen deposition was substantially elevated in pleura of TBPF compared with controls (Supplemental Figure 1, A and B; supplemental material available online with this article; https://doi.org/10.1172/JCI188822DS1). Moreover, immunohistochemistry staining (Supplemental Figure 1, C and D) and immunofluorescence staining (Supplemental Figure 1, E and F) further verified excessive expression of collagen VII and collagen I in pleura of TBPF. Western blot and quantitative real-time PCR analysis also reinforced increased collagen VII in TBPF pleura (Supplemental Figure 1, G–I).

### Collagen VII increased ahead of collagen I during the pleural fibrotic process.

**Figure 2:**
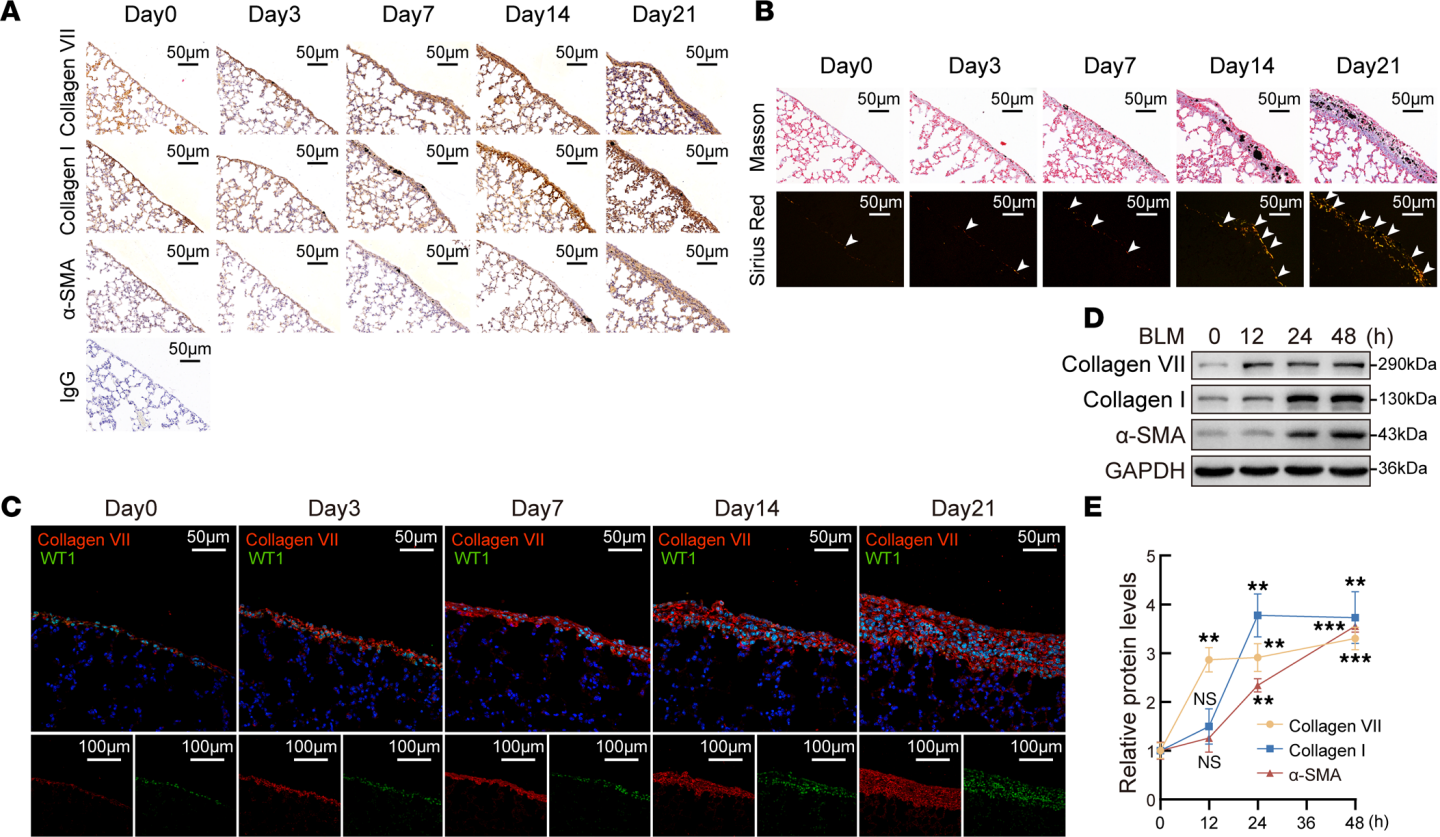
Collagen VII increased from early stage during pleural fibrotic process. Mouse pleural fibrosis model was induced by intrapleural injection of bleomycin (BLM) plus carbon particles. Lungs with pleural tissues were collected at days 3, 7, 14, and 21 (*n* = 4). The indicated staining was performed using lung and visceral pleura sections. (**A**) Representative immunohistochemistry staining for collagen VII, collagen I, and α-SMA. (**B**) Representative images of Masson’s trichrome staining (top) and polarized light microscopy of Sirius red staining (bottom). (**C**) Representative immunofluorescence staining for WT1 (green), collagen VII (red), and their colocalization in mouse pleural tissues. Nuclei were counterstained with DAPI (blue). Scale bars: 50 μm (upper); 100 μm (lower). (**D** and **E**) PMCs were treated with BLM (0.2 μg/mL) for 12, 24, or 48 hours, after which collagen VII, collagen I, and α-SMA protein was detected by Western blotting. (**D**) Representative images of Western blots. (**E**) Line graph shows changes of proteins according to **D**. Results are expressed as mean ± SD. Statistical significance was determined using 1-way ANOVA. *n* = 3. **P < 0.01, ***P < 0.001; ns, not significant compared with 0 hours.

To investigate dynamic changes of collagen VII expression during the pleural fibrotic process, a mouse model was made by intrapleural administration of bleomycin (BLM) combined with carbon particles. As shown in Figure 2, A and B, after the inflammatory process at days 3 and 7, collagen deposition and fibrosis were obviously established at day 21. Notably, immunohistochemistry and immunofluorescence staining revealed that upregulation of collagen VII started at day 3, with substantial increases at day 7 and maintenance at a high level to day 21. In contrast, collagen I and α-SMA were upregulated from day 7 to day 21 (Figure 2, A and C, and Supplemental Figure 2).

In the in vitro experiments, firstly collagen VII was detected in PMCs, lung fibroblasts, and alveolar and bronchial epithelial cells. As shown in Supplemental Figure 3, A–I, the basic level of collagen VII expression in alveolar and bronchial epithelial cells was very low, and there was no change even when the cells were treated with the pleural fibrotic inducer BLM or tuberculous pleural effusion (TBPE). In contrast, collagen VII was highly expressed in primary human PMCs and rat PMCs, and moderately expressed in lung fibroblasts. After treatment with BLM or TBPE, collagen VII increased more in rat PMCs than in lung fibroblasts (Supplemental Figure 3, E and F). Moreover, coimmunofluorescence staining of collagen VII and WT1 (a marker of mesothelial cells) revealed that collagen VII was predominantly expressed in PMCs in mouse lung tissues (Figure 2C) and human samples (Supplemental Figure 1E). Thus, PMCs were considered as a primary source of collagen VII and investigated in subsequent experiments. As shown in Figure 2, D and E, collagen VII increased earlier than collagen I and α-SMA in BLM-treated PMCs. It was confirmed again that BLM or TBPE treatment induced increases of collagen VII expression in primary human PMCs (Supplemental Figure 3, G–J) and primary rat PMCs (Supplemental Figure 4, A–H). However, interestingly, TGF-β1 did not change collagen VII protein or mRNA expression, suggesting a regulatory mechanism independent of TGF-β1 signaling (Supplemental Figure 4, A and I–K).

These findings indicated that collagen VII increased from an early stage and ahead of collagen I during the pleural fibrotic process.

### Inhibition of collagen VII in PMCs attenuated pleural fibrosis induced by bacillus Calmette-Guérin, TBPE, or BLM plus carbon particles.

To explore the role of collagen VII in pleural fibrosis, primary rat PMCs were treated with *Col7a1* siRNA and recombinant collagen VII protein. As shown in Supplemental Figure 5, A–E, *Col7a1* siRNA was designed, and inhibited BLM- and TBPE-induced cell proliferation. BLM and TBPE induced upregulation of collagen I and α-SMA, and these were inhibited by *Col7a1* siRNA (Supplemental Figure 5, F–M). On the contrary, treatments with serial concentrations of recombinant collagen VII protein induced a dose-dependent upregulation of collagen I and α-SMA in the cells (Supplemental Figure 6). *COL7A1* siRNA and recombinant collagen VII protein also changed expression of collagen I and α-SMA in primary human PMCs (Supplemental Figure 7).

**Figure 3:**
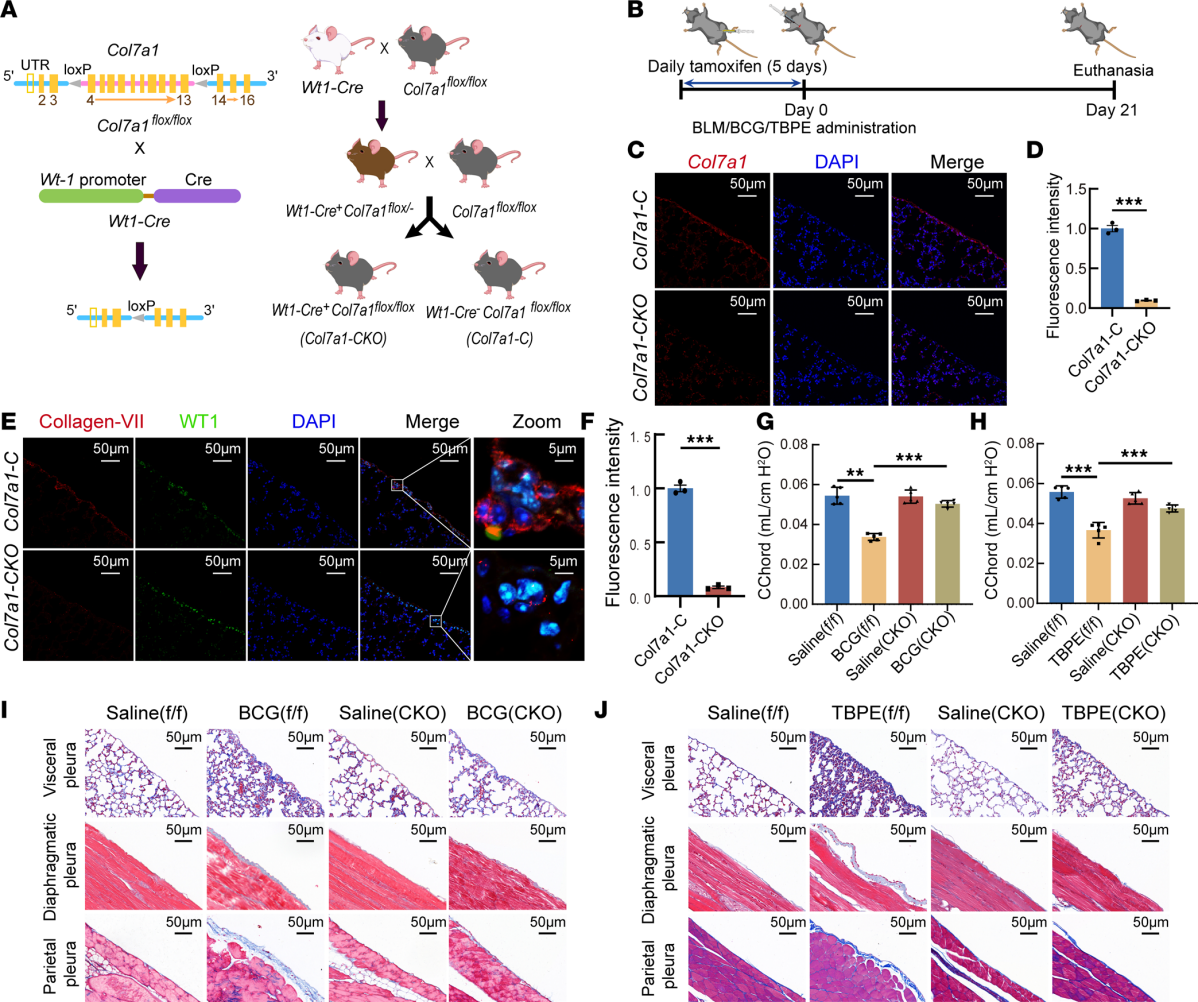
Mesothelial cell–specific deletion of collagen VII attenuated pleural fibrosis induced by bacillus Calmette-Guérin or tuberculous pleural effusion. (**A**) *Col7a1^fl/fl^* mice were generated using the CRISPR/Cas9 system by insertion of two *loxP* sequences into introns flanking *Col7a1* exons. Upon Cre-mediated gene deletion, a stop codon was introduced, resulting in a non-functional collagen VII protein. Cross-breeding *Col7a1^fl/fl^* mice with WT1-Cre transgenic mice produced mice with mesothelium-specific *Col7a1* knockout, *Wt1-Cre^+^ Col7a1^fl/fl^*. (**B**) Schematic of the making of the pleural fibrosis model in *Wt1-Cre^+^ Col7a1^fl/fl^* mice by intrapleural injection of bacillus Calmette-Guérin (BCG) or tuberculous pleural effusion. (**C**) Representative RNA-FISH staining of *Col7a1* mRNA in pleural tissues of *Wt1-Cre^+^ Col7a1^fl/fl^* and *Wt1-Cre*^–^ *Col7a1^fl/fl^* mice. *Col7a1* transcripts were visualized in red; nuclei were counterstained with DAPI (blue). Scale bars: 50 μm. (**D**) Bar graph showing fluorescence intensity of red color according to **C**. (**E**) Tissue sections were stained with anti–N-terminal collagen VII antibody (red) and WT1 (green), and nuclei were counterstained with DAPI (blue). Scale bars: 50 μm; 5 μm in zoom panels. (**F**) Bar graph showing fluorescence intensity of red color according to **E**. (**D** and **F**) Results are expressed as mean ± SD. Statistical significance was determined by unpaired 2-tailed Student’s *t* tests. *n* = 3. ***P < 0.001. (**G**–**J**) Pleural fibrosis models were made by intrapleural injection of BCG or tuberculous pleural effusion in control and *Wt1-Cre^+^ Col7a1^fl/fl^* mice. Before lung and pleural tissues were taken at day 21 after injection, lung function tests were performed. (**G** and **H**) Lung function test results. Cchord, chord compliance. Results are presented as mean ± SD. Statistical significance was determined using 1-way ANOVA. *n* = 5. **P < 0.01, *** *P* < 0.001. (**I** and **J**) Representative images of Masson’s trichrome staining of visceral, parietal, and diaphragmatic pleura. Scale bars: 50 μm. TBPE, tuberculous pleural effusion; CKO, conditional knockout.

At the same time, mice with mesothelial cell–specific deletion of *Col7a1* (*Wt1-Cre^+^ Col7a1^fl/fl^*) were generated and used in pleural fibrosis models (Figure 3, A and B). RNA-FISH, quantitative PCR (qPCR), and immunofluorescence colocalization collectively confirmed collagen VII depletion in pleura of mouse models (Figure 3, C–F, and Supplemental Figure 8, A–C). Mouse pleural fibrosis models were made by bacillus Calmette-Guérin (BCG), TBPE, or BLM plus carbon particles. Knockout of *Col7a1* in mesothelial cells restored the declined mouse lung chord compliance (Figure 3, G and H, and Supplemental Figure 8D). Data from Masson’s trichrome staining, immunohistochemistry, immunofluorescence, and Sirius red staining all indicated marked attenuation of pleural fibrosis in *Wt1-Cre^+^ Col7a1^fl/fl^* mice with downregulated collagen I and α-SMA (Figure 3, I and J, Supplemental Figure 8, E–I, and Supplemental Figures 9 and 10). These data suggested that inhibition of collagen VII in PMCs attenuated pleural fibrosis.

### Excessive collagen VII increased ECM stiffness by regulating collagen conformation.

**Figure 4:**
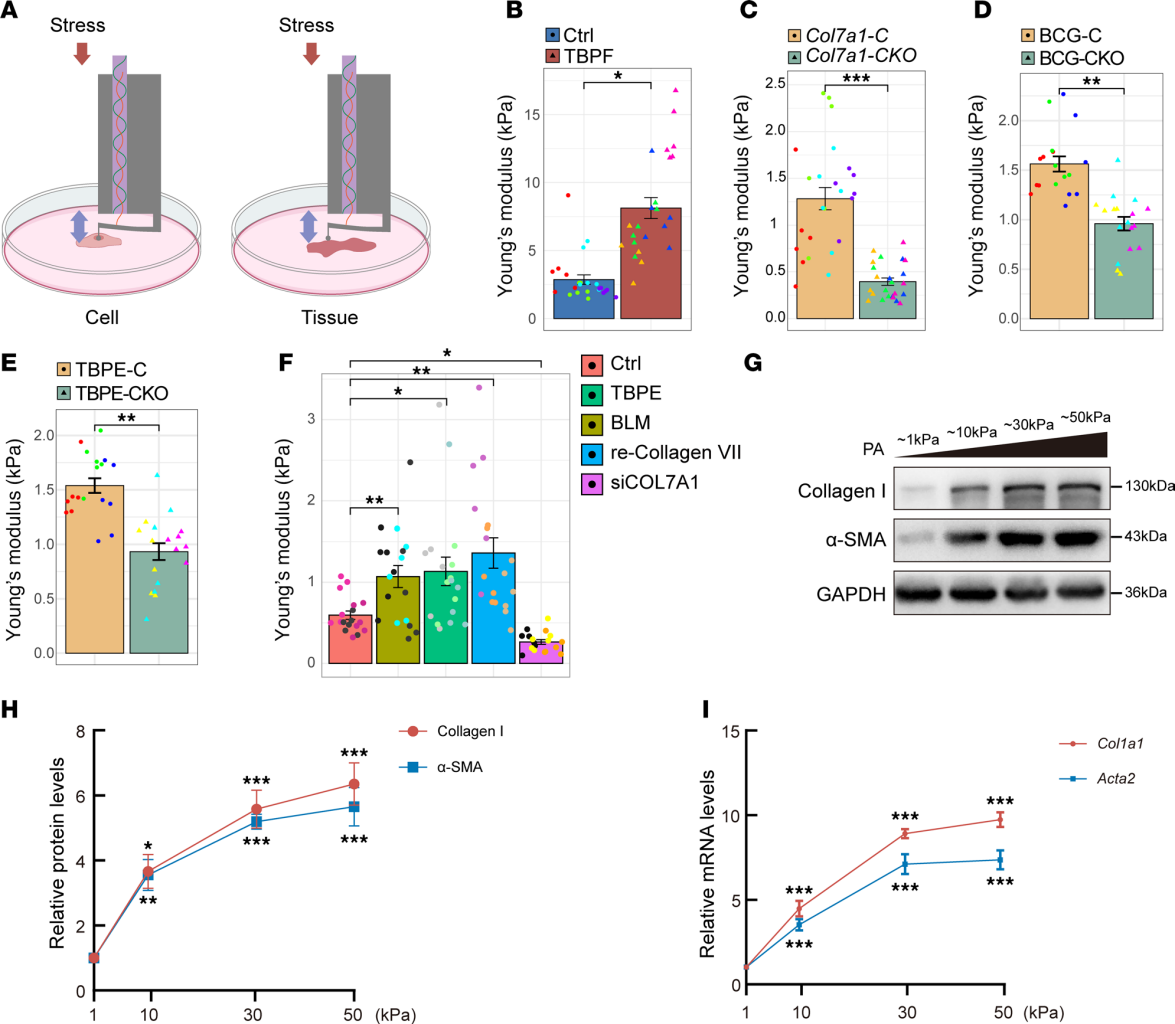
Collagen VII increased cell and tissue stiffness. (**A**) Schematic of the nanoindenter measuring the Young’s modulus of cells and tissues. (**B**) Young’s modulus analysis of pleural tissue from patients and control. *n* = 4, one color indicating one subject. (**C**) Young’s modulus analysis of pleural tissue from *Wt1-Cre^+^ Col7a1^fl/fl^* mice or control mice. *n* = 4. (**D** and **E**) Young’s modulus analysis in pleural tissue of *Wt1-Cre^+^ Col7a1^fl/fl^* mice and control mice treated with BCG (**D**) or TBPE (**E**). *n* = 3. (**B**–**E**) Data are expressed as mean ± SD. Statistical significance was determined using unpaired 2-tailed Student’s *t* tests. *P < 0.05, **P < 0.01, ***P < 0.001. (**F**) Nanoindentation measurement of cellular Young’s modulus in cultured PMCs treated with BLM (0.2 μg/mL), TBPE (5%), recombinant collagen VII protein (1 μg/mL), and siRNA of collagen VII. *n* = 3. (**G**–**I**) Effect of substrate stiffness on collagen I and α-SMA expression in cultured PMCs. As described in Methods, polyacrylamide hydrogels with defined stiffness (1, 10, 30, and 50 kPa) were prepared, then PMCs were cultured for 24 hours. Cells were harvested for Western blotting and real-time qPCR. (**G**) Representative images of Western blots of collagen I and α-SMA protein. (**H**) Line graphs showing protein changes according to **G**. *n* = 3. (**I**) Line graphs showing changes of *Col1a1* and *Acta2* mRNA. *n* = 3. (**F**, **H**, and **I**) Data are expressed as mean ± SD. Statistical significance was determined using 1-way ANOVA. *P < 0.05, **P < 0.01, ***P < 0.001. CKO, conditional knockout; re-Collagen VII, recombinant collagen VII protein; siCOL7A1, *Col7a1* siRNA.

As an anchoring fibril, collagen VII is located in the basement membrane. To understand interactions of collagen VII with ECM and the underlying role in mechanical stress, the Young’s modulus of pleural tissue was assessed using a bio-nanoindenter (Figure 4A). As shown in Figure 4B, stiffness of pleural tissue was substantially elevated in TBPF patients compared with controls. On the contrary, *Col7a1* knockout in mesothelial cells decreased tissue stiffness in control mice (Figure 4C). Further investigation showed that collagen VII deletion in *Wt1-Cre^+^ Col7a1^fl/fl^* mice prevented increases of tissue stiffness induced by BCG or TBPE (Figure 4, D and E). In the in vitro experiments, BLM, TBPE, and recombinant collagen VII protein increased cellular rigidity in PMCs. On the contrary, the cellular rigidity decreased in PMCs treated with *Col7a1* siRNA (Figure 4F). Substrate polyacrylamide hydrogels, which had serial levels of stiffness in medium, increased collagen I and α-SMA expression in cultured primary rat PMCs (Figure 4, G–I). At the same time, polydimethylsiloxane (PDMS) gels were used in treatment for primary rat PMCs, and similar results were obtained (Supplemental Figure 11).

**Figure 5:**
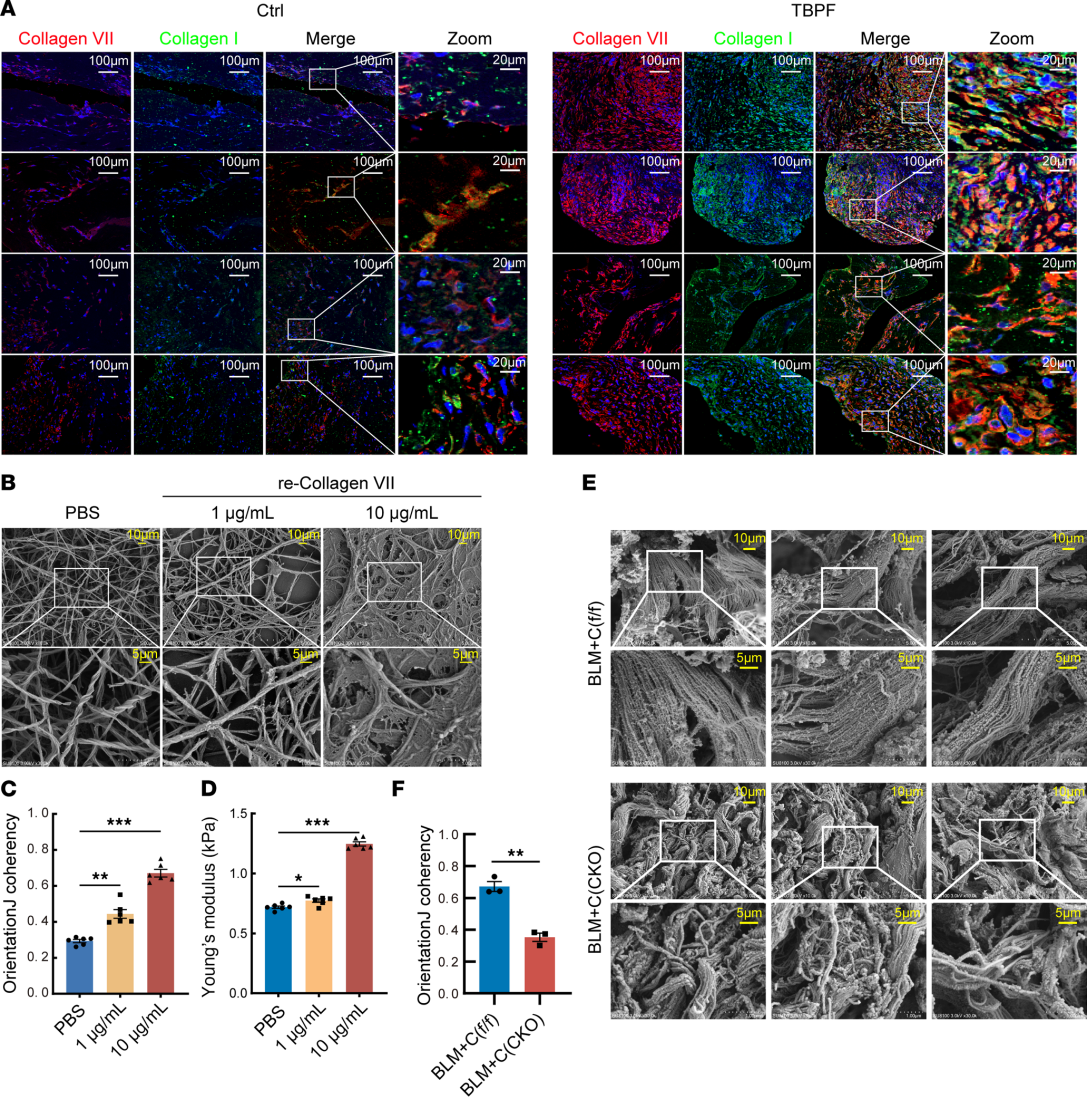
Excessive collagen VII induced collagen I linearization. (**A**) Immunofluorescence staining was performed on human pleural tissue sections to reveal collagen VII (red) and collagen I (green). Colocalization is shown in yellow. Nuclei were counterstained with DAPI (blue). Scale bars: 100 μm; 20 μm in zoom panels. Ctrl, control subjects; TBPF, patients with tuberculous pleural fibrosis. *n* = 4. (**B**–**D**) Collagen I was treated with recombinant collagen VII for 24 hours in the absence of cells. (**B**) Representative electron microscopy images of alterations in the triple-helix structure. Top row: original magnification, ×10,000; bottom row: original magnification, ×30,000. Scale bars: 10 μm (top), 5 μm (bottom). (**C**) Bar graphs showing coherency changes according to **B**. (**D**) Young’s modulus analysis to show changes of stiffness. (**C** and **D**) Data are expressed as mean ± SD. Statistical significance was determined using 1-way ANOVA. *n* = 6. *P < 0.05, **P < 0.01, ***P < 0.001. (**E** and **F**) Pleural fibrosis models were made using *Cre^+^ Col7a1^fl/fl^* mice and control mice by intrapleural injection of BLM plus carbon particles as described in Supplemental Figure 8. (**E**) Representative images of electron microscopy scan of pleural tissues. Scale bars: 10 μm or 5 μm. (**F**) Bar graphs showing coherency changes according to **E**. *n* = 3. Data are expressed as mean ± SD. Statistical significance was determined by unpaired 2-tailed Student’s *t* tests. **P < 0.01. re-Collagen VII, recombinant collagen VII protein.

To understand why excessive collagen VII increases ECM stiffness, immunofluorescence staining was performed on clinical pleural samples to reveal the location of collagen VII and collagen I. As shown in Figure 5A, increased collagen VII and collagen I were interlaced in the fibrotic pleura of TBPF. Collagen VII directly interacted with collagen I with binding site at the vWF2 domain of *Col7a1*. Then an in vitro experiment was performed to investigate the effect of collagen VII on collagen I. As shown in Figure 5, B and C, collagen I was directly treated with recombinant collagen VII; scanning electron microscopy revealed that collagen VII induced changes of collagen I conformation, which presented as loosening of the triple-helical structure, even linearization. Mechanical stiffness assays revealed that collagen VII increased the stiffness of collagen I gels (Figure 5D). In mouse models, scanning electron microscopy showed collagen fiber linearization and tight organization in fibrotic pleural tissue, but these were prevented by collagen VII knockout (Figure 5, E and F).

These results indicated that excessive collagen VII promoted collagen fiber linearization and increased ECM stiffness.

### Excessive collagen VII mediated pleural fibrosis through integrin in TBPF.

**Figure 6:**
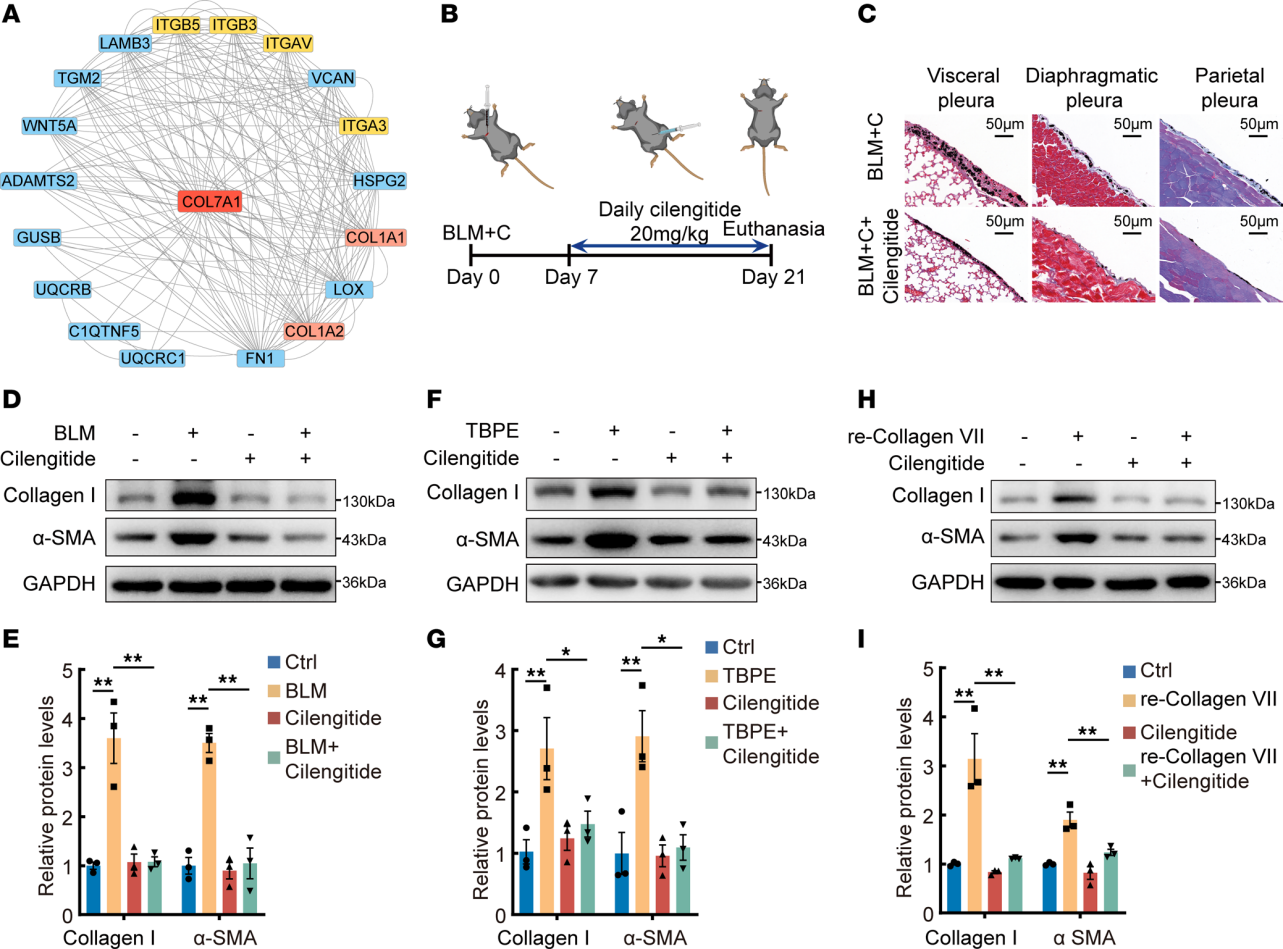
ITGAV siRNA and cilengitide prevented increases of collagen I and α-SMA in vivo and in vitro. (**A**) Protein-protein interaction network analysis based on differentially expressed proteins identified by tandem mass tag analysis in Figure 1A. Key molecular interaction proteins with collagen VII are shown. (**B**) Flowchart of protocol. Mice with pleural fibrosis induced by BLM plus carbon particles were treated with the integrin inhibitor cilengitide. Cilengitide (20 μg/g) was administered intraperitoneally daily from day 7 to day 21 after BLM plus carbon injection. *n* = 5. (**C**) Representative images of Masson’s trichrome staining of visceral, parietal, and diaphragmatic pleura from model mice and cilengitide-treated model mice. Scale bars: 50 μm. (**D**–**I**) PMCs were treated with BLM (0.2 μg/mL), TBPE (5%), and recombinant collagen VII (1 μg/mL) with or without cilengitide (10 μg/mL) for 24 hours, after which collagen I and α-SMA proteins were detected by Western blotting. (**D**, **F**, and **H**) Representative images of Western blots of collagen I and α-SMA protein. (**E**, **G**, and **I**) Bar graphs showing changes of protein according to **D**, **F**, and **H**, respectively. Data are expressed as mean ± SD. Statistical significance was determined using 1-way ANOVA. *n* = 3. *P < 0.05, **P < 0.01. re-Collagen VII, recombinant collagen VII protein.

Increasing evidence highlighted a central role of integrins in mediating cell-ECM interactions, such as ECM adhesion and mechanotransduction (27, 28). Based on protein-protein interaction analysis in pleural tissues from TBPF and control subjects, multiple integrin proteins (ITGAV, ITGA3, ITGB3, and ITGB5) and collagen I were identified interacting with collagen VII (Figure 6A). In the above experiments, ITGAV, ITGA3, ITGB3, and ITGB5 were also found to be increased in TBPF (Figure 1E). Previous studies revealed a crucial role of α_v_β_3_ and α_v_β_5_ integrin in fibrosis. α_v_β_3_ consists of ITGAV (α_v_ integrin) and ITGB3 (β_3_ integrin), and α_v_β_5_ consists of ITGAV and ITGB5 (β_5_ integrin). Thus, the role of ITGAV was investigated in pleural fibrosis here.

First, animal model experiments were performed. Cilengitide, a cyclized Arg-Gly-Glu–containing (RGD-containing) pentapeptide, selectively blocks activation of the α_v_β_3_ and α_v_β_5_ integrins (29, 30). Thus, cilengitide was selected to use in vivo. As shown in Figure 6, B and C, mouse pleural fibrosis was attenuated by cilengitide. Then cultured PMCs were treated with *Itgav* siRNA to inhibit ITGAV protein as well as decrease integrin. As shown in Supplemental Figure 12, A–F, BLM, TBPE, and recombinant collagen VII substantially increased collagen I and α-SMA, and these were inhibited by *Itgav* siRNA in PMCs. Subsequently, PMCs were treated with cilengitide. Cilengitide also prevented BLM-, TBPE-, and recombinant collagen VII–induced increase of collagen I and α-SMA protein (Figure 6, D–I). mRNA levels of *Col1a1* and *Acta2* had changes similar to those of their proteins in PMCs treated with *Itgav* siRNA or cilengitide (Supplemental Figure 12, G–L).

These data suggested that collagen VII mediated ECM deposition and pleural fibrosis through integrin.

### PI3K-AKT signaling occurred downstream of integrin in mediation of pleural fibrosis by collagen VII.

**Figure 7:**
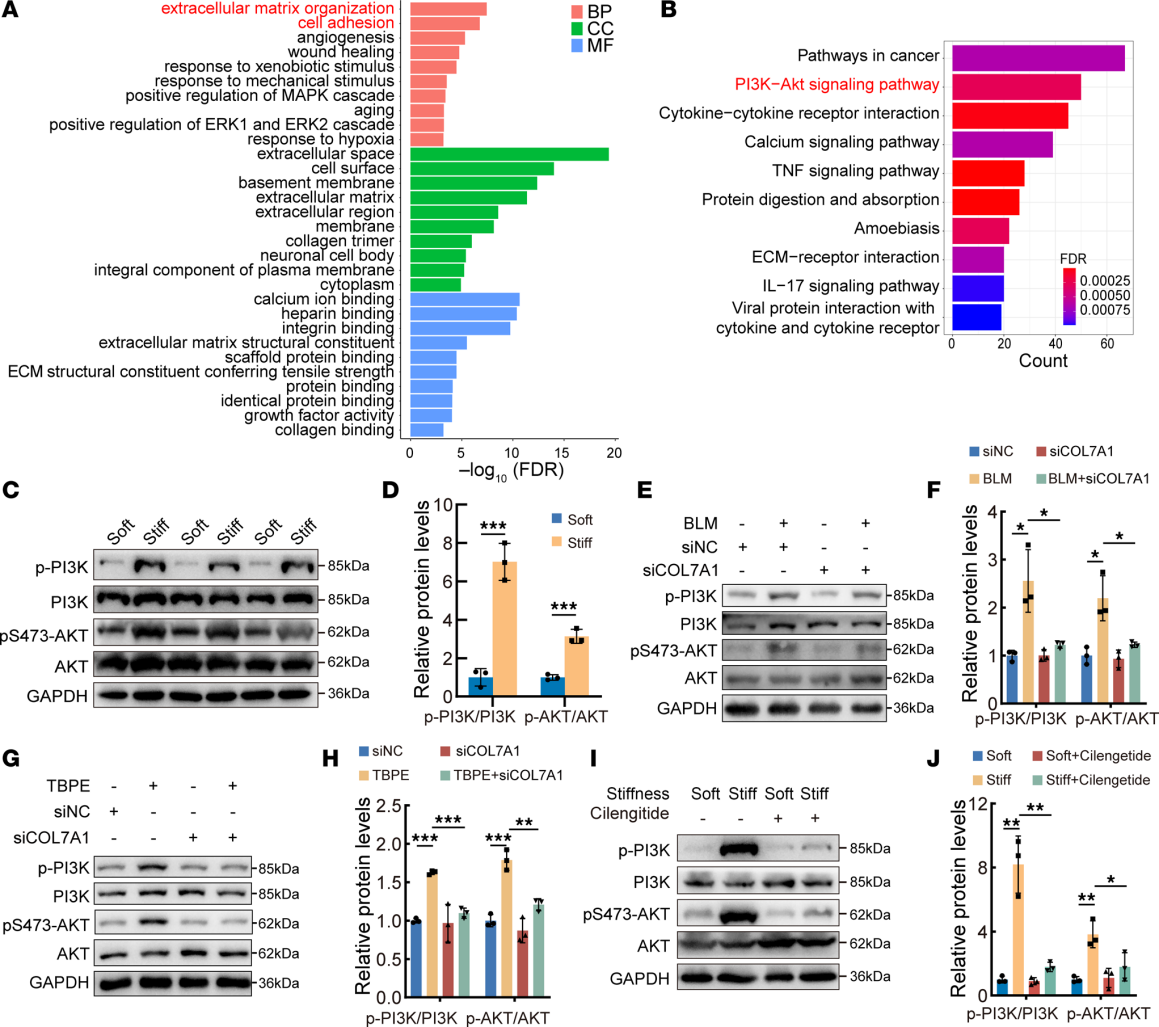
Increased ECM stiffness activated integrin/PI3K-AKT signaling. (**A**) Gene Ontology analysis of differentially expressed genes from RNA-Seq between *Col7a1* siRNA and control groups in PMCs. BP: biological process; CC: cellular component; MF: molecular function. (**B**) KEGG pathway analysis based on data of differentially expressed genes identified by RNA-Seq. (**C** and **D**) PI3K-AKT pathway signaling protein expressions in PMCs cultured in substrates of different stiffness. As described in Methods, soft and stiff substrate was made by polyacrylamide hydrogels with elastic moduli of about 1 kPa (soft) and about 30 kPa (stiff). PMCs were cultured in medium with these soft or stiff substrates for 24 hours, then harvested for Western blotting. (**D**) Bar graphs showing changes of proteins according to **C**. Data are expressed as mean ± SD. Statistical significance was determined by unpaired Student’s *t* tests. *n* = 3. ***P < 0.001. (**E**–**H**) Effect of *Col7a1* siRNA on PI3K-AKT signals in PMCs. After transfection of control siRNA or *Col7a1* siRNA for 36 hours, PMCs were cultured with or without BLM and TBPE for 24 hours, then harvested for Western blotting. (**F** and **H**) Bar graphs showing changes of proteins according to **E** and **G**. Data are expressed as mean ± SD. Statistical significance was determined by 1-way ANOVA. *n* = 3. *P < 0.05, **P < 0.01, ***P < 0.001. (**I** and **J**) Effect of cilengitide on PI3K-AKT signals in PMCs. Soft and stiff substrate in PMC-cultured medium was made in the same manner as in **C**. PMCs were cultured with or without cilengitide (10 μg/mL) for 24 hours, then harvested for Western blotting. (**J**) Bar graphs showing changes of proteins according to **I**. Data are expressed as mean ± SD. Statistical significance was determined by 1-way ANOVA. *n* = 3. *P < 0.05, **P < 0.01. siNC, control siRNA; siCOL7A1, *Col7a1* siRNA.

To further understand the mechanism of collagen VII in pleural fibrosis, RNA-Seq analysis was conducted in PMCs that were treated by silencing of *Col7a1*. Gene Ontology enrichment analysis confirmed again a close relationship between collagen VII and ECM (Figure 7A). Kyoto Encyclopedia of Genes and Genomes (KEGG) analysis revealed that differentially expressed genes after *Col7a1* silencing were mainly enriched in the PI3K-AKT signaling pathway (Figure 7B). As shown in Supplemental Figure 13, TBPE and BLM activated the PI3K-AKT pathway in cultured rat PMCs. Furthermore, PI3K-AKT signaling was more active in PMCs cultured in stiff polyacrylamide hydrogels versus soft ones (Figure 7, C and D). Knockdown of *Col7a1* inhibited BLM- or TBPE-induced PI3K-AKT pathway activation (Figure 7, E–H). Stiff polyacrylamide hydrogels and recombinant collagen VII protein activated the PI3K-AKT pathway, and this was prevented by the integrin inhibitor cilengitide (Figure 7, I and J, and Supplemental Figure 14, A and B). However, collagen VII knockdown failed to inhibit activation of PI3K-AKT signaling when PMCs had been cultured in stiff polyacrylamide hydrogels (Supplemental Figure 14, C and D). To further confirm the role of PI3K-AKT signaling in mediation of pleural fibrosis by collagen VII, PDMS gels were also used. As shown in Supplemental Figure 15, stiff PDMS gels activated PI3K-AKT signaling as stiff polyacrylamide hydrogels did.

These findings indicated that excessive collagen VII activated the integrin/PI3K-AKT signaling pathway after increasing ECM stiffness.

### Collagen VII and JUN formed a positive-feedback loop in mediating pleural stiffness.

**Figure 8:**
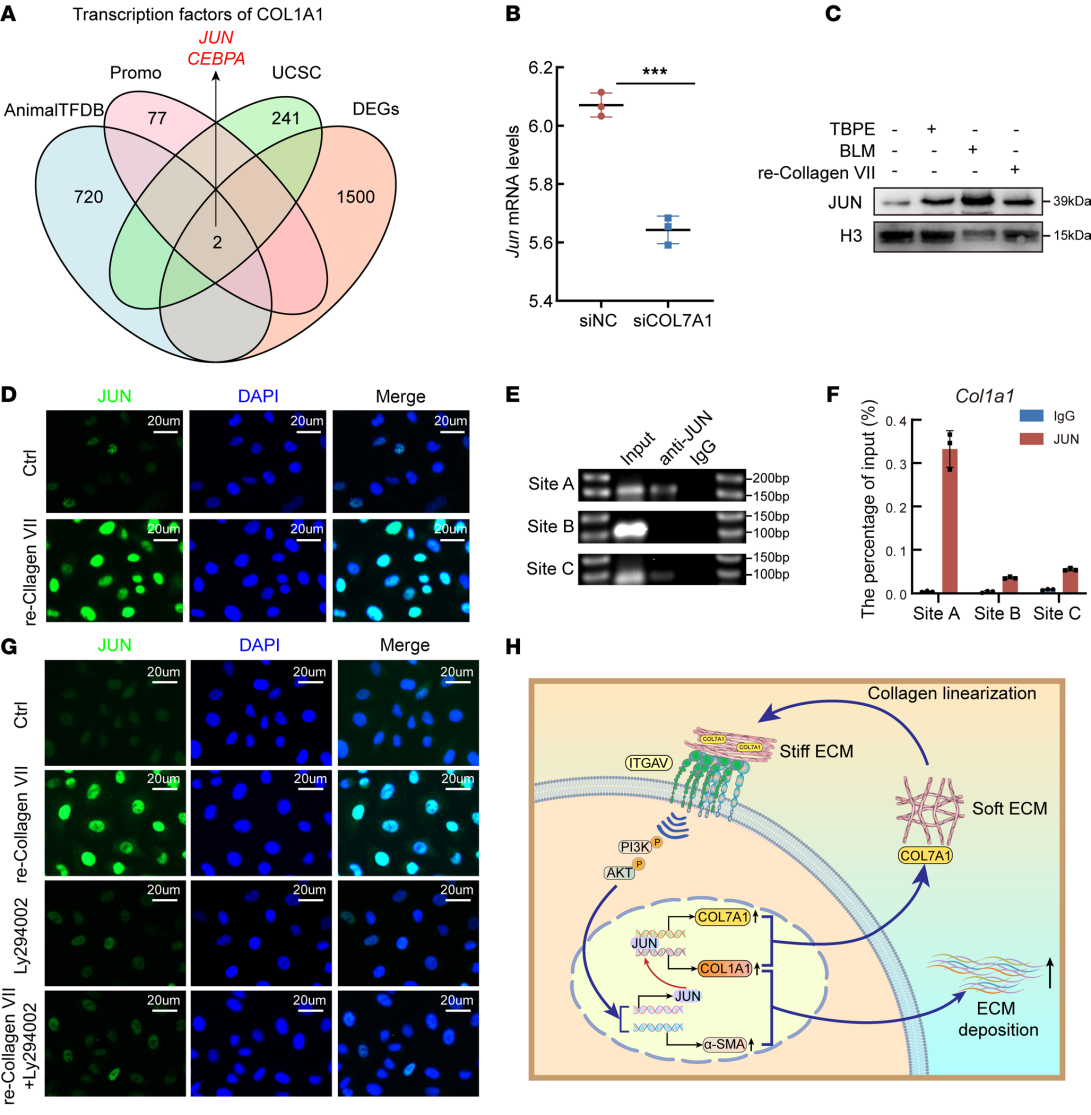
Excessive collagen VII mediated pleural fibrosis via ECM stiffness and integrin/PI3K-AKT/JUN. (**A**) Venn diagram showing prediction of transcription factors for *Col1a1*. Based on data of differentially expressed genes after collagen VII knockdown (shown in Figure 6A), transcription factors for *Col1a1* were predicted using 3 transcription factor databases (AnimalTFDB, PROMO, and UCSC). (**B**) Bar graph showing JUN mRNA expression level changes in PMCs treated with *Col7a1* siRNA. Data are expressed as mean ± SD. Statistical significance was determined by unpaired 2-tailed Student’s *t* tests. *n* = 3. ***P < 0.001. (**C**) Immunoblotting analysis of nuclear JUN expression in PMCs treated with TBPE (5%), BLM (0.2 μg/mL), and recombinant collagen VII protein (1 μg/mL) for 24 hours. (**D**) Immunofluorescence staining showing JUN distribution and expression in PMCs treated with recombinant collagen VII protein. Scale bars: 20 μm. (**E**) Agarose gel electrophoresis of DNA products from chromatin immunoprecipitation (ChIP). (**F**) ChIP-qPCR and luciferase assays to evaluate JUN-binding activity on *Col1a1* promoter. *n* = 3. (**G**) Immunofluorescence analysis to reveal effect of recombinant collagen VII and LY294002 (inhibitor of PI3K-AKT) on JUN expression. Scale bars: 20 μm. (**H**) Schematic illustrating role of collagen VII in pleural fibrosis. re-Collagen VII, recombinant collagen VII protein; siNC, control siRNA; siCOL7A1, *Col7a1* siRNA.

To further understand the underlying mechanism, based on results of RNA-Seq analysis in PMCs treated with *Col7a1* siRNA, three databases of transcription factor (AnimalTFDB, PROMO, and UCSC) were used to predict downstream of integrin/PI3K-AKT signaling. The prediction result indicated that 2 transcription factors, JUN and CEBPA, were involved in collagen I expression regulated by collagen VII/integrin/PI3K-AKT signaling (Figure 8A). Previous studies implicated that JUN was downstream of PI3K-AKT signaling (31). Here, downregulation of JUN expression in PMCs was confirmed when *Col7a1* was knocked down (Figure 8B). Therefore, it was necessary to investigate whether collagen VII/integrin/PI3K-AKT regulates *Col1a1* transcription via JUN. As shown in Figure 8C, Western blotting analysis of nuclear protein extraction was performed, revealing that TBPE, BLM, and recombinant collagen VII protein upregulated JUN expression. The immunofluorescence results also indicated that recombinant collagen VII protein activated JUN (Figure 8D). Subsequently, results of ChIP and dual-luciferase reporter gene assays confirmed that JUN was a transcription factor of collagen I, and its binding sites on the promoter of *Col1a1* were identified (Figure 8, E and F, and Supplemental Figure 16). Inhibition of the PI3K-AKT pathway attenuated the upregulation of JUN induced by recombinant collagen VII (Figure 8G). Moreover, inhibition of JUN prevented up-expression of collagen I and α-SMA induced by recombinant collagen VII protein or stiff matrix (Supplemental Figure 17). Interestingly, predictive analysis indicated that JUN was a transcription factor of *Col7a1* in multiple databases. Here, experiments with ChIP-qPCR and dual-luciferase reporter gene assays confirmed that JUN was a transcriptional factor of *Col7a1* (Supplemental Figure 18). Thus, collagen VII promoted JUN production via the integrin/PI3K-AKT pathway, while JUN in turn facilitated collagen VII transcription, forming a positive-feedback loop in mediating ECM deposition and pleural fibrosis (Figure 8H).

## Discussion

In this study, firstly, collagen VII protein was found to be increased in pleurae of patients with TBPF. Next, results from cellular and animal models revealed that collagen VII began to increase at an early stage during the pleural fibrotic process. Increase of collagen VII was earlier than that of collagen I and α-SMA. Inhibition of collagen VII attenuated experimental pleural fibrosis induced by BCG, TBPE, or BLM plus carbon particles. Then the underlying mechanism was studied. Excessive collagen VII changed collagen conformation, which resulted in increasing of ECM stiffness. Increased ECM stiffness activated the integrin/PI3K-AKT/JUN signaling pathway, which led to more ECM deposition as well as pleural fibrosis. Taken together, these results suggest that excessive collagen VII increased ECM stiffness, which contributed to pleural fibrosis.

At the beginning of this study, using tandem mass tag–based proteomic analysis, we tried to find changes of protein components in fibrotic pleurae from TBPF patients. Not surprisingly, consistent with our previous findings (32), collagen I substantially increased. Notably, upregulation of collagen VII and integrin proteins caught our attention. Collagen VII, which is responsible for anchoring fibril assembly, bridges epidermis and dermis to the basement membrane (13, 14). Mutations in *COL7A1*, which encodes collagen VII, led to recessive dystrophic epidermolysis bullosa (16). However, to our knowledge, reports concerning the role of collagen VII in pleural fibrosis were scarce. Our study revealed dynamic changes of collagen VII, collagen I, and α-SMA in PMCs and pleural fibrosis. Collagen VII changed before collagen I and α-SMA, which indicated that collagen VII should be an active participant in, rather than an end result of, fibrotic remodeling in driving pleural fibrosis progression. TGF-β1 is a classical profibrotic factor across various fibrotic conditions, such as pulmonary, pleural, hepatic, and cardiac fibrosis (33). As inducers of pleural fibrosis, BLM and TBPE promoted collagen VII synthesis in PMCs. Meanwhile, TGF-β1 did not change collagen VII expression in the cells. These results indicated that collagen VII mediated pleural fibrosis independent of the TGF-β1 signaling pathway.

We used 3 methods — BLM plus carbon particles, TBPE, and BCG-induced — to make pleural fibrosis models in the current study. Inhibition of collagen VII prevented pleural fibrosis in these 3 models. This suggested that collagen VII had a common effect in pleural fibrosis that resulted from different etiological factors. So far, very limited effective cure for pleural fibrosis has been available (34); collagen VII should be a potential target for treatment of pleural fibrosis.

In this study, we disclosed that collagen VII mediated pleural fibrosis via increasing pleural tissue stiffness, which resulted from collagen loosening its triple-helical structure and linearization. Maintenance of the elasticity of collagen fibers, especially collagen I, depends on this triple-helical structure. A previous study confirmed that collagen VII directly interacts with collagen I (35). Wegener et al. reported that collagen VII directly bound with collagen I at the vWF2 domain of *Col7a1* (36). ECM protein binding to collagen I results in its conformation change. Jia and colleagues disclosed that tumor cell–secreted matricellular protein WISP1 (CCN4) directly bound to collagen I to promote its linearization in vitro (in the absence of cells) and in vivo in tumors (11). Similarly, the current study found that recombinant collagen VII changed collagen I conformation, which presented as loosening of the triple-helical structure, even becoming linearization. Linearized collagen I became flat and organized dense fibers, which increased ECM stiffness. Increased stiffness and disorganized ECM changed the mechanical properties of ECM, which triggered cell-ECM crosstalk signaling; these changes progressively impeded tissue function and ultimately caused fibrosis (37–40). Furthermore, researchers proposed that ECM was a driver of progressive fibrosis (41). Parker et al. reported that fibrotic ECM activated a profibrotic positive-feedback loop between fibroblasts and aberrant ECM in idiopathic pulmonary fibrosis (IPF); interrupting this loop may be a strategy for fibrosis treatment (42).

Next, we explored how increased pleural stiffness transmitted molecular signaling. Increased ECM stiffness indicates increased tissue mechanical tension. Based on integrin-based adhesions, the mechanical tension is transmitted from ECM to intracellular cytoskeleton and cellular signaling pathways (43, 44). Wu and colleagues reported that elevated mechanical tension activated a TGF-β signaling loop in AT2 cells, which drove progressive lung fibrosis (45). We elucidated that increased tissue stiffness activated signaling pathways through integrins. Integrins are known as receptors of ECM that bridge cells and ECM. The main protein of integrins, ITGAV, played a crucial role in ECM rigidity sensing and adaptation (46, 47). Targeting ITGAV was a strategy in preventing ECM abnormalities (48, 49).

Using RNA-Seq analysis, we found that PI3K-AKT signaling was a downstream pathway of integrin in pleural fibrosis. Matrix stiffening was reported to activate the PI3K-AKT pathway (50). The PI3K-AKT signaling pathway regulates a variety of biological processes, balances cytoskeletal forces, and mediates cytoskeletal remodeling and epithelial-mesenchymal transition (51–53). c-JUN is a component of the heterodimeric AP-1 transcription factor, which is regarded as an oncoprotein, and promotes fibrosis in liver, skin, and lung; a c-JUN N-terminal kinase (JNK) inhibitor has been used in clinical trials for patients with pulmonary fibrosis (54–57). Fu et al. found that increased mechanical pressure induced JUN expression (31). Previous research reported that the PI3K-AKT pathway was a positive regulator of JUN expression in epithelial-mesenchymal transition (58), which played an important role in ECM deposition. In the current study, we confirmed that JUN was downstream of PI3K-AKT signal in ECM deposition and pleural fibrosis. All this research supported our idea that the integrin-ITGAV/PI3K-AKT/JUN pathway played a key role in mediation of pleural fibrosis by collagen VII. Thus, excessive collagen VII increased ECM stiffness and subsequently triggered integrin/PI3K-AKT/JUN signaling, which contributed to ECM deposition and aggravated pleural fibrosis.

Like pleural fibrosis, IPF is a classical fibrotic lung disease, a refractory disease characterized by subpleural damage and fibrosis. The subpleural lung damage that often accompanies pleural injury indicated that PMCs were involved in these disorders (59). Furthermore, ECM deposition in IPF is similar to that in pleural fibrosis (60). Thus, upregulated collagen VII in PMCs should contribute to lung subpleural damage and fibrosis in IPF. We speculated that our current findings about collagen VII in pleural fibrosis would be helpful in exploring the pathogenesis of IPF.

Last, it is worth noting that our study has several limitations. (a) Excessive collagen VII was found in pleurae from TBPF, but we did not uncover why and how collagen VII increased. (b) We did additional experiments to learn whether there is a membrane receptor on PMCs for collagen VII, and did not find one (data not shown). Further studies using more sensitive or targeted approaches are needed to clarify whether potential direct interactions between PMCs and collagen VII exist. (c) In our study, proteomic analysis identified ITGAV, ITGA3, ITGB3, and ITGB5 as the key integrins associated with collagen VII. Collagen VII promoted pleural fibrosis via increasing the stiffness of ECM, while α_v_β_3_ and α_v_β_5_ have been reported to sense ECM mechanical forces and transduce mechanical signals into the cell (61–64). Cilengitide selectively blocks activation of α_v_β_3_ and α_v_β_5_ integrins rather than all α_v_ integrins (29, 30). Thus, we selected cilengitide. However, we do not rule out the possibility that other factors played a role through cilengitide during the process. (d) WT1 expression is predominantly restricted to mesothelial cells, and WT1-Cre has been widely used to target PMCs. However, the expression of WT1 in other cell types such as fibroblasts and myofibroblasts cannot be fully excluded. This may have a slight influence on our results.

In summary, our study elucidated the role of collagen VII in pleural fibrosis. Excessive collagen VII mediated pleural fibrosis via increasing ECM stiffness, which activated the integrin/PI3K-AKT/JUN signaling pathway. Further translational and clinical research is expected to explore therapeutic strategy in human pleural fibrosis.

## Methods

### Sex as a biological variable.

Our study examined male and female human subjects and animals, and similar findings are reported for both sexes.

### Reagents and antibodies.

Recombinant human TGF-β1 protein and PI3K kinase inhibitor (LY294002) were purchased from R&D Systems. Recombinant human collagen VII protein was sourced from Abmart (CG424928S). The ChIP assay kit was obtained from Active Motif, and the dual-luciferase reporter gene assay kit was acquired from Vazyme. Cilengitide and tamoxifen were sourced from MedChemExpress, and carbon particles from Mitsubishi Chemical Corp. Bleomycin (BLM) was obtained from Haerbin Laibotong Pharmaceutical Co. Ltd. The antibodies used in the experiments were as follows: anti–collagen VII antibody (ab309143, Abcam, and 19799-1-Ap, Proteintech), anti–collagen I antibody (AF7001, Affinity), anti–α-SMA antibody (PAB59619, Bioswamp), anti–TGF-β1 antibody (21898-1-AP, Proteintech), anti-PI3K antibody (4249, Cell Signaling Technology), anti–p-PI3K antibody (4228, Cell Signaling Technology), anti-AKT antibody (9272, Cell Signaling Technology), anti–p-AKT antibody (4060, Cell Signaling Technology), anti-WT1 antibody (ab89901, Abcam), anti-JUN antibody (24909-1-AP, Proteintech), anti-ITGAV antibody (27096-1-AP, Proteintech), anti-GAPDH antibody (60004-1, Proteintech), anti-H3 antibody (T56587, Abmart), and IgG antibody (10283-1-AP, Proteintech).

### Clinical samples.

Pleural tissue samples from patients with tuberculous pleurisy and normal pleural tissue resected from lung adenocarcinoma in patients were obtained with informed consent from the patients. Clinical information is presented in Supplemental Table 1. The diagnostic criteria for tuberculous pleurisy included the identification of *Mycobacterium tuberculosis* in pleural fluid or the presence of caseating granulomas in closed pleural biopsy specimens in the absence of evidence of any other granulomatous diseases. Tuberculous pleurisy was diagnosed according to Light’s criteria. Definitive diagnosis of tuberculous pleurisy was based on the detection of *M*. *tuberculosis* in pleural fluid or pleural tissue, or demonstration of caseating granulomas in pleural biopsy. The TBPF pleural tissues used in this study were obtained from patients initially admitted with suspected tuberculous pleurisy and pleura thickening based on clinical symptoms and chest CT imaging findings. The TBPF pleural tissues were pleura samples obtained by pleural biopsy using medical thoracoscopy. All patients had not received anti-tuberculosis treatment prior to the pleural biopsy. The pleural tissues of TBPF used in this study were finally diagnosed as tuberculous pleurisy according to above criteria. Pleural tissues in the control group were obtained from patients with lung adenocarcinoma and pleural effusion. These pleural tissues were obtained by pleural biopsy using medical thoracoscopy in the same manner as the TBPF pleural tissues. These tissues were hyperplastic pleura without fibrosis or manifestations of tumor metastasis, which was confirmed by pathological examination, and served as control pleura. This study was approved by the Human Ethics Committee of Union Hospital, Tongji Medical College, Huazhong University of Science and Technology.

### Tandem mass tag.

Total proteins were extracted from fresh pleura, and protein quality was assessed. Qualified samples were subjected to trypsin digestion and tandem mass tag labeling separately. The labeled peptides were then mixed in equal amounts to form a composite. After desalting, the composite underwent fractionation using high-pH reversed-phase HPLC, producing 12 distinct peptide fractions. Each fraction was subjected to nano-HPLC reversed-phase chromatography and mass spectrometric detection. Protein identification and quantitative analysis were performed using Proteome Discoverer/MaxQuant search software (Thermo Fisher Scientific). Quantitative results were standardized and subjected to statistical analysis to identify differentially expressed proteins for subsequent bioinformatics analysis.

### Generation of Wt1-Cre^+^ Col7a11^fl/fl^ mice and making of the mouse pleural fibrosis model.

*Col7a1^fl/fl^* mice were generated by Cyagen Biosciences (Suzhou, China) using the CRISPR/Cas9 system. Two *loxP* sequences were inserted into the introns flanking *Col7a1* exons. *Wt1-Cre* transgenic mice were obtained from The Jackson Laboratory. Wild-type C57BL/6 mice were also sourced from The Jackson Laboratory. The *Wt1-Cre^+^ Col7a1^fl/fl^* (*Col7a11*-conditional-knockout [*Col7a11*-CKO]) mice were crossed with *Col7a1^fl/fl^* mice to specifically delete collagen VII in mesothelial cells, with their *Wt1-Cre^–^ Col7a1^fl/fl^* (*Col7a1*-control [*Col7a1*-C]) littermates serving as controls. *Col7a1*-C and *Col7a1*-CKO mice (8–10 weeks old) were anesthetized with 1% pentobarbital sodium (60 mg/kg) and injected intrapleurally with BLM and carbon particles, bacillus Calmette-Guérin (BCG), or TBPE (5 μL/g) in the right pleural cavity, 3–5 mm to the right of the sternum at the fifth intercostal space, while control mice received an equal volume of saline. All mice were euthanized on day 21 after lung function measurements. Mice were housed in specific pathogen–free facilities at Tongji Medical College with a 12-hour light/dark cycle. All experimental procedures were approved by the Animal Care and Use Committee of Tongji Medical College, Huazhong University of Science and Technology.

### Experimental procedure for cilengitide treatment.

Mice were subjected to intraperitoneal injections of cilengitide (20 mg/kg/d; dissolved in PBS) starting on day 7 after the mouse model was made. The cilengitide injections were administered once daily for a duration of 2 weeks, continuing until euthanasia at day 21. Control animals received an equivalent volume of PBS following the same injection schedule as the cilengitide treatment group. The treatments were carried out at a consistent time each day to minimize potential variations due to circadian effects. At the conclusion of the treatment period, all animals were humanely euthanized in accordance with institutional guidelines.

### Second-harmonic generation imaging method.

Second-harmonic generation (SHG) imaging was performed using a Leica 2-photon microscope with a ×25 water immersion objective. The tissues were excited with a laser wavelength of 880 nm, and the SHG signal was detected by 420 to 460 nm range. This setup allowed for high-resolution imaging of collagen deposition in TBPF tissues. The imaging was conducted under standard conditions, and all images were processed and analyzed using Leica software. Representative SHG images are shown Supplemental Figure 19.

### Isolation and identification of primary human PMCs.

Fresh pleural effusion was first filtered through sterile gauze to remove debris, followed by centrifugation at 1,500*g* for 6 minutes at 4°C to collect the cell pellet. The pellet was resuspended in RPMI 1640 or DMEM/F-12 medium supplemented with 10% fetal bovine serum (FBS) and 1% penicillin-streptomycin, and seeded into uncoated culture flasks. The cells were then incubated at 37°C in a 5% CO_2_ atmosphere. After 24 hours, the culture medium was replaced to remove non-adherent cells. To enhance purity, cells were cultured under low-serum conditions (2% FBS) with the addition of epidermal growth factor (EGF; 10 ng/mL) to promote mesothelial cell proliferation. Once the cells reached an appropriate confluence, their identity was confirmed by immunofluorescence staining, with the markers calretinin and WT1 (Supplemental Figure 20). This isolation and culture protocol was consistent with our previously published methods (32, 65).

### Isolation of primary rat PMCs.

Primary rat PMCs were isolated from 4-week-old male Sprague-Dawley rats (80–120 g). Under sterile conditions, the thoracic cavity was exposed, and 5 mL of 1 mg/mL protease dissolved in RPMI 1640 medium was injected into the pleural space. The carcass was gently shaken and incubated at 4°C for 12 hours, followed by 30 minutes at 37°C with 0.1 mg/mL DNase I (Roche). The cell suspension was collected and centrifuged at 300*g* for 5 minutes at 4°C. The pellet was washed with PBS and resuspended in Epithelial Cell Medium–Animal (catalog 4101, ScienCell) supplemented with 10% FBS and 1% penicillin-streptomycin. The cells were cultured at 37°C in a 5% CO_2_ atmosphere for 7 days, with medium changes every 48 hours. Before experiments, cells were passaged using 0.25% trypsin-EDTA containing 0.1% soybean trypsin inhibitor (Gibco) and seeded in RPMI 1640 with 20% FBS. This isolation and culture protocol was consistent with the methods in our previous studies (32, 66, 67).

### Culture and treatment of cells.

Human PMCs (HPMCs), PMCs, human lung fibroblasts (HLFs), and rat lung fibroblasts (RLFs) were isolated as primary cells. The rat alveolar epithelial cell line 6-TN, HEK293T cells, and human bronchial epithelial (HBE) cell lines were obtained from the American Type Culture Collection. HPMCs and PMCs were cultured in RPMI 1640 medium (Gibco) supplemented with 10% FBS. HLFs and HBE and HEK293T cells were cultured in high-glucose DMEM (Gibco) supplemented with 10% FBS (Gibco). 6-TN cells were cultured in RPMI 1640 medium supplemented with 10% FBS. Cells were passaged at a 1:3 ratio, and the medium was changed every 1–2 days. Cells were cryopreserved in cell-specific freezing medium (211072, NEST Biotechnology). Different treatment factors and concentrations for cells were as follows: BLM (0.2 μg/mL); TBPE and transudative pleural effusion (5%); recombinant protein TGF-β1 (10 ng/mL); recombinant protein collagen VII (1 μg/mL); cilengitide (10 μg/mL). All culture media contained 100 U/mL penicillin and 100 μg/mL streptomycin. Cells were maintained at 37°C in a humidified atmosphere containing 5% CO_2_, and cells were starved for 6–8 hours before treatment.

### Detection of tissue stiffness and single-cell stiffness.

Fresh tissues were sliced into 40-μm-thick tissue blocks using a vibratome and then immersed in PBS. Young’s modulus was measured using the Piuma Nanoindenter (Optics11), with probe information indicating stiffness: 0.47 N/m and tip radius 23.5 μm. Individual primary PMCs and polyacrylamide hydrogels were directly measured in culture dishes using the Chiaro Nanoindenter (Optics11), with probe information indicating stiffness: 0.021 N/m and tip radius 3 μm. These measurements were conducted at 37°C under controlled atmosphere (5.0% CO_2_ and constant humidity). Data analysis was performed using the manufacturer-provided software (Data Viewer v2.5.0). The Hertz contact model was applied to determine Young’s modulus from each load-indentation curve, using a constant indentation rate. The contact point of each load-indentation curve was identified using integrated contact fitting software, up to a maximum of 80% of the maximum load. Hertz fitting was applied within the range of contact points between 0 nm and 480 nm.

### Mouse lung function assessment.

To assess mouse lung function, mice were anesthetized with sodium pentobarbital solution for endotracheal intubation and connection to a forced maneuver system (CRFM100, EMMS). Respiration was monitored under spontaneous breathing using whole-body plethysmography and respiratory rate sensors connected to a data acquisition system (EMMS). Lung function parameters were measured, including lung compliance, elasticity, and resistance including chord compliance. The experimental procedures strictly adhered to animal experimental ethics standards, and statistical methods were used for data analysis to compare lung function differences among different groups or post-treatment conditions.

### Scanning electron microscopy.

To ensure consistency in ultrastructural analysis, lung tissue samples for scanning electron microscopy were collected from the right lower lobe of each mouse. A small tissue segment (about 2 × 2 mm) was carefully dissected from the visceral pleura region within 2 mm of the lung periphery. All mice were euthanized under deep anesthesia, and lungs were excised without inflation to avoid distortion of collagen fibrils. Tissue orientation was maintained during dissection and mounting to ensure uniform imaging perspectives. Samples were immediately fixed in 2.5% glutaraldehyde in 0.1 M phosphate buffer (pH 7.4) at 4°C overnight, postfixed in 1% osmium tetroxide, dehydrated through a graded ethanol series, and critical-point-dried. Dried samples were mounted on stubs, sputter-coated with gold, and imaged using a scanning electron microscope.

### Preparation of polyacrylamide hydrogels and polydimethylsiloxane with different stiffness in cell culture.

To make substrates of different stiffness in cultured medium, polyacrylamide hydrogels and polydimethylsiloxane (PDMS) were used. Polyacrylamide hydrogels with defined elastic moduli (~1, ~10, ~30, and ~50 kPa) were fabricated by varying acrylamide/bis-acrylamide concentrations on APTES/DCDMS-treated coverslips. Polymerization was initiated with 0.1% APS and TEMED to generate approximately 100-μm-thick gels, followed by UV cross-linking with collagen I coating (50 μg/mL) (25, 28). The stiffness of polyacrylamide hydrogels was validated by nanoindentation. PDMS (Sylgard 184, Dow Corning) was mixed at base/curing agent ratios of 60:1, 40:1, 20:1, and 10:1 by weight, corresponding to control, soft, moderate, and stiff substrates. Mixtures were cured at 60°C for 12 hours. PMCs were seeded at a density of 1 × 10^5^ cells per well onto each substrate. Cells were cultured in RPMI 1640 medium supplemented with 10% FBS and maintained at 37°C in a humidified incubator with 5% CO_2_. After 24 hours, cells were harvested for downstream analyses.

### Transfection of siRNA and plasmid.

The collagen VII–specific small interfering RNA (siRNA) and corresponding scrambled siRNA were purchased from Ambion. Primary PMCs were seeded in 12-well plates 24 hours before transfection. Transient transfection was performed using Lipofectamine 3000 (Invitrogen, Shanghai, China) when cells reached 60% confluence in RPMI 1640 medium without FBS and antibiotics. Transfection efficiency was evaluated 36 hours after transfection using real-time qPCR or Western blotting. Three different siRNAs targeting collagen VII were tested. *Itgav* and *Jun* siRNAs were used in the same manner as *Col7a1* siRNA. All RNA interference sequences designed in this study are listed in Supplemental Table 2. For JUN plasmid transfection, Neofect (TF201201, Neofect Biotechnologies) was mixed with plasmids according to the instruction manual. After 15 minutes, PMCs and HEK293T cells were transfected with the Neofect-plasmid complexes. Transfected cells were harvested 48 hours after treatment.

### Dual-luciferase reporter gene assay experiment.

Initially, cultured cells were transfected with plasmids containing the firefly luciferase reporter gene and Renilla luciferase gene. Subsequently, cells were treated following experimental requirements and harvested at appropriate time points. The cell lysates were prepared using suitable cell lysis buffer. Firefly luciferase activity was measured by addition of luciferase assay reagent to cell lysates, followed by measurement of Renilla luciferase activity by addition of Renilla luciferase assay reagent in separate split aliquots of cell lysates. Transfection efficiency was normalized by calculation of the ratio of firefly luciferase activity to Renilla luciferase activity. Data analysis was performed based on objectives of the experiment.

### Histological and immunohistochemical analysis.

To determine the cellular localization and changes of collagen VII, collagen I, and α-SMA, PMCs were seeded on sterile glass slides. After different treatments, cells were fixed with 4% paraformaldehyde at room temperature for 40 minutes. Immunostaining was performed using antibodies against collagen VII (dilution 1:100), WT1 (dilution 1:100), collagen I (dilution 1:100), or α-SMA (dilution 1:200) overnight at 4°C, followed by incubation with FITC-conjugated secondary IgG antibodies at room temperature for 1 hour. Cell nuclei in PMCs were stained with DAPI in the dark for 5 minutes. For human and mouse tissues, specific tissues were fixed in PBS containing 4% paraformaldehyde for 48 hours, embedded in paraffin, sectioned, and stained as previously described (1). Fluorescence images were captured using a confocal microscope.

### ChIP assay.

The chromatin immunoprecipitation (ChIP) assay aimed to elucidate the interaction between the transcription factor JUN and the promoters of *COL1A1* and *COL7A1* using the ChIP-IT Express enzymatic kit (53009, Active Motif). After fixation with 1% formaldehyde, PMCs and HEK293T cells were lysed and enzymatically sheared to obtain protein/DNA complexes. Subsequently, immunoprecipitation was performed using antibodies against JUN or IgG. ChIP-enriched DNA fragments were purified, with a portion subjected to qualitative analysis via DNA gel electrophoresis, and the remainder was quantified using real-time qPCR. The primer sequences used for ChIP-qPCR are detailed in Supplemental Table 3.

### Western blot analysis.

After extraction of total protein from fresh tissue or cells, protein concentration was measured using spectrophotometry and then adjusted, followed by the addition of protein loading buffer (L-7102, Biolinkedin) for denaturation. Subsequently, proteins were separated by size using SDS-PAGE electrophoresis. The separated proteins were then transferred onto a PVDF membrane. Nonspecific binding sites were blocked at room temperature for 1 hour using 5% BSA. The membrane was then incubated sequentially with primary antibody overnight at 4°C and with an HRP-conjugated secondary antibody at room temperature for 1 hour. The expression level of the target protein was observed using chemiluminescence. The primary antibody dilutions used in this study were as follows: collagen VII, 1:1,000; collagen I, 1:1,000; α-SMA, 1:1,000; PI3K, 1:1,000; p-PI3K, 1:1,000; AKT, 1:1,000; p-AKT, 1:1,000; JUN, 1:1,000; WT1, 1:1,000; ITGAV, 1:1,000; GAPDH, 1:5,000. Secondary antibody dilutions were 1:10,000 for GAPDH and 1:5,000 for the others.

### Real-time qPCR analysis.

Total RNA from cell or tissue samples was extracted using Trizol, followed by reverse transcription of RNA into corresponding cDNA using Hiscript Q RT SuperMix (Nanjing Novazyme). Subsequently, real-time fluorescence qPCR reactions were performed using Cham Q SYBR qPCR Master Mix (Nanjing Novazyme) and corresponding primers. The relative expression of each target gene was normalized using GAPDH as the reference gene. Specific primer information can be found in Supplemental Table 4.

### RNA deep sequencing (RNA-Seq).

After siRNA interference with PMCs as described above, total RNA from animals was extracted according to the instructions of the TRIzol Reagent (Life Technologies). Subsequently, RNA concentration and purity were measured using the NanoDrop 2000 spectrophotometer (Thermo Fisher Scientific), and RNA integrity was assessed using the Agilent Bioanalyzer 2100 system (Agilent Technologies) with the RNA Nano 6000 assay kit. The initial total amount for library construction of each sample was 1 μg. Following the manufacturer’s instructions, sequencing libraries were generated using the Hieff NGS Ultima Dual-mode mRNA Library Prep Kit for Illumina [Yeasen Biotechnology (Shanghai) Co. Ltd.], and indexes were added to the sequences of each sample. PCR products were purified using AMPure XP magnetic beads (A63880, Beckman Coulter), and library quality was evaluated on the Agilent Bioanalyzer 2100 system. Libraries were sequenced on the Illumina NovaSeq platform, generating paired-end sequences of 150 bp, followed by bioinformatics analysis using the R programming language.

### Statistics.

Data were analyzed using GraphPad Prism software. For comparisons between 2 groups, an unpaired, 2-tailed Student’s *t* test was used. Multiple-group comparisons were conducted using 1-way ANOVA. Gene and protein expression data were normalized using GAPDH as a reference, with stability verified across experimental conditions. Statistical significance was set at *P* less than 0.05. Bioinformatics analysis of RNA-Seq data was performed using R software, including differential expression analysis and pathway enrichment studies with specific packages. All experiments were independently replicated at least 3 times, and results are expressed as mean ± SD and are consistently reported throughout the study.

### Study approval.

Clinical sample collection and related experiments in this study were approved by the Human Ethics Committee of Union Hospital, Tongji Medical College, Huazhong University of Science and Technology (approval number 2022 IEC 043). All animal-related experimental procedures were approved by the Animal Care and Use Committee of Tongji Medical College, Huazhong University of Science and Technology (approval number 20220304).

### Data availability.

All data and sources of materials are included in the article. The RNA-Seq data generated in this study were deposited in the NCBI’s Sequence Read Archive under the BioProject accession number PRJNA1333591. All the values in the figures are provided in the supplemental Supporting Data Values file. Requests for further information and for resources and reagents should be directed to and will be fulfilled by the lead contact, WLM.

## Author contributions

QL, HY, and WLM conceptualized the study. QL, SJC, QN, TZC, XLC, ZHJ, HDZ, XF, YHJ, LML, PPC, SHH, and MW developed methodology. QL, HY, and SJC performed investigation. QL, QN, XLC, and XF performed visualization. HY, WLM, LX, and MW acquired funding. QL, XLH, MW, HY, and WLM performed data and logical analysis and organization. QL, SJC, WLM, and HY wrote the original draft of the manuscript. XLH, LX, MW, WLM, HY, and QL wrote and reviewed the manuscript.

## Funding support

- National Natural Science Foundation of China (82270111 and 81973991 to WLM; 82070066 and 82270075 to HY; 82070098 to LX; 82200081 to MW).

## Supplementary Material

Supplemental data

Unedited blot and gel images

Supporting data values

## Version 1 10/16/2025

In-Press Preview

## Version 2 12/15/2025

Electronic publication
