# Supplementary material for: Excessive collagen type VII mediates pleural fibrosis via increasing extracellular matrix stiffness
Source: J Clin Invest. 2025 Oct 16;135(24):e188822. doi: 10.1172/JCI188822 (PMC12700545; doi:10.1172/JCI188822)
Supplement: Supplemental data [file jci-135-188822-s060.pdf]

Online Supplementary Materials

Title: Excessive collagen type VII mediates pleural fibrosis via increasing extracellular matrix stiffness

Authors: Qian Li *et al*

Contents:

1. **Online Tables:**

Table S1. Demographics of patients recruited in the study

Table S2. Sequences of siRNA

Table S3. Primers used for chip-qPCR

Table S4. Primers used for RT-qPCR

2. **Online Figures:**

Figure S1. Expression of collagen VII and collagen I in human pleural tissues

Figure S2. Dynamic expressions of collagen I and α-SMA during fibrotic process in mouse pleural fibrosis model

Figure S3. Expression of collagen VII in pleural mesothelial cells, lung fibroblasts, bronchial epithelial cells and alveolar epithelial cells

Figure S4. Effect of bleomycin (BLM), tuberculous pleural effusion (TBPE) and TGF-β1 on expression of collagen VII in pleural mesothelial cells (PMCs)

Figure S5. Inhibition of collagen VII by siRNA prevented fibrosis-related protein expressions induced by bleomycin (BLM), tuberculous pleural effusion (TBPE) in PMCs

Figure S6. Recombinant collagen VII protein promoted collagen I and α-SMA expression in rat PMCs

Figure S7. Collagen VII mediated collagen I and α-SMA expression in primary human pleural mesothelial cells (HPMCs)

Figure S8. Mesothelial cell-specific deletion of collagen VII attenuated pleural fibrosis induced by bleomycin (BLM) plus carbon particles

Figure S9. Immunofluorescence and Sirius Red staining of mouse pleural tissues

Figure S10. Mesothelial cell-specific deletion of collagen VII attenuated pleural fibrosis induced by Bacillus Calmette-Guérin (BCG) or tuberculous pleural effusion (TBPE)

Figure S11. Stiff PDMS gels induced collagen I and α-SMA expression in primary rat pleural mesothelial cells (PMCs)

Figure S12. ITGAV siRNA and cilengitide prevented increases of collagen I and α-SMA in vitro

Figure S13. Tuberculous pleural effusion (TBPE) and bleomycin (BLM) activated PI3K-AKT pathway in cultured-PMCs

Figure S14. Collagen VII-induced ECM stiffness activated integrin/PI3K-AKT signaling

Figure S15. Increased ECM stiffness activated integrin/PI3K-AKT signaling

Figure S16. JUN is a transcription factor for type I collagen

Figure S17. *Jun* siRNA prevented up-expression of collagen I and α-SMA induced by recombinant collagen VII protein and stiff matrix

Figure S18. JUN activated transcription of the gene of collagen VII

Figure S19. Second Harmonic Generation (SHG) validation of human pleural tissue samples

Figure S20. Identification of primary human pleural mesothelial cells (HPMCs)

1. Online Tables

Table S1. Demographics of patients recruited in the study

Subject	Age	Sex	Clinical diagnosis
Control 1	29	F	Lung adenocarcinoma * #
Control 2	54	M	Lung adenocarcinoma * #
Control 3	54	M	Lung adenocarcinoma * #
Control 4	65	M	Lung adenocarcinoma * #
Control 5	51	M	Lung adenocarcinoma *
Patient 1	57	M	Tuberculous pleurisy #
Patient 2	71	M	Tuberculous pleurisy #
Patient 3	68	M	Tuberculous pleurisy #
Patient 4	33	F	Tuberculous pleurisy #
Patient 5	59	M	Tuberculous pleurisy
Patient 6	51	M	Tuberculous pleurisy
Patient 7	53	M	Tuberculous pleurisy
Patient 8	59	M	Tuberculous pleurisy
Patient 9	80	M	Tuberculous pleurisy
Patient 10	54	M	Tuberculous pleurisy
Patient 11	76	M	Tuberculous pleurisy
Patient 12	65	M	Tuberculous pleurisy
Patient 13	45	M	Tuberculous pleurisy
Patient 14	63	M	Tuberculous pleurisy
Patient 15	28	M	Tuberculous pleurisy
Patient 16	35	M	Tuberculous pleurisy
Patient 17	52	M	Tuberculous pleurisy
Patient 18	50	M	Tuberculous pleurisy
Patient 19	35	M	Tuberculous pleurisy
Patient 20	32	M	Tuberculous pleurisy
Patient 21	63	M	Tuberculous pleurisy

*Normal pleural tissue resected from adenocarcinoma

Patients recruited in pleural tissue proteomic analysis

Table S2. Sequences of siRNA

Gene	Sequences (5'-3')
Col7a1siRNA(rat)-1	CAAAGACUCUGCAGAGAUAAC
	UAUCUCUGCAGAGUCUUUGUU
Col7a1siRNA(rat)-2	CGGUGUGUCUGGACCUGAAGG
	UUCAGGUCCAGACACACCGCG
Col7a1siRNA(rat)-3	CGAGUACCGUUUCACUGUACG
	UACAGUGAAACGGUACUCGGU
Col7a1siRNA(human)-1	GGGGCAGGGGGUCAAGCUAUU
	UAGCUUGACCCCCUGCCCCUU
Col7a1siRNA(human)-2	GGGCAGCAAUGGUGACCAAGG
	UUGGUCACCAUUGCUGCCCGG
Col7a1siRNA(human)-3	GAAUGGUGCUGCAGGCAAAGC
	UUUGCCUGCAGCACCAUUCGG
Itgav(rat)siRNA-1802	GCUUAAAGGCGGAUGGCAATT
	UUGCCAUCCGCCUUUAAGCTT
Itgav(rat)siRNA-767	GCACAAAGACCGUUGAGUATT
	UACUCAACGGUCUUUGUGCTT
Itgav(rat)siRNA-1106	CAGCAAGGACUUUGGGAAUTT
	AUUCCCAAAGUCCUUGCUGTT
Itgav(rat)siRNA-1802	GCUUAAAGGCGGAUGGCAATT
	UUGCCAUCCGCCUUUAAGCTT
Itgav(rat)siRNA-767	GCACAAAGACCGUUGAGUATT
	UACUCAACGGUCUUUGUGCTT
Itgav(rat)siRNA-1106	CAGCAAGGACUUUGGGAAUTT
	AUUCCCAAAGUCCUUGCUGTT
JUN(rat)siRNA- 1732	GGAUCAAGGCGGAGAGGAATT
	UUCCUCUCCGCCUUGAUCCTT
JUN(rat)siRNA-1276	CCAAGAACGUGACAGAUGATT
	UCAUCUGUCACGUUCUUGGTT
JUN(rat)siRNA-1489	CAAACCUCAGCAACUUCAATT

Table S3. Primers used for chip-qPCR

Site		Sequences
COL1A1-siteA	Forward	GCAGGACTTTGGTGGGTTC
COL1A1-siteA	Reverse	ACAGCAATGGAGGGATGGA
COL1A1-siteB	Forward	GATGATTCTCTCTGGTTCC
COL1A1-siteB	Reverse	TGATTGACTTTGTTTTAGG
COL1A1-siteC	Forward	ATCCCATACATCCCCAACA
COL1A1-siteC	Reverse	ATAAAGCCCCTTCTCCAGT
COL7A1-siteA	Forward	ATGGCCTGAGGGCTTGTCT
COL7A1-siteA	Reverse	GCAGCGCCGTGGGGGGTGG
COL7A1-siteB	Forward	CAGGGACCTACAAGAAAAG
COL7A1-siteB	Reverse	GACCACCCACAGTGACAGC
COL7A1-siteC	Forward	CCTTGGTTTCCCTAAGACC
COL7A1-siteC	Reverse	GCCACTCCTGTGATTCAGC

Table S4. Primers used for qRT-PCR

Gene		Human Sequences	Rat Sequences
COL7A1	Forward	TGAGGAGCAGAAGAAGGCAAG	TGCTGTGGATAATGGCTTAGACTTGG
	Reverse	CCTGGCTGATAGATGCCAAGA	CAAGGCTCTAGTTGTGGCTGTATGG
COL1A1	Forward	TAGGGTCTAGACATGTTCAGCTTTGT	CACTGTCCTTGTCGATGGCT
	Reverse	GTGATTGGTGGTGGGATGTCT	GGCAGGCGAGATGGCTTATT
α-SMA	Forward	GAGCGTGGCTATTCCTTCGT	AGCATCCGACCTTGCTAACG
	Reverse	GCCCATCAGGCAACTCGTAA	AGAGTCCAGCACAATACCAGTTG
GAPDH	Forward	TGGCTACAGCAACAGGGTGG	TGCCACTCAGAAGACTGTGG
	Reverse	GGTACATGACAAGGTGCGGCT	TTCAGCTCTGGGATGACCTT

2. Online Figures

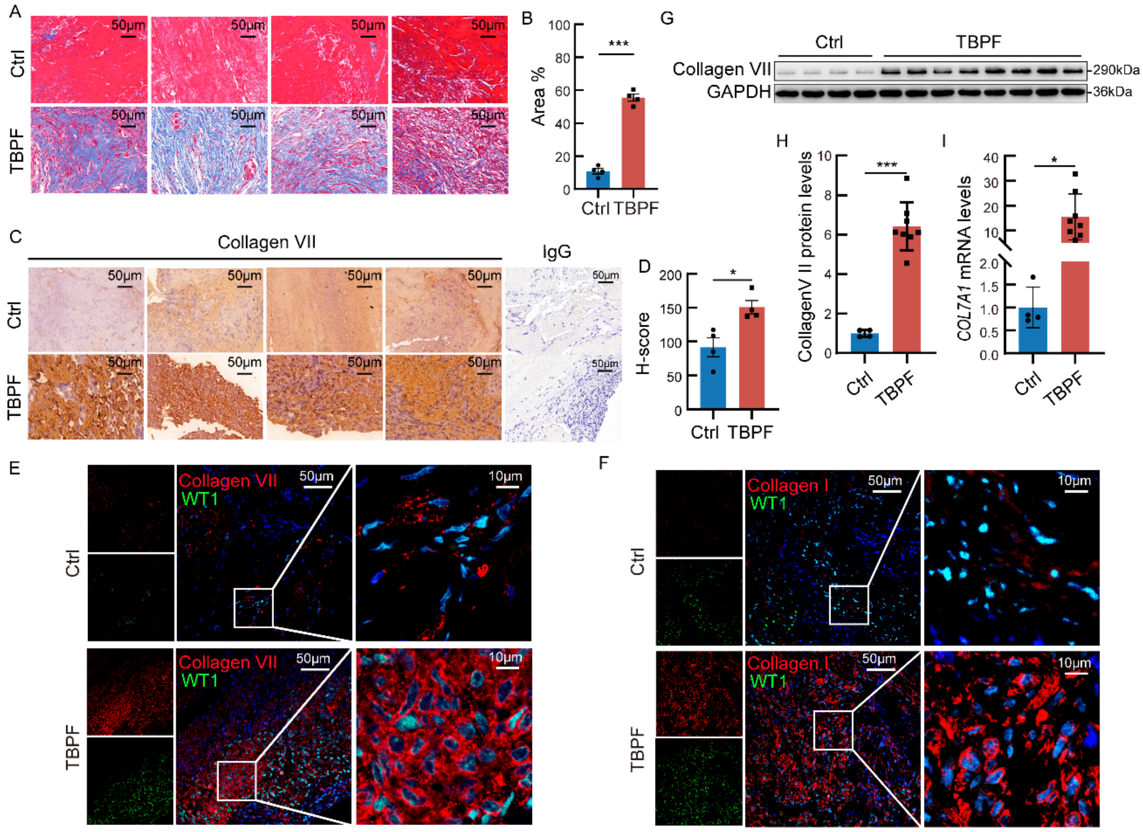

Figure S1. Expressions of collagen VII and collagen I in human pleural tissues. (A) Representative images of Masson's trichrome staining of pleural tissues. Color blue is showing collagen fibers. Scale bar, 50 μm. (B) Changes in collagen area percentages in the pleural tissues according to A. n=4. (C) Representative images of immunohistochemistry staining of collagen VII in pleural tissues. Color brown is showing positive staining. Scale bar, 50 μm. (D) H-score quantification of immunohistochemical staining for collagen VII according to C. n=4. (E) Immunofluorescence staining of collagen VII (red) and WT-1 (green, WT-1 expresses in the nucleus). (F) Immunofluorescence staining of collagen I (red) and WT-1 (green). Nuclei were counterstained with DAPI (blue). Scale bar, 50 μm or 10 μm. TBPF: human tissues from patients with tuberculous pleural fibrosis. (G-I) Western blotting and RT-qPCR analysis of collagen VII expression in human pleural tissues. Ctrl, n=4; TBPF, n=8. Results were expressed as mean ± SD. Statistical significance was determined by unpaired two-tailed Student's t-tests. $*P < 0.05$, $***P < 0.001$. Ctrl: control pleural tissues. Pleural tissues in TBPF and Ctrl groups were obtained as described as the Methods.

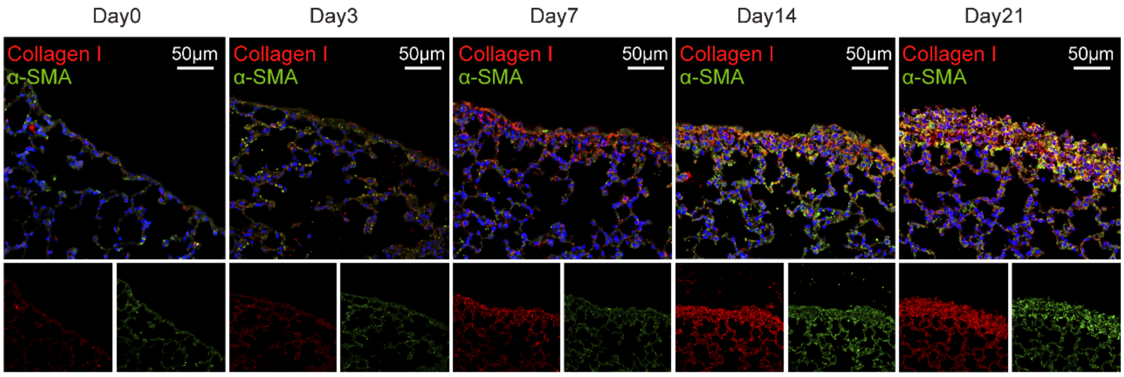

Figure S2. Dynamic expressions of collagen I and α-SMA during fibrotic process in mouse pleural fibrosis model. Mouse pleural fibrosis models were made as described as the Methods. Immunofluorescence staining of collagen I (red) and α-SMA (green) was performed. Nuclei were counterstained with DAPI (blue). Scale bar, 50 μm.

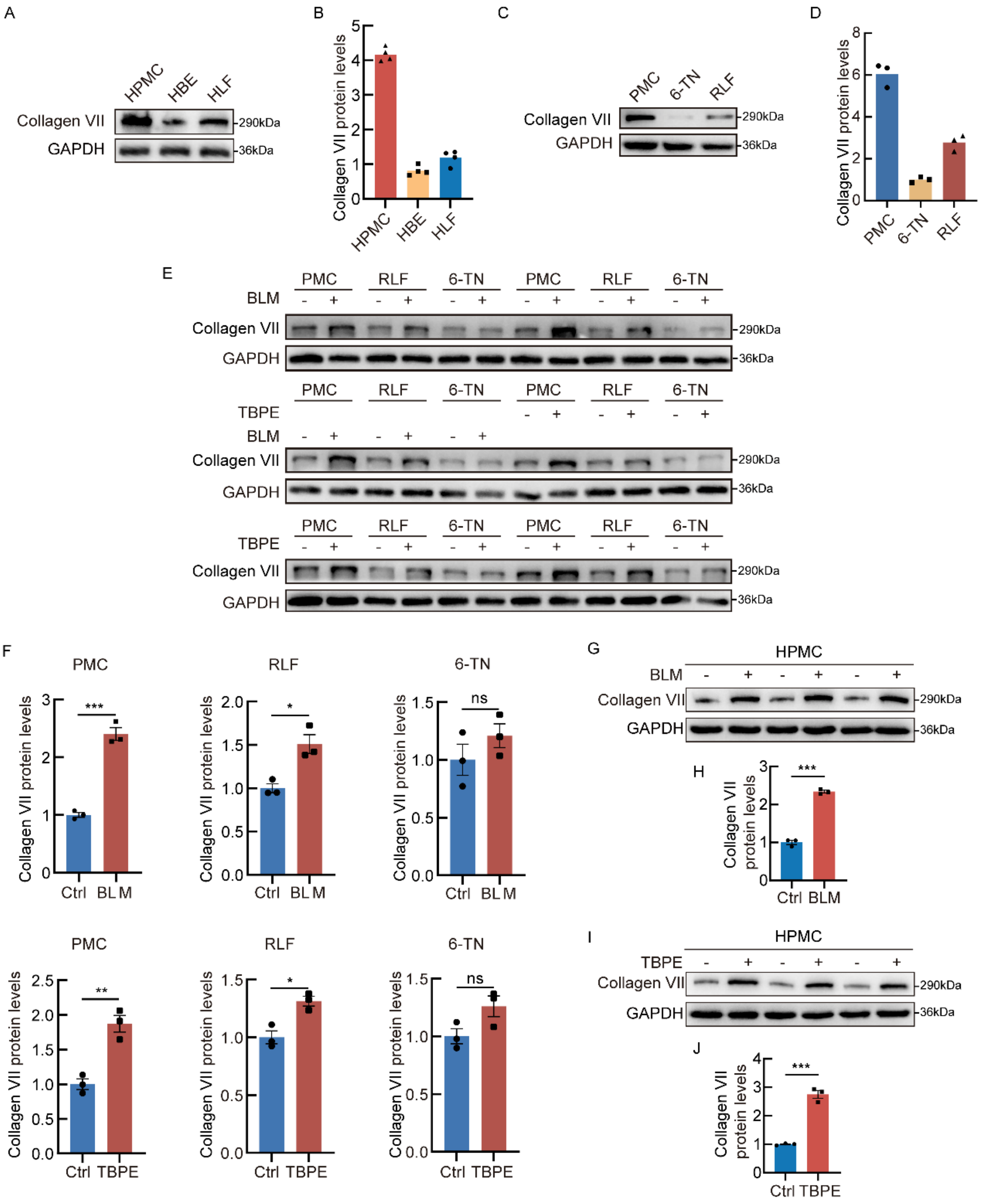

Figure S3. Expression of collagen VII in pleural mesothelial cells, lung fibroblasts, bronchial epithelial cells and alveolar epithelial cells. (A-D) Expression of collagen VII in different types of cells. HPMC, primary human pleural mesothelial cell. HBE: human bronchial epithelial cell line cell. HLF, primary human lung fibroblast. PMC, primary rat pleural mesothelial cell. 6-TN (RLE 6-TN), rat type II alveolar epithelial cell line cell. RLF, primary rat lung fibroblast. n=4 (B). n=3 (D). (E-F) Rat cells were treated with bleomycin (BLM, 0.2

μg/ml) or tuberculous pleural effusion (TBPE, 5%) for 24 h, after which collagen VII was detected by western blotting. Results were expressed as mean ± SD. Statistical significance was determined by unpaired two-tailed Student's t-tests. n=3. $^*P < 0.05$, $^{**}P < 0.01$, $^{***}P < 0.001$. ns: not significant. (G-J) Primary human cells, HPMCs were treated with bleomycin (BLM, 0.2 μg/ml) or tuberculous pleural effusion (TBPE, 5%) for 24 h, after which collagen VII was detected by western blotting. Results were expressed as mean ± SD. Statistical significance was determined by unpaired two-tailed Student's t-tests. n=3. $^{***}P < 0.001$.

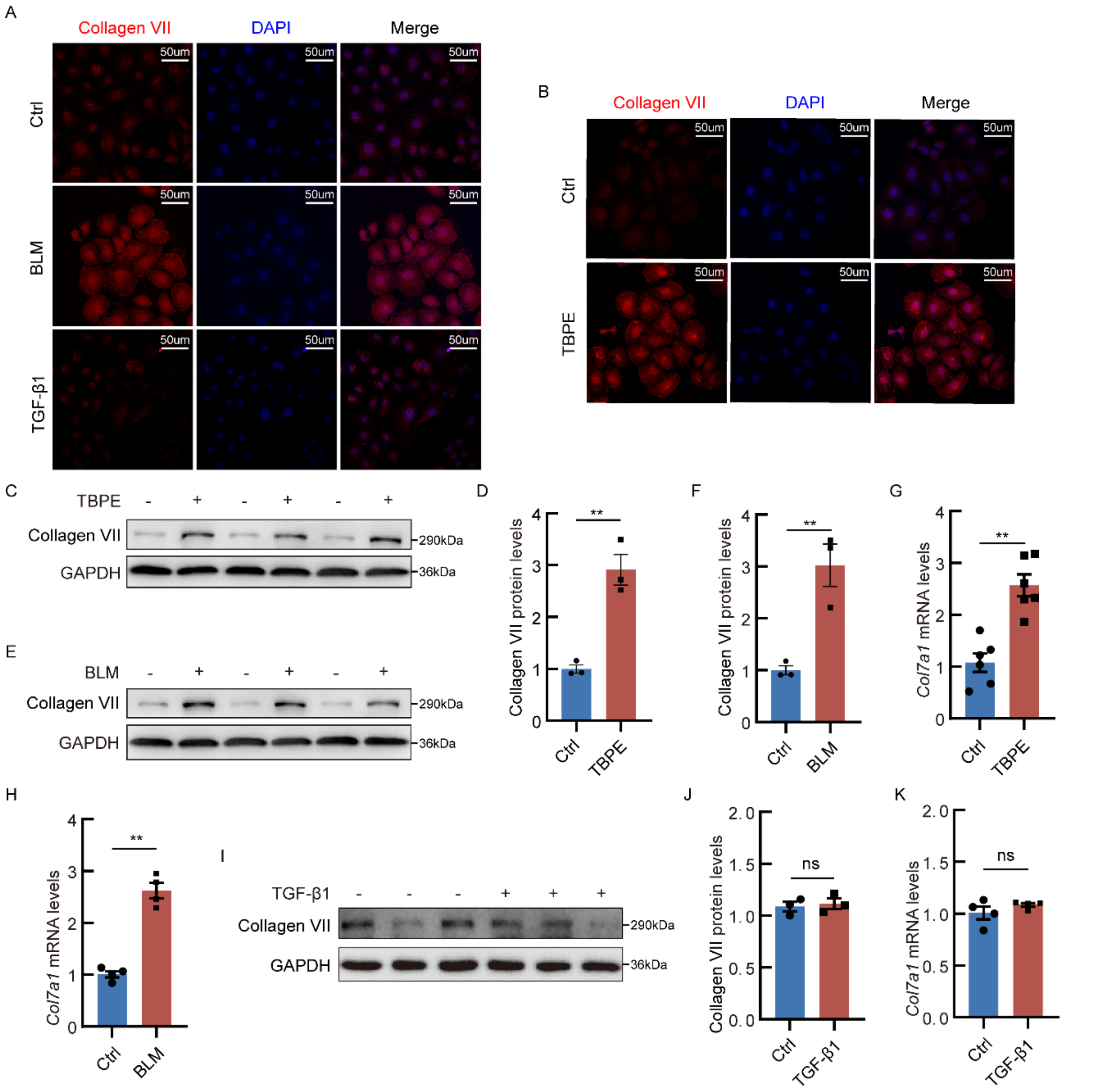

Figure S4. Effect of bleomycin (BLM), tuberculous pleural effusion (TBPE) and TGF-β1 on expression of collagen VII in pleural mesothelial cells (PMCs). PMCs were treated with bleomycin (BLM, 0.2 μg/ml), TGF-β1 (10 ng/ml), transudative pleural effusion (TPE, 5%), or tuberculous pleural effusion (TBPE, 5%) for 24 h, then indicated experiment was performed. (A, B) Immunofluorescence staining of collagen VII (red). (C-K) PMCs were harvested for western blotting and RT-qPCR. (C, E, I) Images of western blots. (D, F, J) Bar graphs showing changes of proteins according to C, E, I. n=3. (G, H, K) Bar graphs showing changes of *Col7a1* mRNA. n=6 (G). n=4 (H, K). Results were expressed as mean±SD. Statistical significance was determined by unpaired two-tailed Student's t-tests. **$P < 0.01$. ns: not significant.

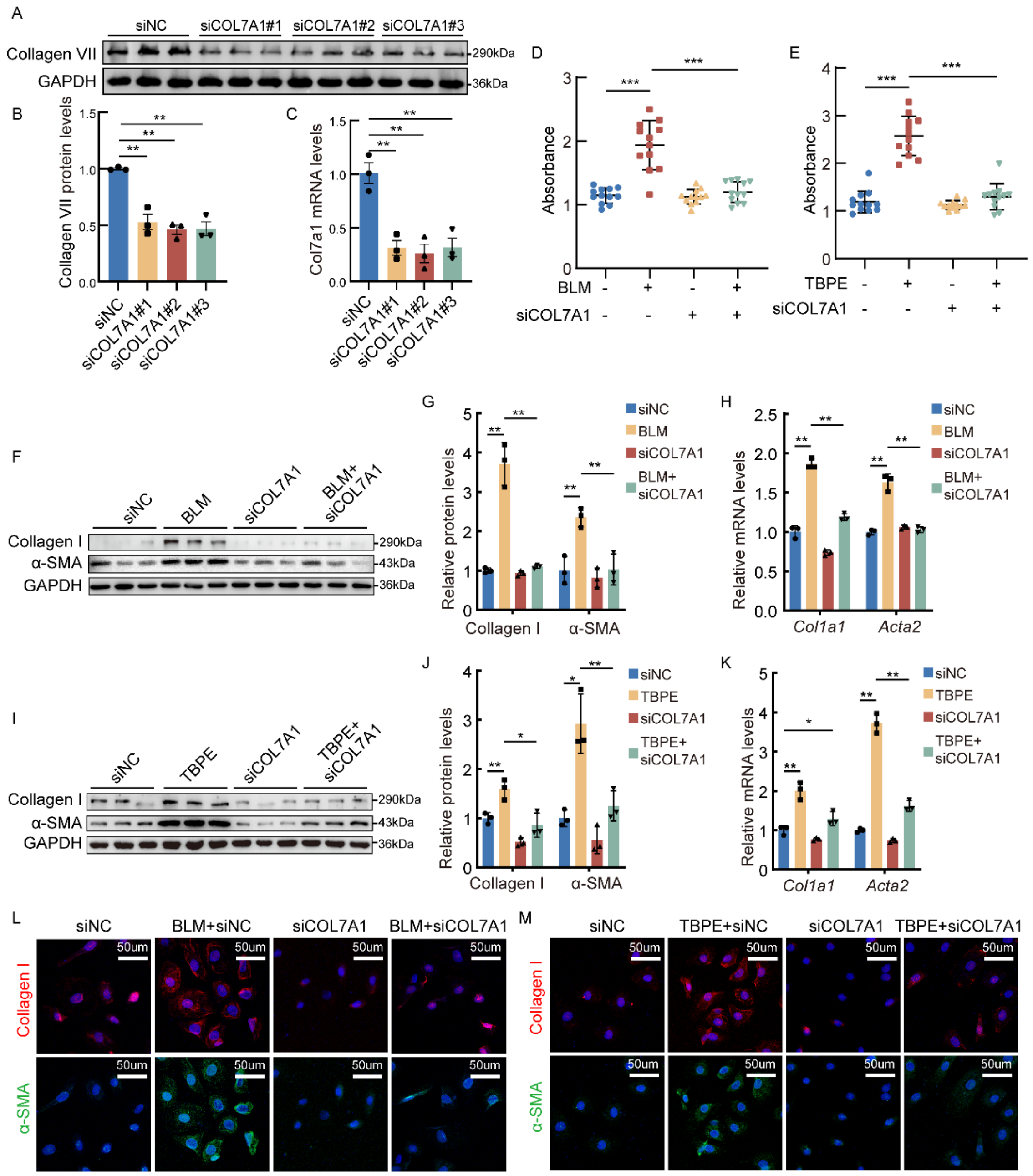

Figure S5. Inhibition of collagen VII by siRNA prevented fibrosis-related protein expressions induced by bleomycin (BLM), tuberculous pleural effusion (TBPE) in PMCs. (A-C) Western blotting and qRT-PCR were used to detect knockdown efficiency of three siRNAs targeting *Col7a1*. Data were expressed as mean±SD. Statistical significance was determined using one-way ANOVAs. n=3. $^{**}P<0.01$. (D-M) After transfected with control siRNA or *Col7a1* siRNA for 36 h, PMCs were cultured with or without BLM (0.2 μg/ml),

tuberculous pleural effusion (TBPE, 5%) for 24 h, then indicated experiment was performed. (D, E) Cell proliferation was detected by CCK8 analysis. Data were expressed as mean±SD. Statistical significance was determined using one-way ANOVA. n=12. ***P<0.001. (F-K) PMCs were harvested for western blotting and qRT-PCR to detect protein and mRNA expressions of collagen I and α-SMA. (F, I) Images of western blots. (G, J) Bar graphs showing changes of proteins according to F, I. (H, K) Bar graphs showing changes of *Col1a1* and *Acta2* mRNA. Data were expressed as mean±SD. Statistical significance was determined using one-way ANOVA. n=3. *P<0.05, **P<0.01. (L, M) Immunofluorescence staining of collagen I (red) and α-SMA (green) in PMCs. siNC: control siRNA. siCOL7A1: *Col7a1* siRNA.

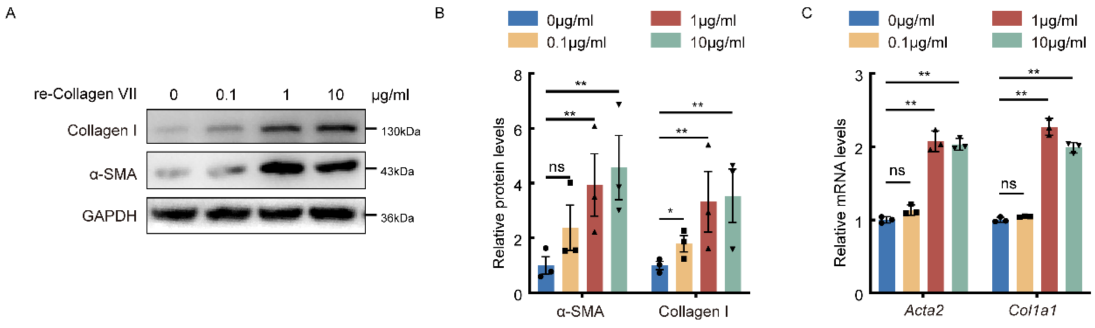

Figure S6. Recombinant collagen VII protein promoted collagen I and α-SMA expression in rat PMCs. Primary rat PMCs were cultured with series concentrations of recombinant collagen VII protein (0, 0.1, 1, 10 μg/ml) in the medium for 24 h, after which collagen I and α-SMA protein and mRNA expression was investigated by western blotting and RT-qPCR. (A) Representative images of western blots. (B) Bar graphs showing changes of proteins according to A. (C) Bar graphs showing changes of *Col1a1* and *Acta2* mRNA. Data were expressed as mean±SD. Statistical significance was determined using one-way ANOVA. n=3. *P<0.05, **P<0.01. ns: not significant.

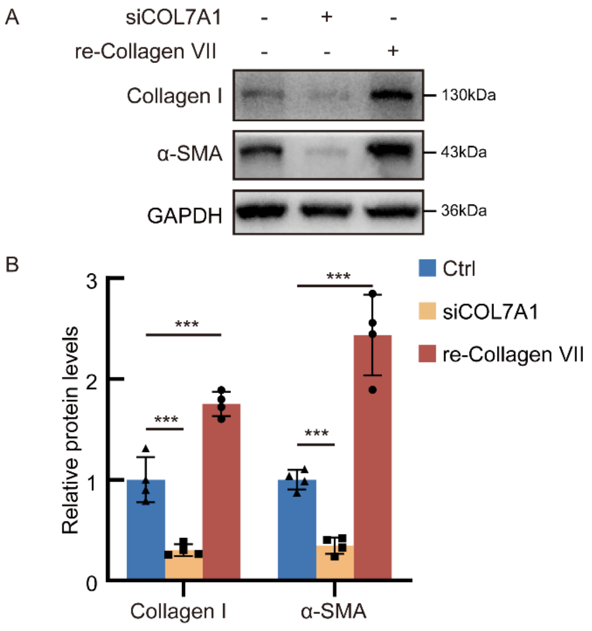

Figure S7. Collagen VII mediated collagen I and α-SMA expression in primary human pleural mesothelial cells (HPMCs). Primary HPMCs were isolated and cultured as described as the Methods. After transfected with *Col7a1* siRNA for 36 h, primary HPMCs were normally cultured for another 24 h. On the other hand, recombinant collagen VII (1 μg/ml) was used to treat primary HPMCs for 24 h. Then cells were harvested, after which collagen I and α-SMA proteins were detected by western blotting. (A) Representative images of blots. (B) Bar graphs were showing changes of protein according to A. Data were expressed as mean ± SD. Statistical significance was determined by one-way ANOVA. n = 4. ***$P < 0.001$. siCOL7A1: *Col7a1* siRNA. re-Collagen VII: recombinant collagen VII protein.

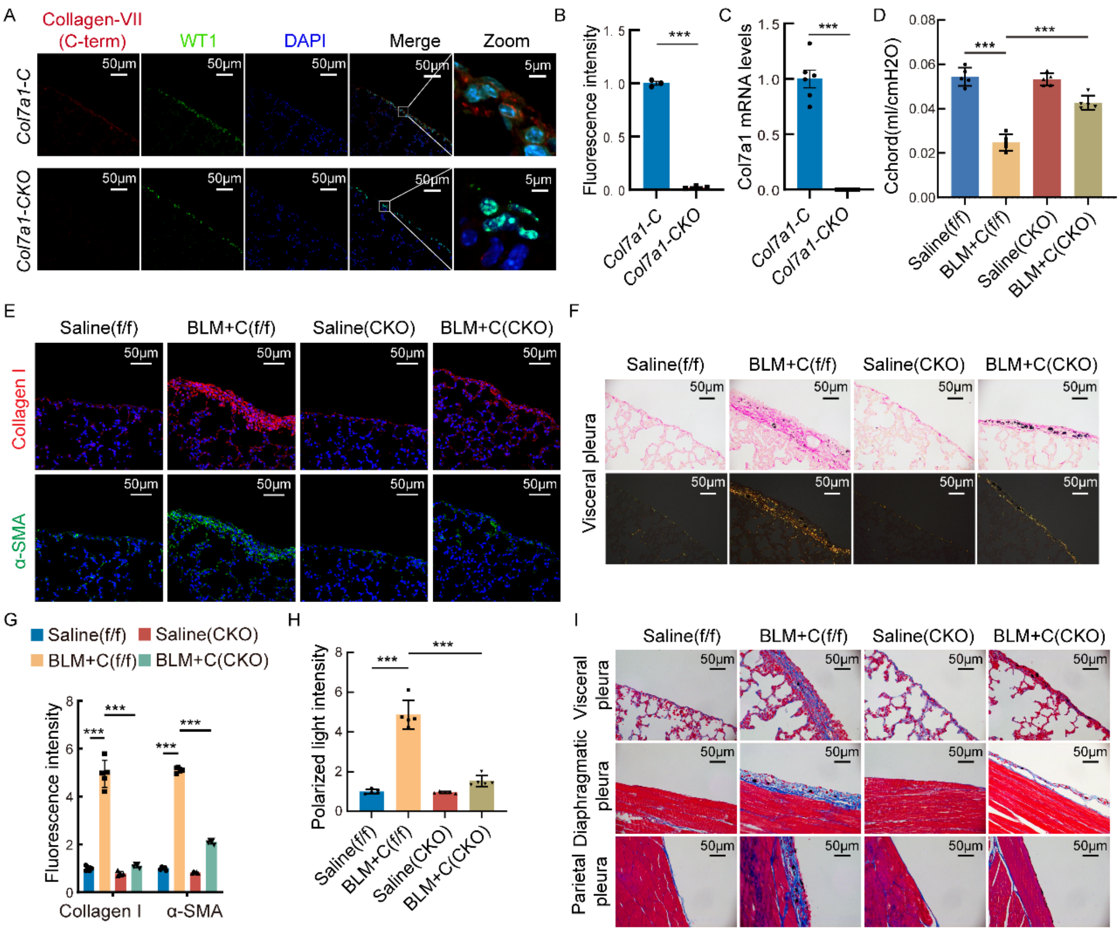

Figure S8. Mesothelial cell-specific deletion of collagen VII attenuated pleural fibrosis induced by bleomycin (BLM) plus carbon particles. (A-C) *Wt1-Cre*$^{+}$*Col7a1*$^{flox/flox}$ mice were made as described as the Methods. Efficiency of *Col7a1* knockout was detected. (A) Tissue sections were stained with anti-C-terminal collagen VII antibody (red) and WT-1 (green), and nuclei were counterstained with DAPI (blue). Scale bar, 50 μm. (B) Bar graph showing changes of fluorescence intensity of red color to reveal collagen VII according to A. n=3. (C) RT-qPCR analysis of *Col7a1* in pleural tissues of *Wt1-Cre*$^{+}$*Col7a1*$^{flox/flox}$ and *Wt1-Cre*$^{-}$*Col7a1*$^{flox/flox}$ mice. n=6. (B, C) Results were expressed as mean ± SD. Statistical significance was determined by unpaired two-tailed Student's t-tests. ***P < 0.001. (D-I) Pleural fibrosis models were made by intrapleural injection of BLM plus carbon particles in control and *Wt1-Cre*$^{+}$*Col7a1*$^{flox/flox}$ mice. Before lung and pleural tissues were taken at days 21 post-injection,

lung function tests were performed. (D) Lung function test results. Cchord: chord compliance. Data were presented as mean±SD. Statistical significance was determined using one-way ANOVA. n=5. *** $P < 0.001$. (E) Representative images of immunofluorescence staining for collagen I and α-SMA in visceral pleura. Scale bar, 50 μm. (F) Bar graphs showing changes of fluorescence intensity corresponding to E. Data were presented as mean±SD. Statistical significance was analyzed using one-way ANOVA. n=5. ***$P < 0.001$. (G) Representative images of sirius red staining of visceral pleura under polarized light microscopy. Scale bar, 50 μm. (H) Bar graphs showing changes of polarized light intensity corresponding to G. Data were presented as mean±SD. Statistical significance was analyzed using one-way ANOVA. n=5. ***$P < 0.001$. (I) Representative images of Masson's trichrome staining of visceral, parietal, and diaphragmatic pleura. Scale bar, 50 μm. Scale bar, 50 μm. BLM+C: BLM plus carbon particles. CKO: conditional knockout.

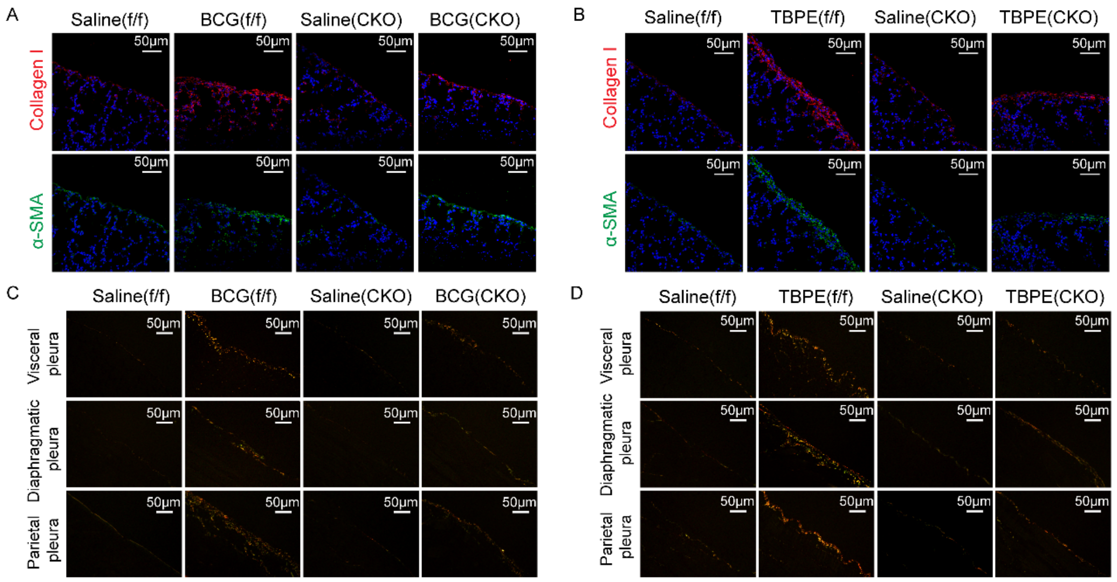

Figure S9. Immunofluorescence and Sirius Red staining of mouse pleural tissues. Pleural fibrosis models were made by intrapleural injection of Bacillus Calmette-Guérin (BCG) plus carbon or tuberculous pleural effusion (TBPE) in control and *Wt1-Cre*$^{+}$*Col7a1*$^{flox/flox}$ mice as the Figure 3. (A, B) Immunofluorescence staining for collagen I (red) and α-SMA (green) in visceral pleura. Scale bar, 50 μm. (C, D) Representative sirius red staining of visceral, parietal, and diaphragmatic pleura under polarized light microscopy. TBPE: tuberculous pleural effusion. CKO: conditional knockout.

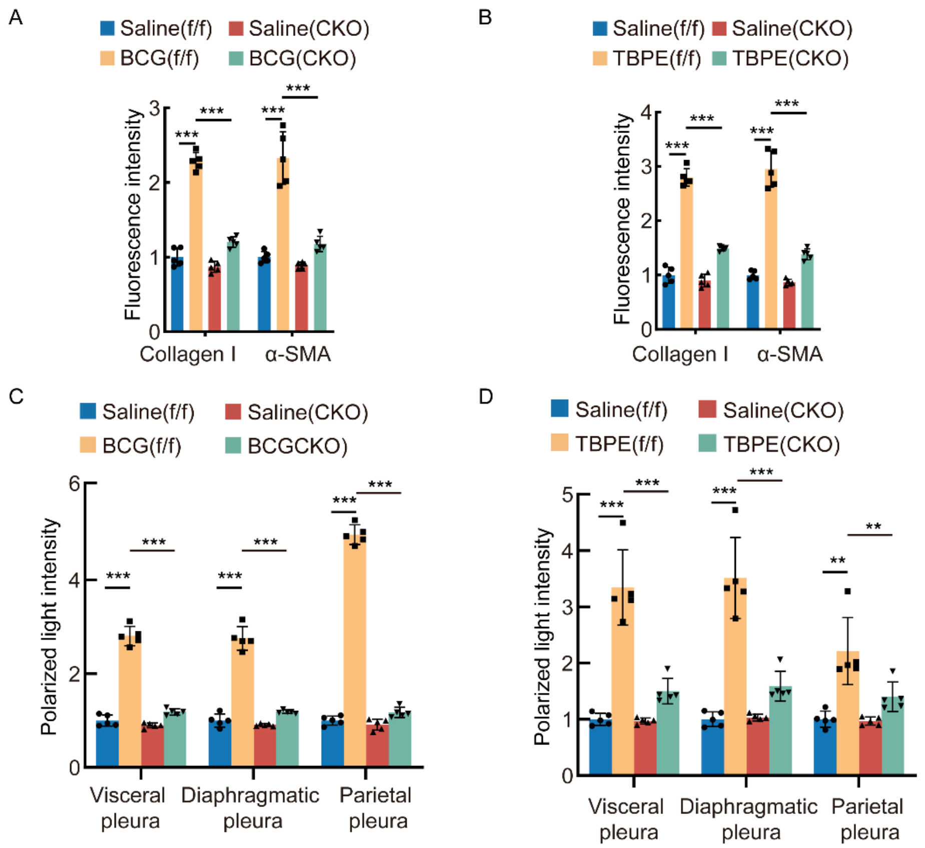

Figure S10. Mesothelial cell-specific deletion of collagen VII attenuated pleural fibrosis induced by Bacillus Calmette-Guérin (BCG) or tuberculous pleural effusion (TBPE). Pleural fibrosis models were made by intrapleural injection of BCG plus carbon or tuberculous pleural effusion in control and *Wt1-Cre*$^{+}$*Col7a1*$^{flox/flox}$ mice as the Figure 3. Immunofluorescence staining for collagen I and α-SMA in visceral pleura, and sirius red staining of visceral, parietal, and diaphragmatic pleura were performed as the Figure S9. Statistical analysis was made to reveal changes of proteins or polarized light intensity. Bar graph (A) according to Figure S9A, (B) according to Figure S9B, (C) according to Figure S9C, (D) according to Figure S9D. Data were presented as mean±SD. Statistical significance was analyzed using one-way ANOVA. n = 5. ** $P < 0.01$. n = 5. *** $P < 0.001$. CKO: conditional knockout.

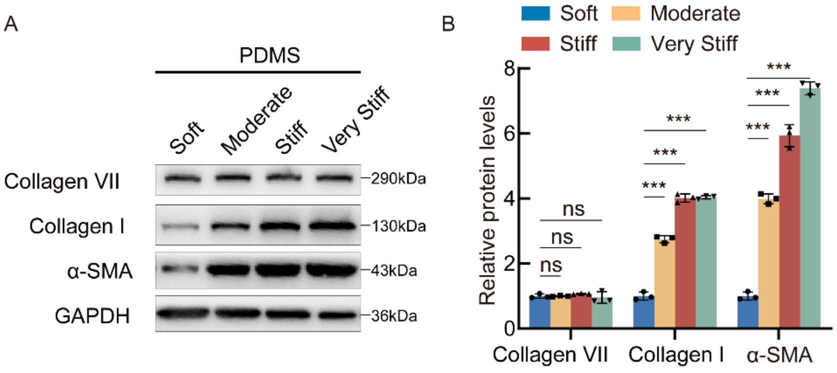

Figure S11. Stiff PDMS gels induced collagen I and α-SMA expression in primary rat pleural mesothelial cells (PMCs). Preparation of PDMS gels was described in the Methods. PDMS was mixed at base-to-curing-agent ratios of 60:1, 40:1, 20:1, and 10:1 (by weight) corresponding to control, soft, moderate, and stiff substrates. PDMS gels with different stiffness were used to culture primary rat PMCs for 24 h, after which cells were harvested for western blotting. (A) Representative images of Western blots. (B) Bar graph showing changes of proteins according to density of blots. Data were presented as mean ± SD. Statistical significance was assessed using one-way ANOVA. n=3. ***$P < 0.001$. ns: not significant.

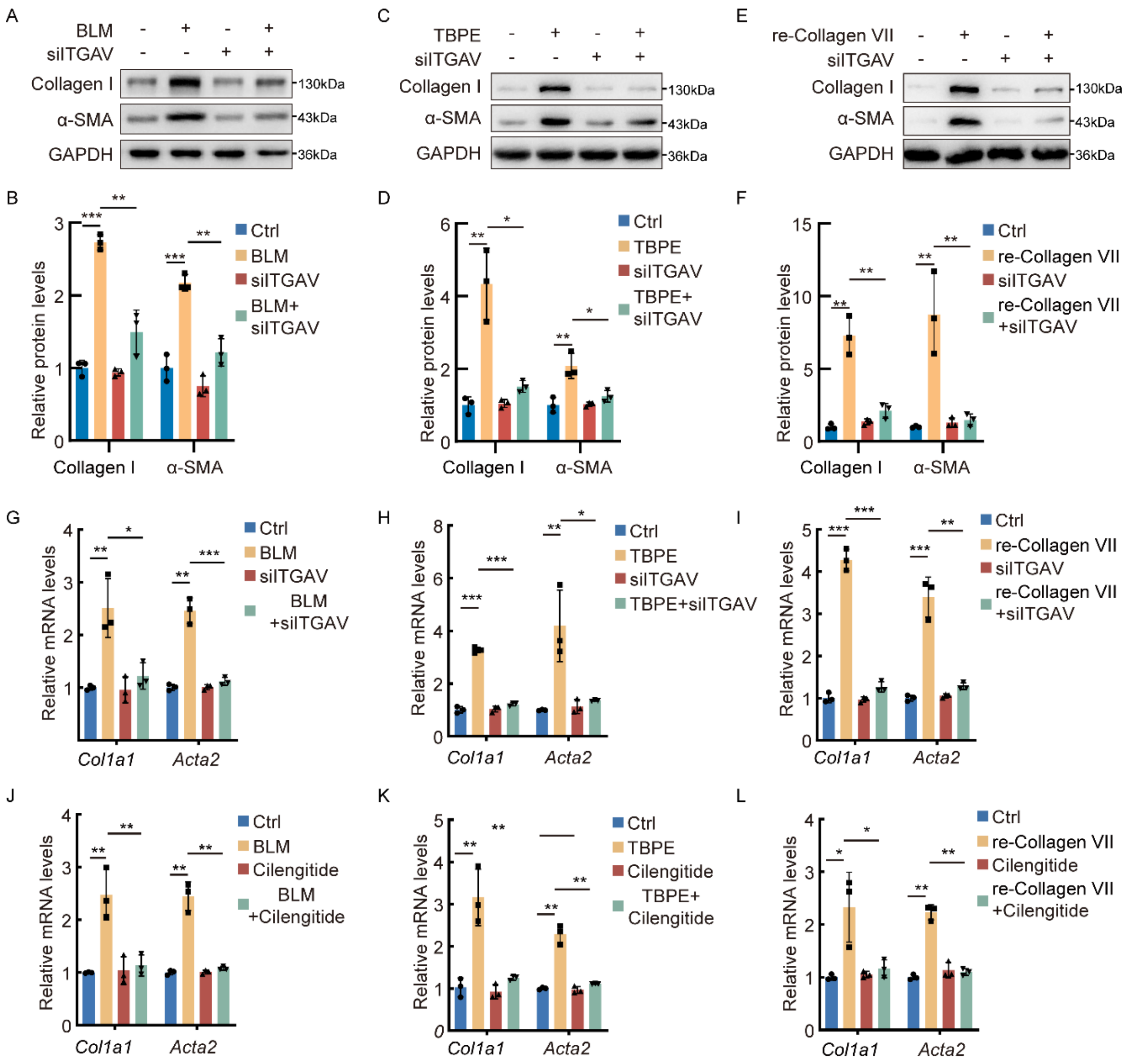

Figure S12. ITGAV siRNA and cilengitide prevented increases of collagen I and α-SMA *in vitro*. (A-I) PMCs were prepared by transfecting *Itgav* siRNA or control siRNA for 36 h. Primary rat PMCs were treated by BLM (0.2 μg/ml), tuberculous pleural effusion (TBPE, 5%), and recombinant collagen VII (1 μg/ml) with or without *Itgav* siRNA for 24 h, after which collagen I and α-SMA were detected by western blotting and RT-qPCR. (A, C, E) Representative images of western blots of collagen I and α-SMA protein. (B, D, F) The bar graphs showing changes of protein according to A, C, E, respectively. (G-I) The levels of *Col1a1* and *Acta2* mRNAs detected by RT-qPCR. Data were expressed as mean±SD. Statistical significance was determined using one-way ANOVA. n=3. *$P < 0.05$, **$P < 0.01$, ***$P < 0.001$. (J-L) PMCs were treated by BLM, TBPE, and recombinant collagen VII with or without cilengitide (10 μg/ml) for 24 h, after which *Col1a1* and *Acta2* mRNAs were detected by RT-qPCR. Data were expressed as mean±SD. Statistical significance was determined using one-way ANOVA. n=3. *$P < 0.05$, **$P < 0.01$, ***$P < 0.001$. siITGAV: *Itgav* siRNA. re-Collagen VII: recombinant collagen VII protein.

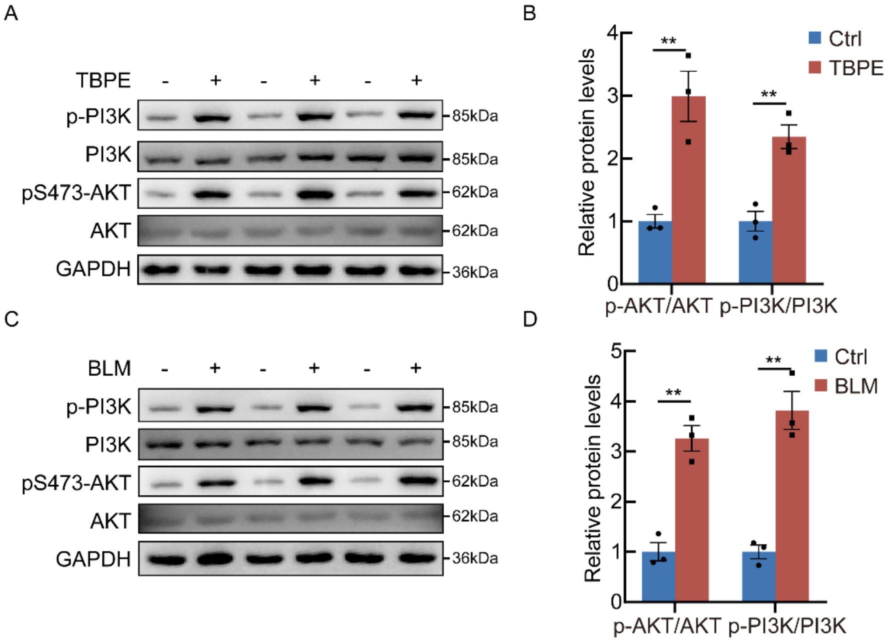

Figure S13. Tuberculous pleural effusion (TBPE) and bleomycin (BLM) activated PI3K-AKT pathway in cultured-PMCs. Cultured PMCs were treated with TBPE (5%) or BLM (0.2 μg/ml) for 24 h, after which p-PI3K, PI3K, pS473-AKT, and AKT proteins were investigated by western blotting. (A, C) Representative images of western blots. (B, D) Bar graphs showing changes of proteins according to A, C. Data were expressed as mean±SD. Statistical significance was determined using unpaired two-tailed Student's t-tests. n=3. $^{**}P < 0.01$.

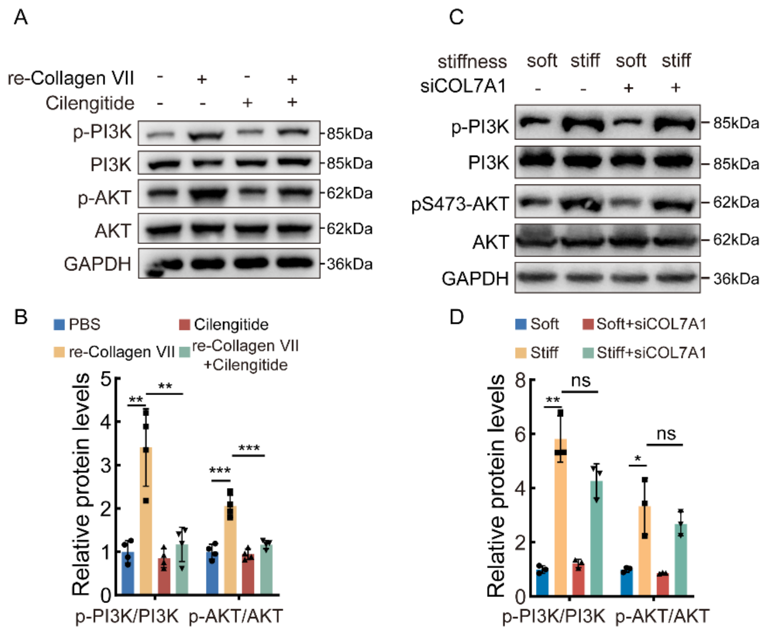

Figure S14. Collagen VII-induced ECM stiffness activated integrin/PI3K-AKT signaling. (A, B) Effects of recombinant collagen VII on PI3K/AKT signalings. Cultured PMCs were treated with recombinant collagen VII protein with or without cilengitide (10 μg/ml) for 24 h, then harvested for western blotting (A). (B) Bar graphs showing changes of proteins according to A. Data were expressed as mean±SD. Statistical significance was determined by one-way ANOVA. n=4. $**P < 0.01$, $***P < 0.001$. (C, D) Effect of *Col7a1* siRNA on PI3K/AKT signals induced by stiff substrate. After transfected control siRNA or *Col7a1* siRNA for 36 h, PMCs were cultured in soft or stiff substrate medium for 24 h, then harvested for western blotting (C). (D) Bar graphs showing changes of proteins according to C. Data were expressed as mean± SD. Statistical significance was determined by one-way ANOVA. n = 4. $***P < 0.001$. ns: not significant. siCOL7A1: *Col7a1* siRNA. re-Collagen VII: recombinant collagen VII protein.

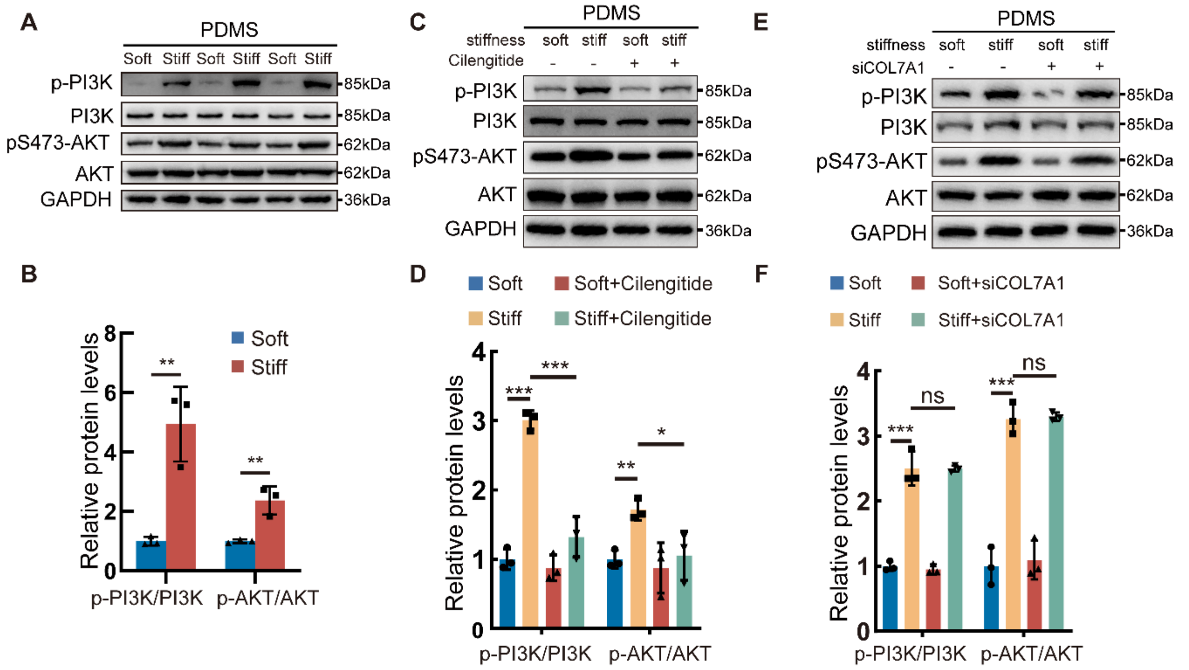

Figure S15. Increased ECM stiffness activated integrin/PI3K-AKT signaling. Preparation procedure of PDMS gels was described in the Methods. Soft and stiff PDMS gels were made using PDMS at base-to-curing-agent ratios of 40:1 and 10:1 (by weight), respectively. Primary rat PMCs were cultured and treated by indicated treatments for 24 h, after which cells were harvested for western blotting. (A, B) Effects of stiff PDMS gels on activation of PI3K/AKT pathway. (C, D) Effects of integrin inhibitor (cilengetide) on activation of PI3K/AKT pathway. (E, F) Effects of *Col7a1* siRNA on activation of PI3K/AKT pathway. (A, C, E) Representative images of Western blots. (B, D, F) Bar graph showing changes of proteins according to density of blots in A, C, E, respectively. Data were expressed as mean ± SD. Statistical significance was determined using unpaired two-tailed Student's t-tests in B. n=3. ***P* < 0.01. Statistical significance was assessed using one-way ANOVA in D and F. n=3. **P* < 0.05, ***P* < 0.01, ****P* < 0.001. ns: not significant. siCOL7A1: *Col7a1* siRNA.

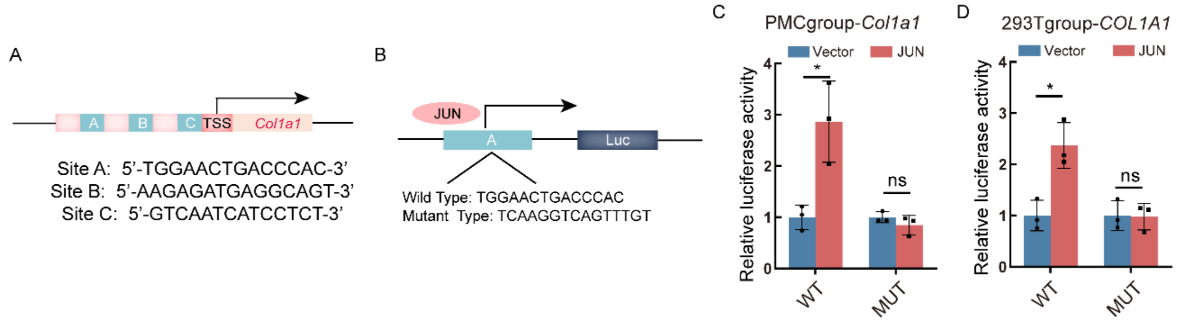

Figure S16. JUN was a transcription factor for type I collagen. (A) Prediction of JUN-binding sites in *Col1a1* promoter. (B) Schematic diagram of mutation at site A of *Col1a1* promoter and its sequence. (C, D) Luciferase activity analysis of reporter plasmids containing wild-type or mutant *Col1a1* promoter binding sites with JUN plasmid administration in rat PMCs (C) and HEK293T cells (human) (D). Data were expressed as mean±SD. Statistical significance was determined by one-way ANOVA. n=3. *$P < 0.05$.

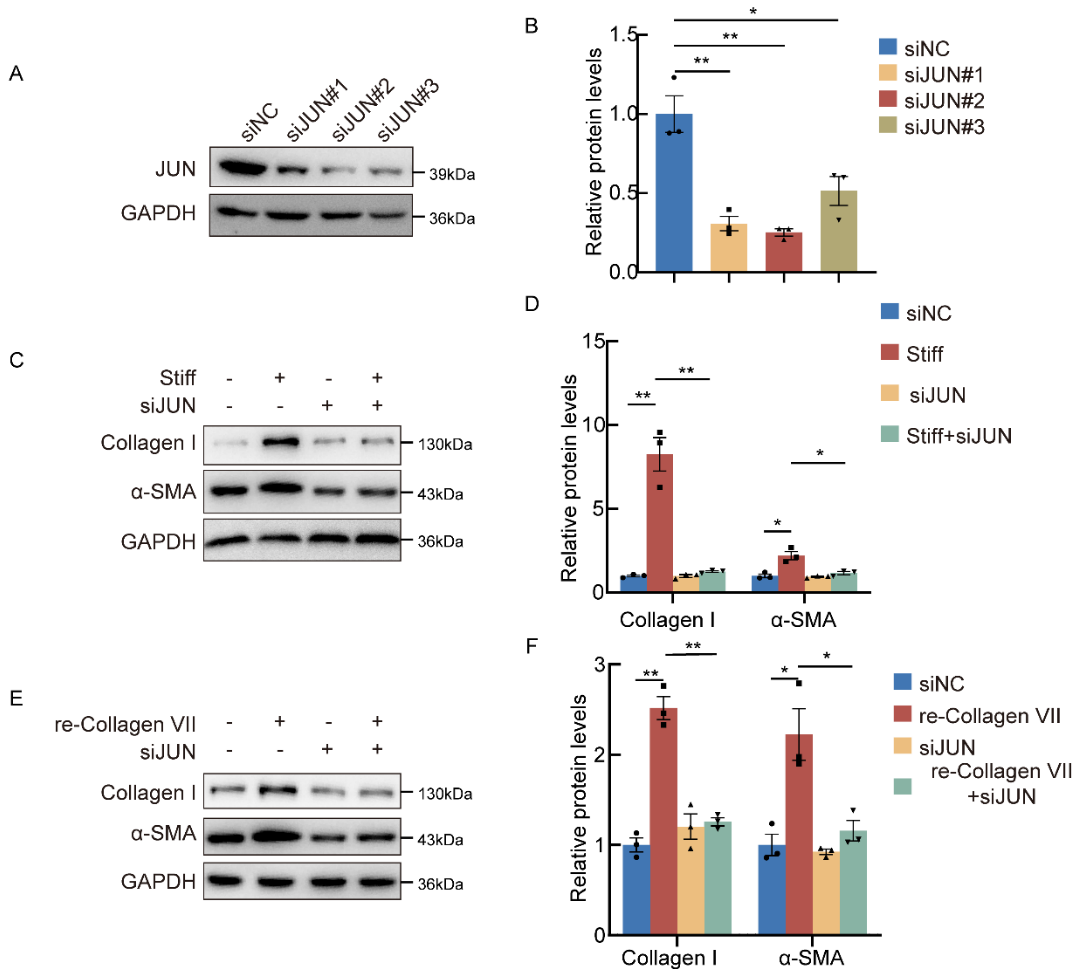

Figure S17. *Jun* siRNA prevented up-expression of collagen I and α-SMA induced by recombinant collagen VII protein and stiff matrix. (A, B) Three *Jun* siRNAs were designed and evaluation their silencing efficiency by western blotting. J*un* siRNA2 in A was selected to do subsequent experiments. Data were expressed as mean±SD. Statistical significance was determined by one-way ANOVA. n=3. **P* < 0.05, ***P* < 0.01. (C, D) Stiff matrix medium was made by Matrigel at concentrations 20 mg/ml which was as same as description in Figure 6C. The control siRNA or *Jun* siRNA was transfected into primary rat PMCs for 36 h, then PMCs were cultured in control or stiff matrix for 24 h. Cells were harvested for western blotting. (E, F) After transfection control siRNA or *Jun* siRNA for 36 h, PMCs were cultured in the medium with or without recombinant collagen VII protein for 24 h. Cells were harvested for western blotting. (C, E) Representative images of western blots. (D, F) Bar graphs showing changes of proteins according to C, E. Data were expressed as mean±SD. Statistical significance was determined by one-way ANOVA. n = 3. **P* < 0.05, ***P* < 0.01. siNC: control siRNA. siJUN: *Jun* siRNA. re-Collagen VII: recombinant collagen VII protein.

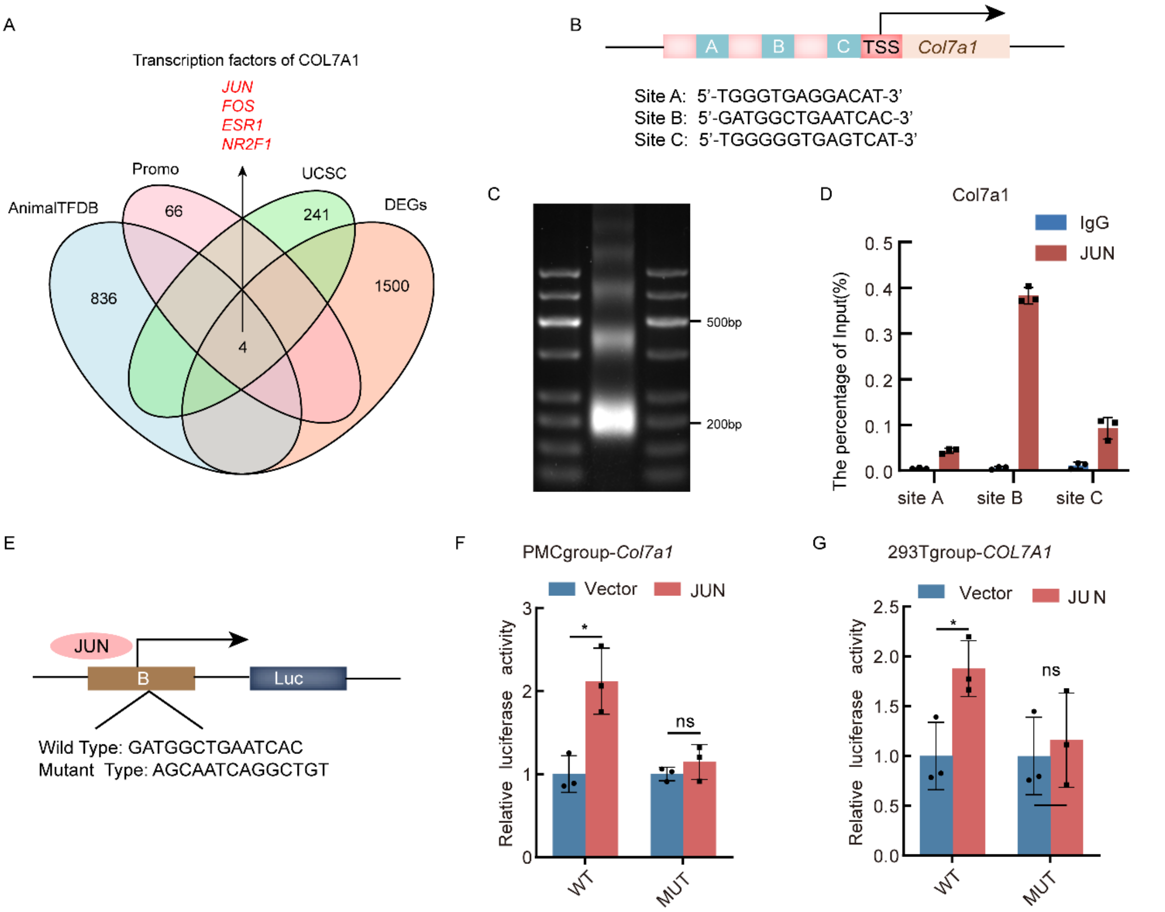

Figure S18. JUN activated transcription of the gene of collagen VII. (A) Venn diagram showing prediction of transcription factors for *Col7a1*. Based on data of differentially expressed genes after collagen VII knockdown (showing in Figure 6A), transcription factors for *Col7a1* were predicted using three transcription factor databases (Animal TFDB, Promo, UCSC). (B) Prediction of JUN-binding sites for *Col7a1* promoter region. (C) Assessment of shearing efficiency by agarose gel electrophoresis of DNA products from chromatin immunoprecipitation (ChIP). (D) ChIP-qPCR to evaluate binding activity of JUN at three sites on *Col7a1* promoter. (E) Schematic diagram of mutation at site B of *Col7a1* promoter and its sequence. (F, G) Luciferase activity analysis of reporter plasmids containing wild-type or mutant JUN binding sites in *Col7a1* or *COL7A1* promoter after transfected with JUN plasmid or vector plasmid in rat PMCs (F) and HEK293T cells (G). Data were expressed as mean±SD. Statistical significance was determined by one-way ANOVA. n = 3. *P < 0.05, ns: not significant.

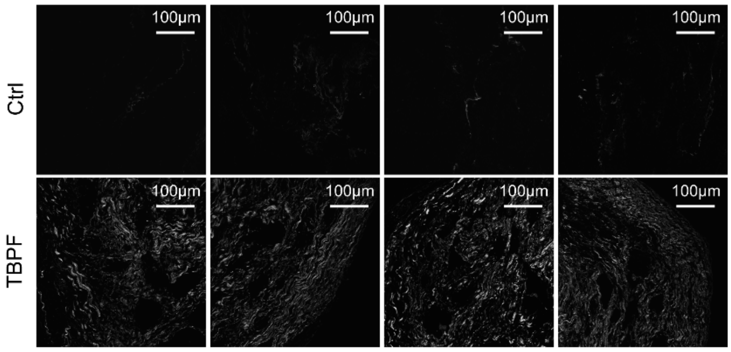

Figure S19. Second Harmonic Generation (SHG) validation of human pleural tissue samples. As described in the Methods, human pleural tissues were obtained as the same as that in the Figure 1. Representative SHG images showing collagen deposition in pleural tissues. Ctrl: pleural tissues from control subjects. TBPF: pleural tissues from patients with tuberculous pleural fibrosis. Scale bars, 100 μm.

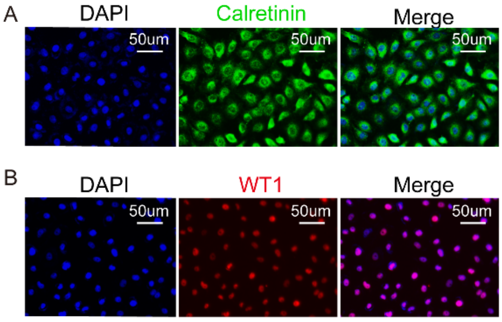

Figure S20. Identification of primary human pleural mesothelial cells (HPMCs). As described in the Methods, primary HPMCs were isolated from pleural effusion. Immunofluorescence staining was performed to reveal expression of mesothelial cell markers calretinin (green) and WT-1(red). Nuclei were counterstained with DAPI (blue).
