# Supplementary material for: Excessive collagen type VII mediates pleural fibrosis via increasing extracellular matrix stiffness
Source: J Clin Invest. 2025 Oct 16;135(24):e188822. doi: 10.1172/JCI188822 (PMC12700545; doi:10.1172/JCI188822)

F2 D

Collagen VII

Collagen I

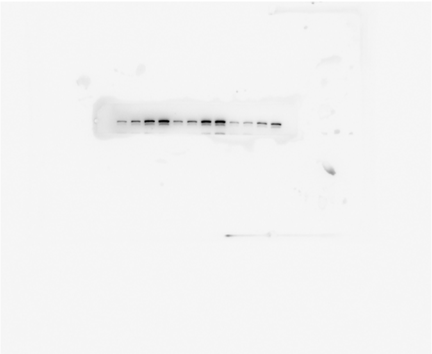

α-SMA

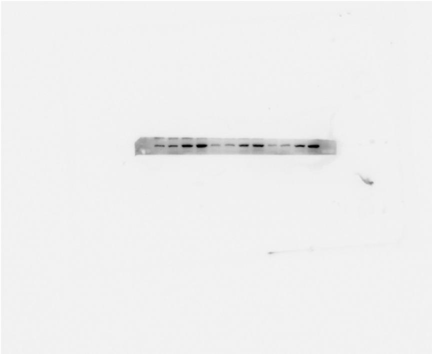

GAPDH

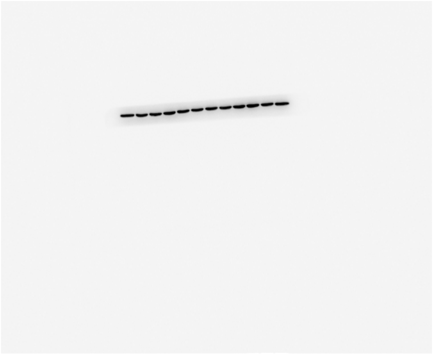

F4 G

Collagen I

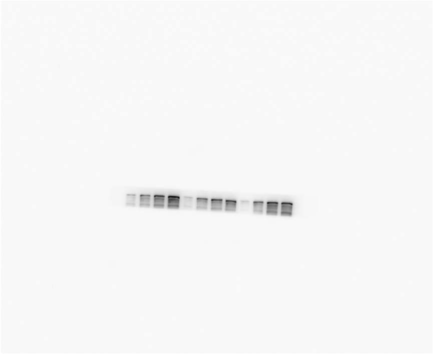

α-SMA

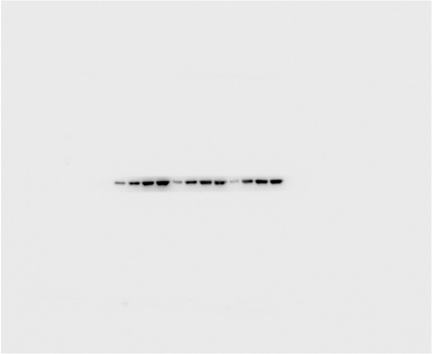

GAPDH

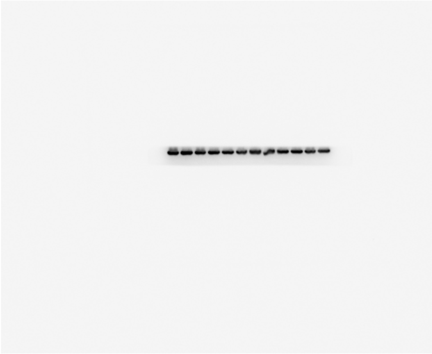

F6 D

Collagen I

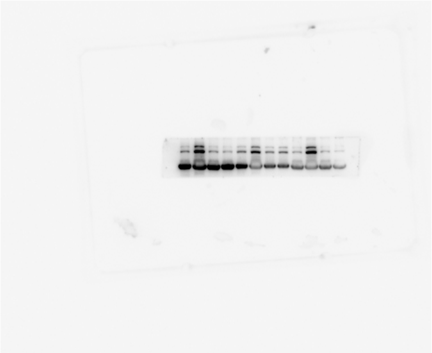

α-SMA

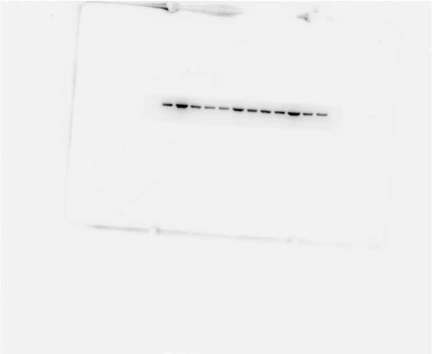

GAPDH

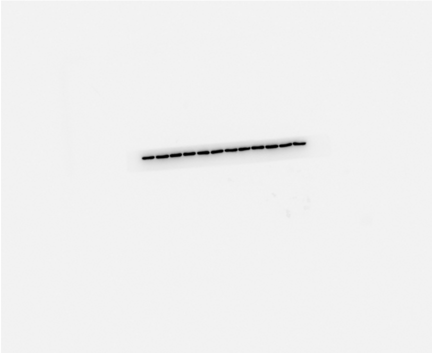

F6 F

Collagen I

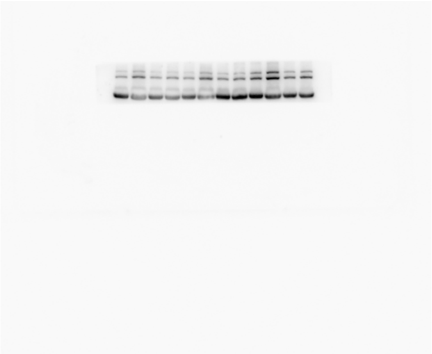

α-SMA

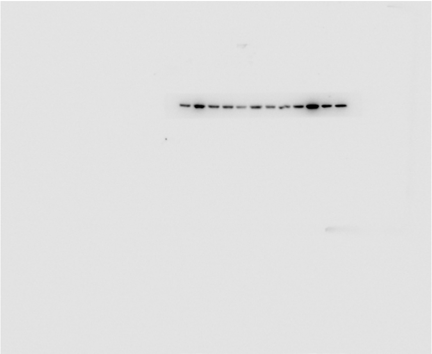

GAPDH

F6 H

Collagen I

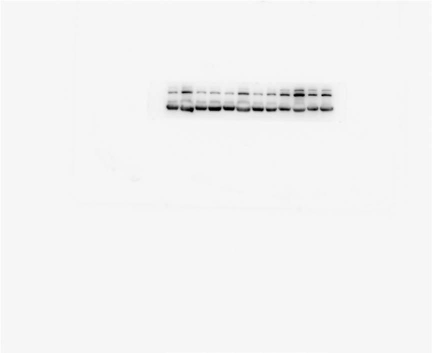

α-SMA

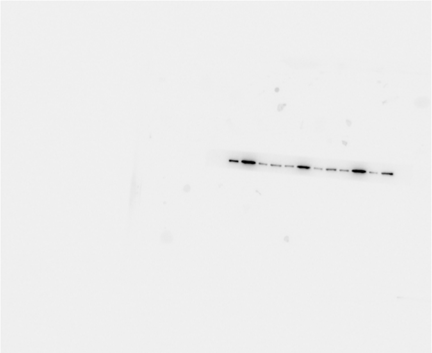

GAPDH

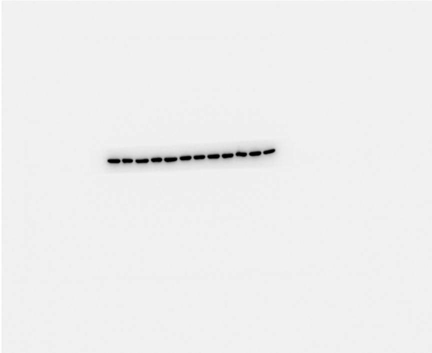

F7 C

p-PI3K
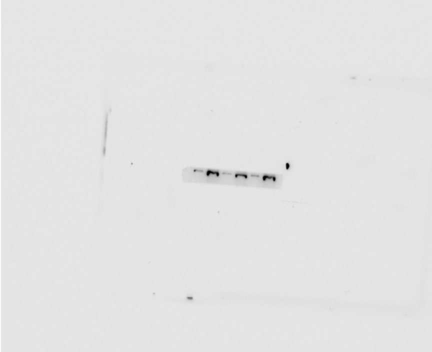
PI3K
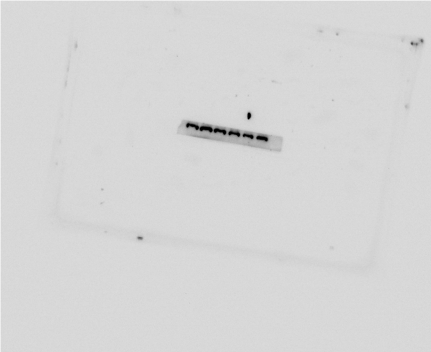
pS473-AKT
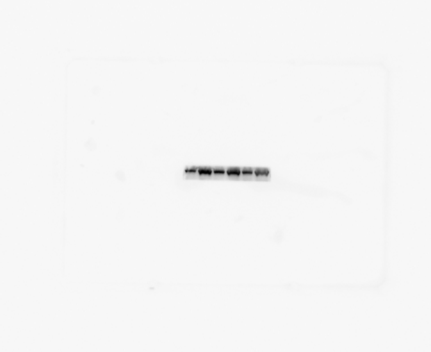
AKT
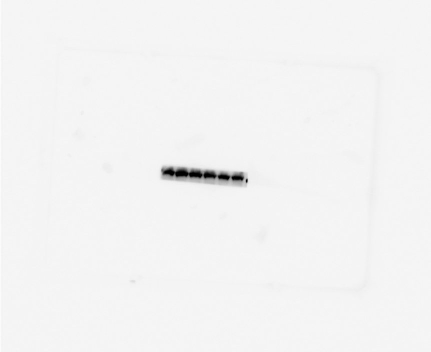
GAPDH
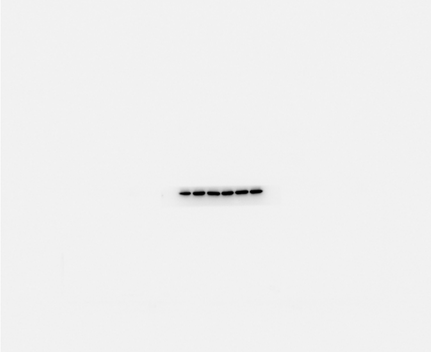

F7 E

p-PI3K
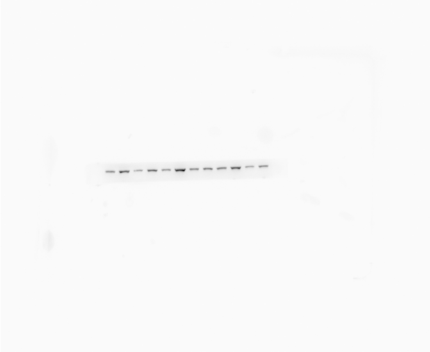
PI3K
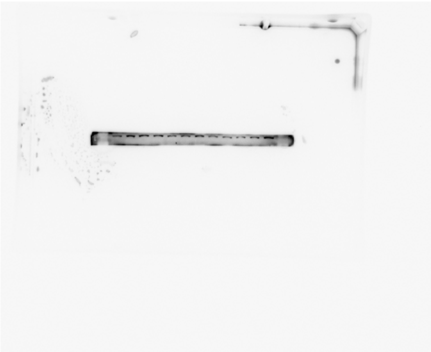
pS473-AKT
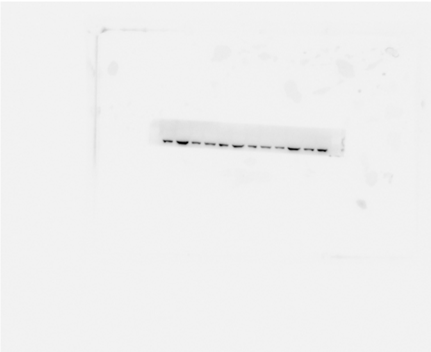
AKT
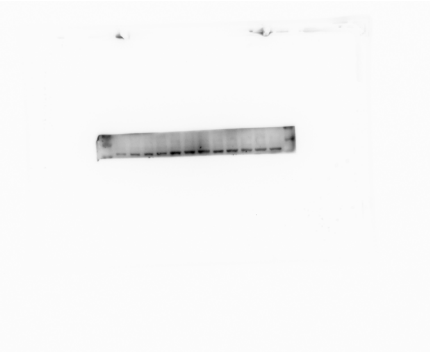
GAPDH
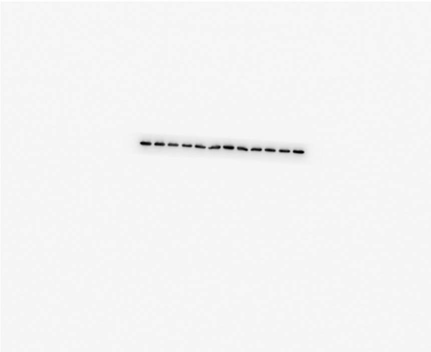

F7 G

p-PI3K
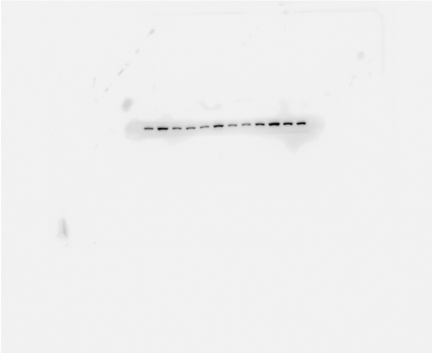
PI3K
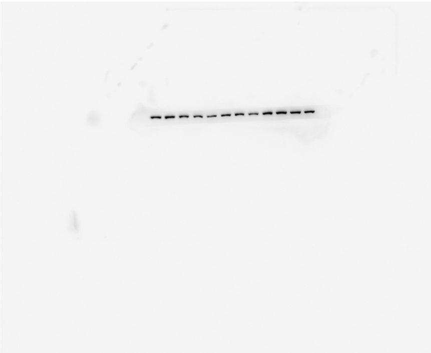
pS473-AKT
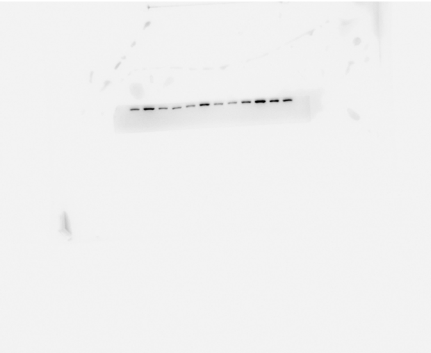
AKT
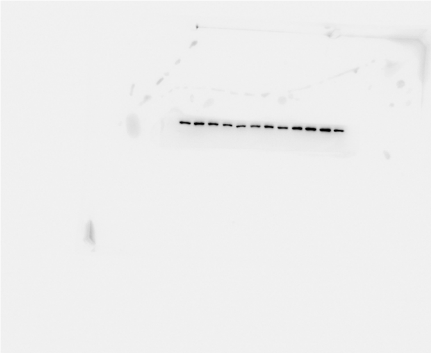
GAPDH
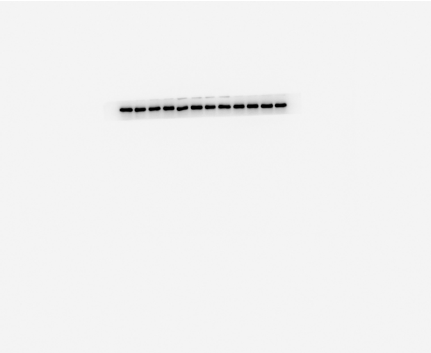

F7 I

p-PI3K

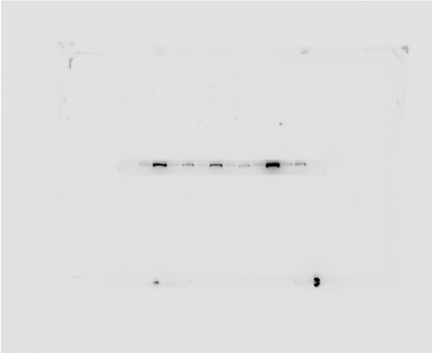

PI3K

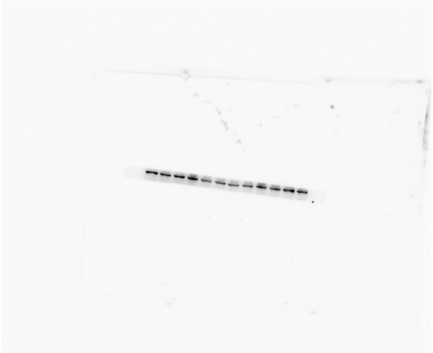

pS473-AKT

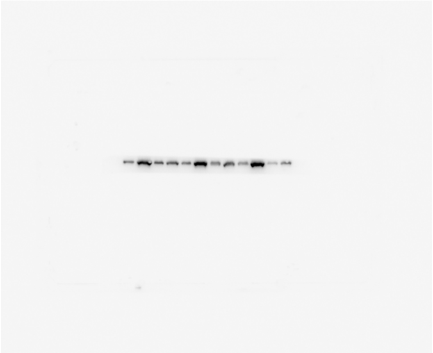

AKT

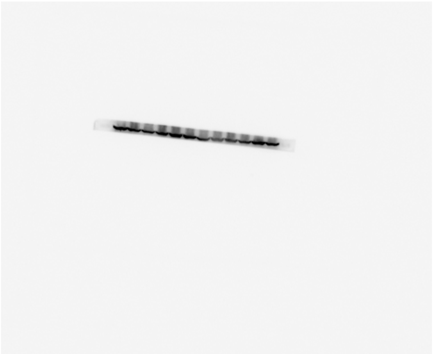

GAPDH

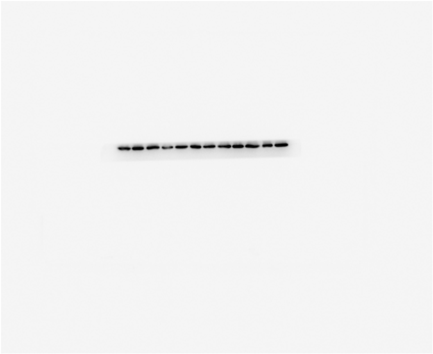

F8 C

JUN

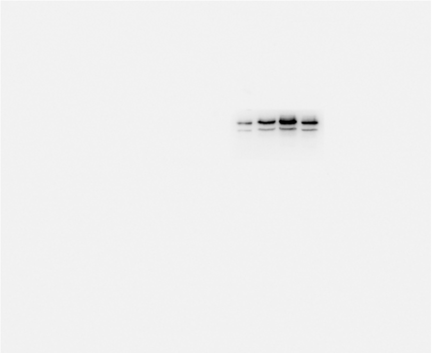

H3

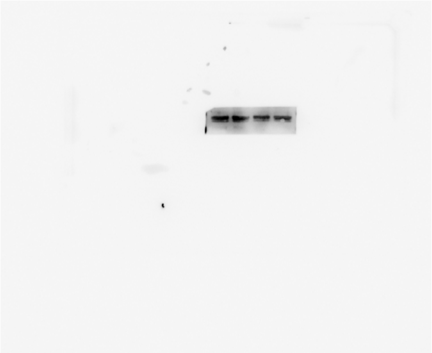

FS1 G

Collagen VII

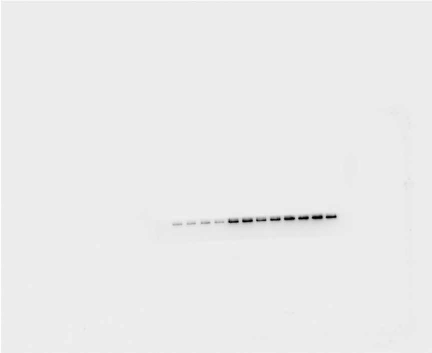

GAPDH

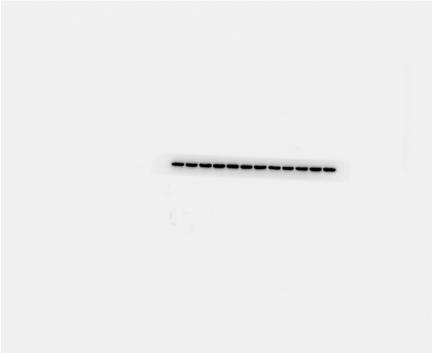

FS3 A

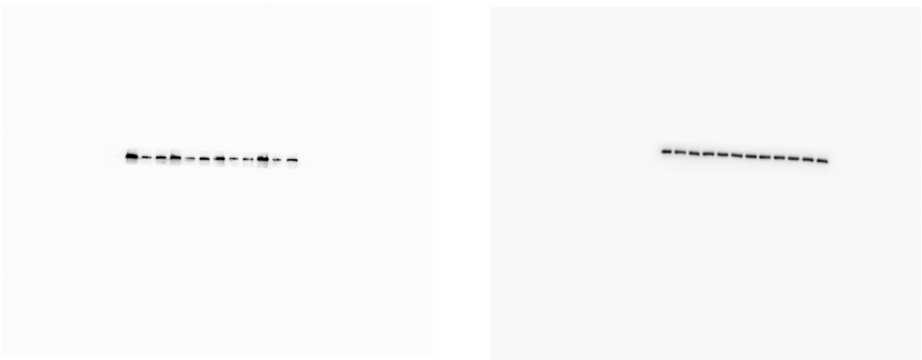

FS3 C

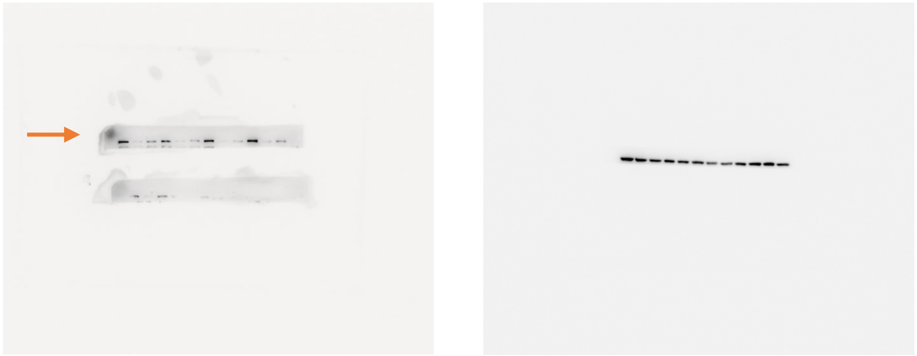

FS3 E

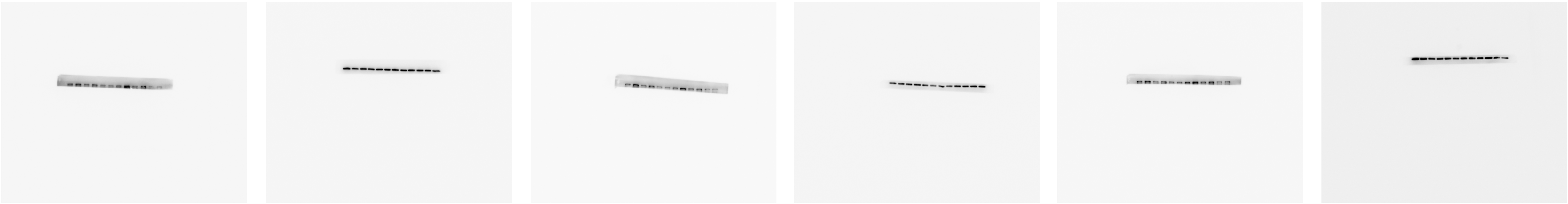

FS3 G

Collagen VII

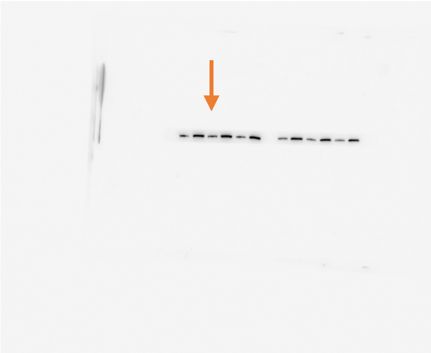

GAPDH

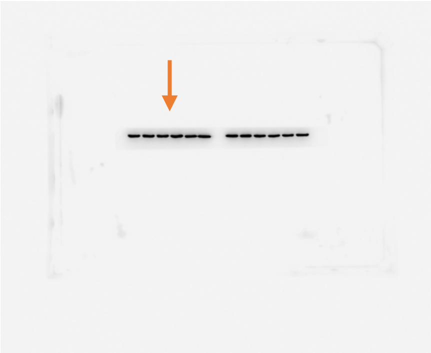

FS3 I

Collagen VII

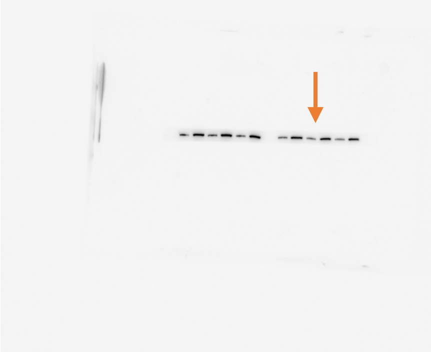

GAPDH

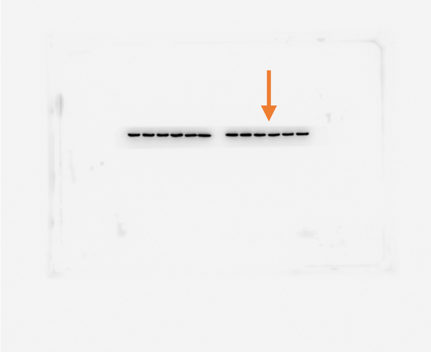

FS4 C and E

Collagen VII

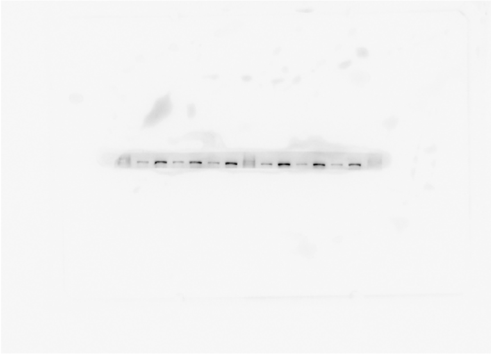

GAPDH

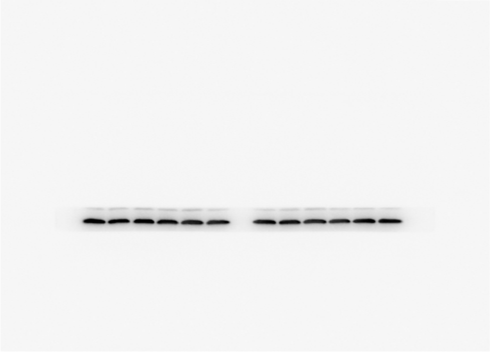

FS4 I

Collagen VII

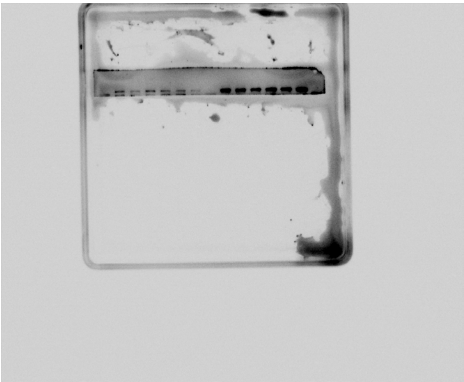

GAPDH

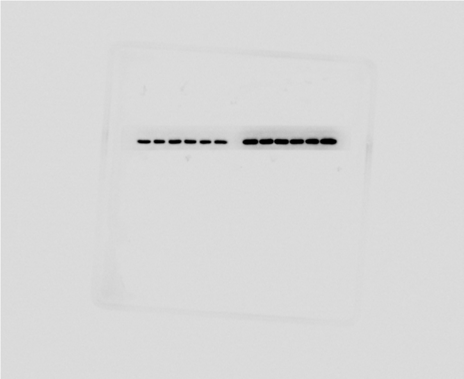

FS5 A

Collagen VII

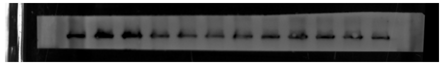

GAPDH

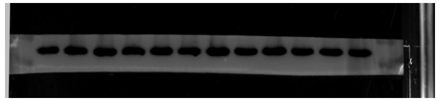

FS5 F

Collagen I

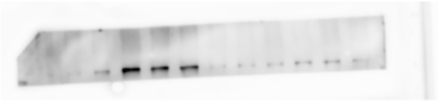

α-SMA

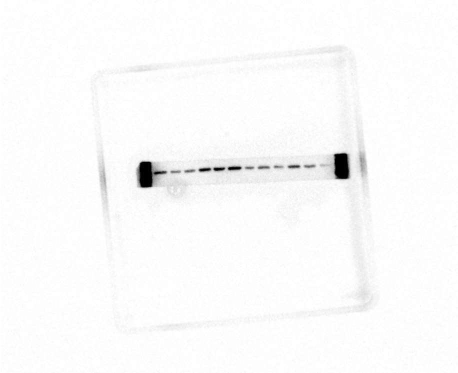

GAPDH

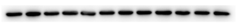

FS5 I

Collagen I

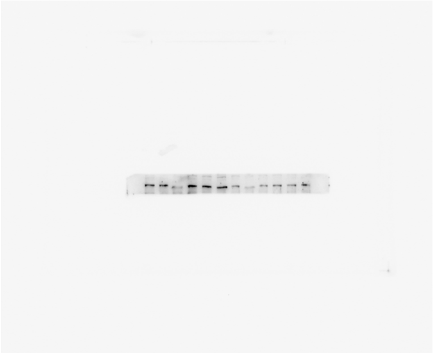

α-SMA

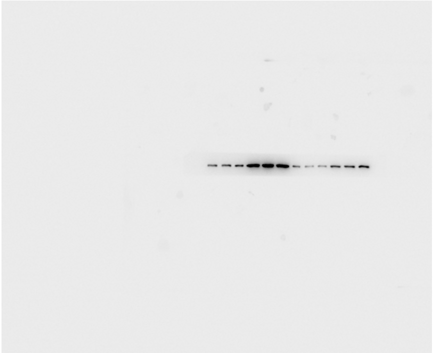

GAPDH

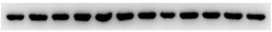

FS6 A

Collagen I

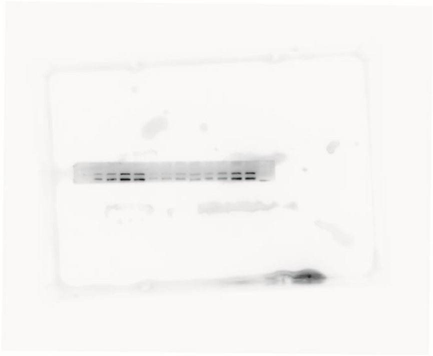

α-SMA

GAPDH

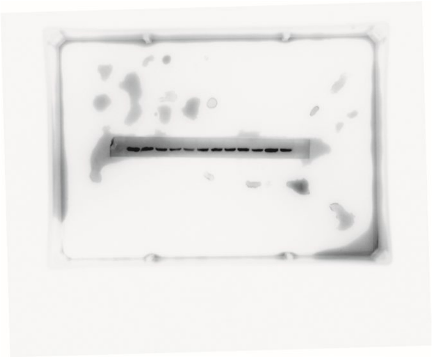

S7 A

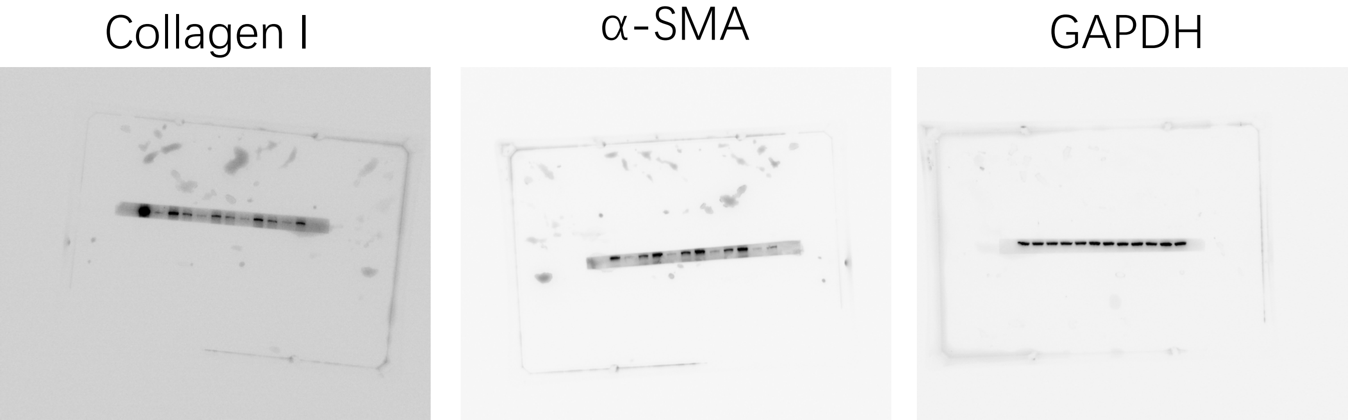

S11 A

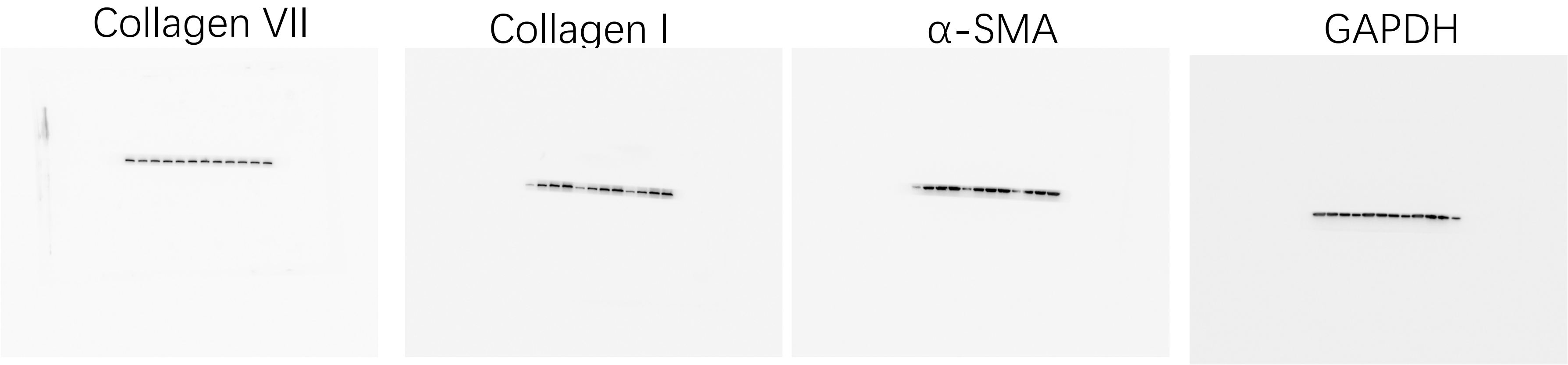

S12 A

Collagen I | α-SMA | GAPDH

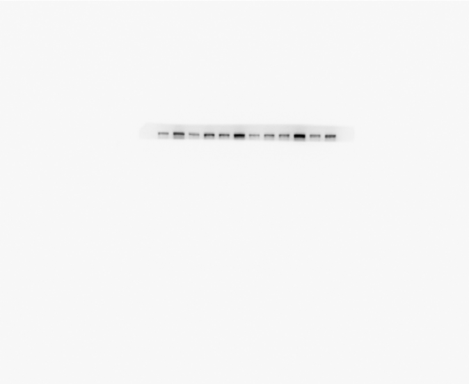

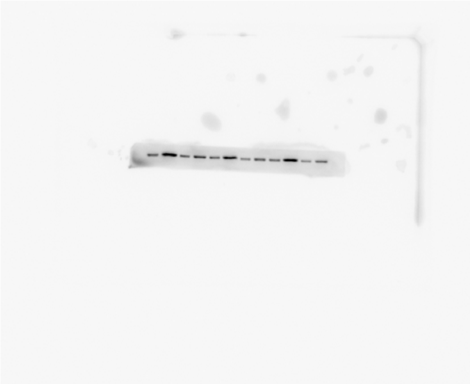

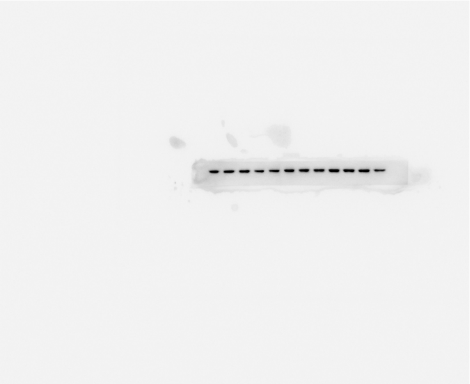

S12 C

Collagen I | α-SMA | GAPDH

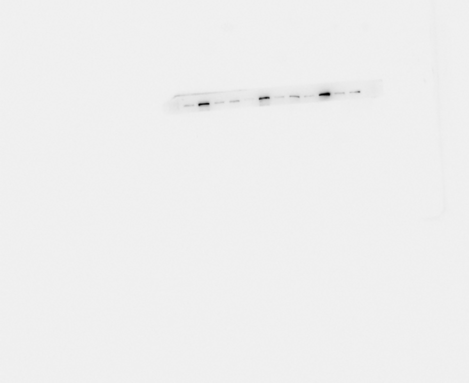

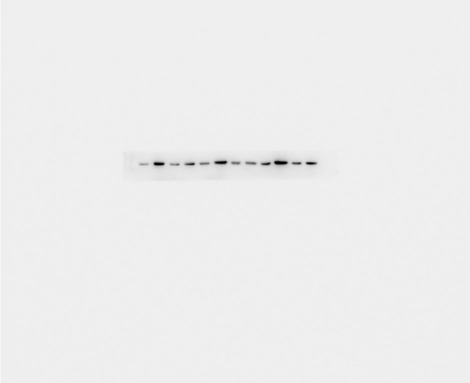

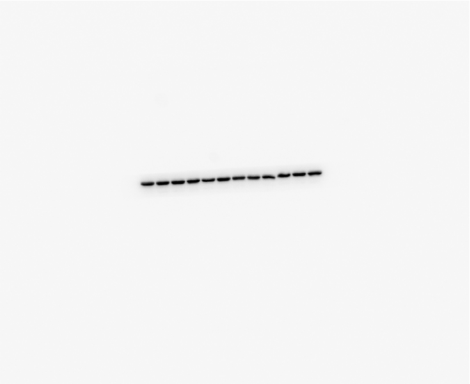

S12 E

Collagen I | α-SMA | GAPDH

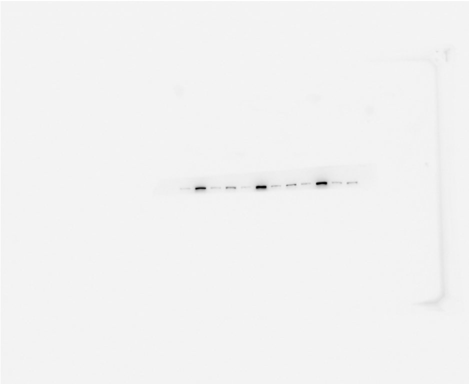

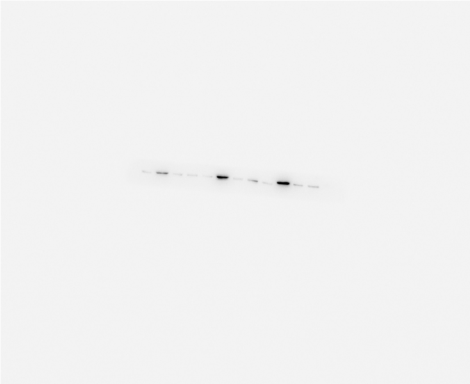

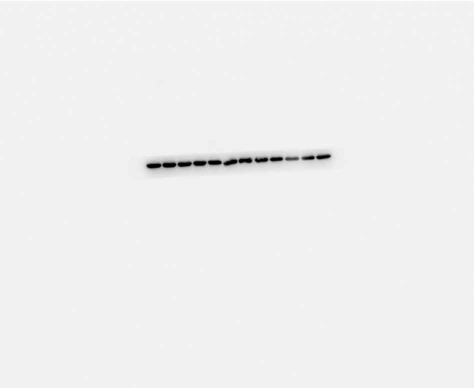

S13 A and C

p-PI3K

PI3K

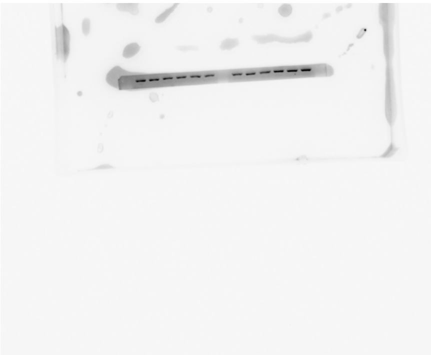

pS473-AKT

AKT

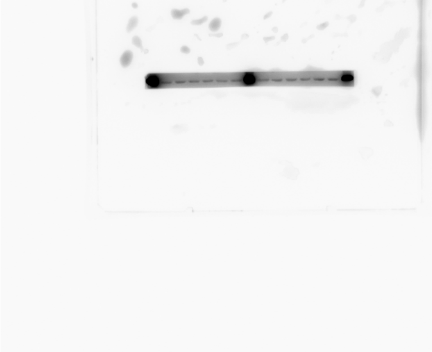

GAPDH

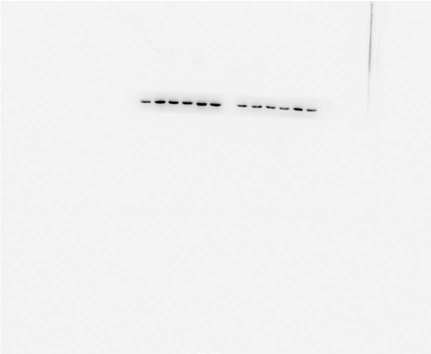

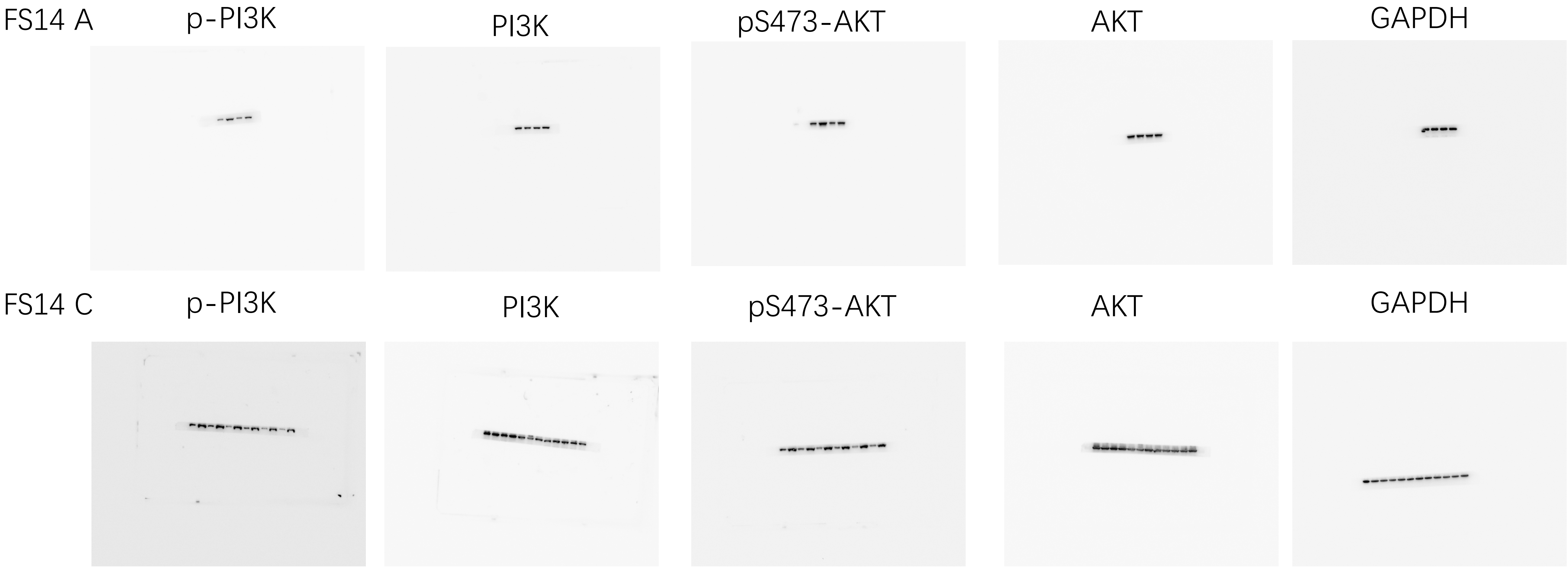

FS14 A
p-PI3K
PI3K
pS473-AKT
AKT
GAPDH
FS14 C
p-PI3K
PI3K
pS473-AKT
AKT
GAPDH

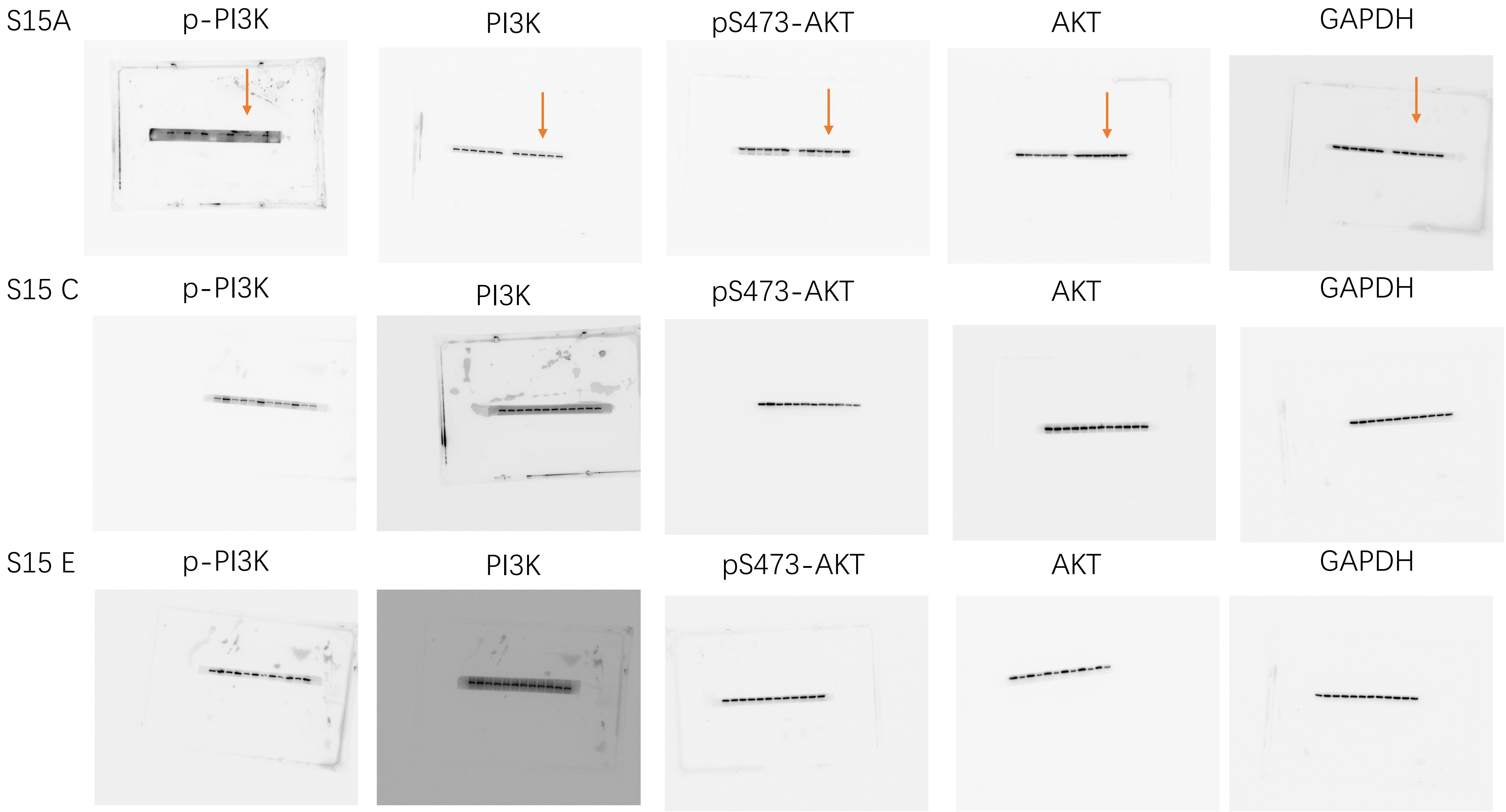

S15A
p-PI3K
PI3K
pS473-AKT
AKT
GAPDH
S15 C
p-PI3K
PI3K
pS473-AKT
AKT
GAPDH
S15 E
p-PI3K
PI3K
pS473-AKT
AKT
GAPDH

S17 A

JUN

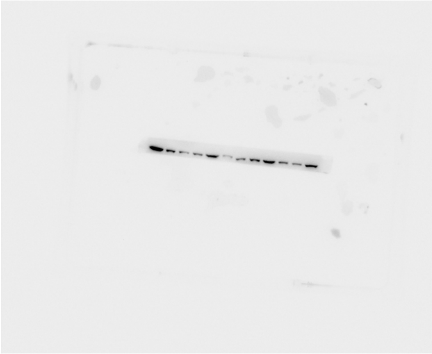

GAPDH

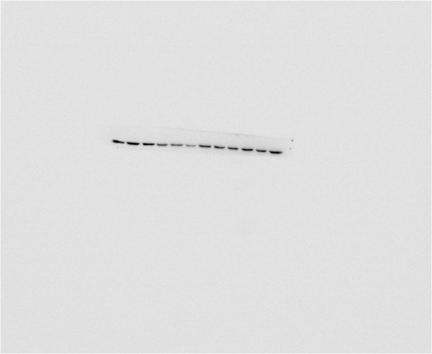

S17 C

Collagen I

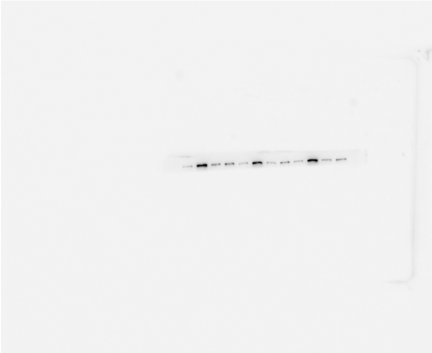

α-SMA

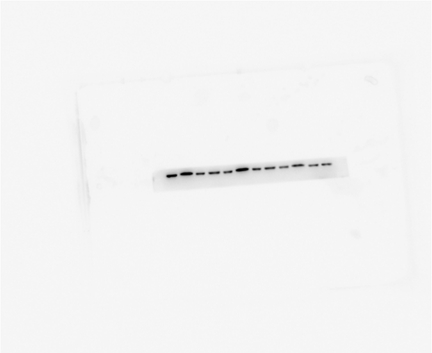

GAPDH

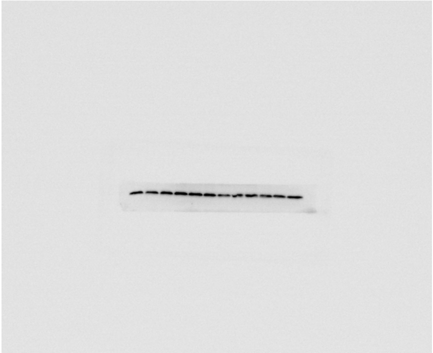

S17 E

Collagen I

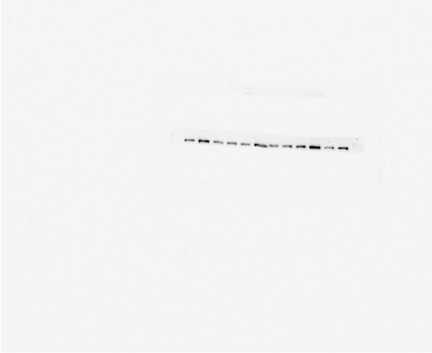

α-SMA

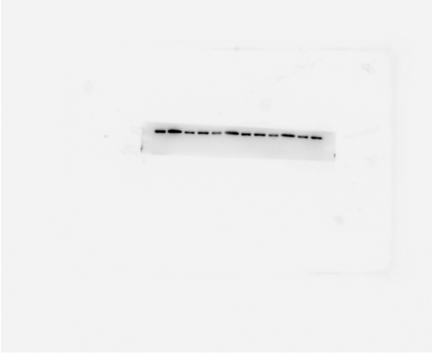

GAPDH

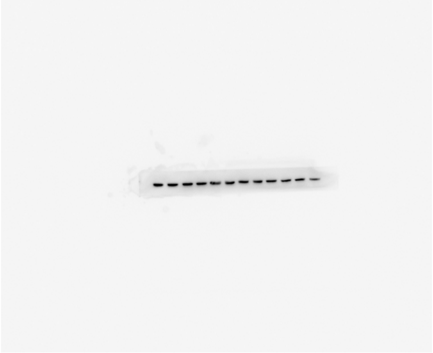

Supplement: Unedited blot and gel images [file jci-135-188822-s061.pdf]
